# A Hybrid Approach for Brain-Tumor Detection and Classification from MRI Images

**DOI:** 10.3390/jimaging12090460

**Published:** 2026-09-21

**Authors:** Mohammad Shahjahan Majib, Md. Mamun Or Rashid, Md. Ashraful Haque, Faysal Ahmed, T. M. Shahriar Sazzad

**Affiliations:** 1 Department of Computer Science and Engineering, Military Institute of Science and Technology (MIST), Dhaka 1216, Bangladesh; smajib@yahoo.com (M.S.M.); faysal202864@gmail.com (F.A.); 2 Department of Computer Science and Engineering, University of Dhaka, Dhaka 1000, Bangladesh; mamun@cse.du.ac.bd; 3 Department of Neurosurgery, National Institute of Neuroscience Hospital, Dhaka 1207, Bangladesh; dr.ashraful2002@gmail.com

**Keywords:** brain-tumor classification, magnetic resonance imaging (MRI), hybrid ensemble learning, temperature scaling (probability calibration), stacking (logistic meta-learner), transfer learning (multi-backbone)

## Abstract

Many existing deep-learning models for brain-tumor diagnosis report very high accuracy, yet they often suffer from overfitting, limited generalizability, and poor robustness, which reduces their clinical reliability. First, a large and diverse dataset was assembled from multiple publicly available sources to reduce data bias. Next, a series of regularization and calibration techniques were applied, including label smoothing, mixup augmentation (α=0.4), exponential moving average (EMA) of model weights to prevent overfitting, stabilize training, and temperature scaling to calibrate predicted probabilities. Since brain-tumor diagnosis directly affects critical clinical decisions, both model accuracy and reliability of its predictions are essential. For that, six diverse, powerful pretrained backbones (ConvNeXt, EfficientNetV2, DenseNet201, Swin, ViT, and DINOv3) were used as frozen feature extractors under pure transfer learning. On top of these frozen features, lightweight classification heads were trained using concatenation, attention, and gated fusion. Then, the predicted-class probabilities from the selected heads were combined and passed to a logistic-regression meta-learner to produce the final prediction, with the aim of improving accuracy and reliability. This architecture is computationally efficient because only lightweight classifiers and a logistic meta-learner are trained, while the heavy backbones remain frozen. The best-performing variant on TEST2, attn_fp_ens, was further evaluated using synchronized 10-fold cross-validation on the TRAIN set. The best-performing TEST variant, the gated-fusion ensemble with fingerprint features (gated_fp_ens), achieved 99.33% test accuracy and 99.28% macro-F1. Under domain shift on the external hospital dataset (TEST2), the attention-fusion ensemble with fingerprint features (attn_fp_ens) showed the strongest performance among the evaluated variants, achieving 95.16% accuracy and 94.11% macro-F1. The TEST2 dataset was collected from a local hospital for external evaluation.

## 1. Introduction

Magnetic resonance imaging (MRI) is widely used for brain-tumor detection due to its excellent soft-tissue contrast and non-invasive nature [1,2]. In recent years, deep learning has significantly advanced neuro-oncological imaging by enabling automated extraction of features for tumor classification [1]. Recent deep-learning models report strong performance for brain-tumor MRI classification [3,4]. However, these gains often come with limitations. Many models tend to overfit on relatively small datasets, lack generalizability, and have poor reliability in real-world clinical settings [3,5]. High accuracy on a single dataset does not guarantee robust performance across diverse imaging conditions or institutions. Moreover, many deep models are miscalibrated, producing overconfident predictions that undermine clinicians’ trust [6,7]. In the context of brain tumors, where diagnostic errors can have life-or-death consequences, it is essential to develop models that are accurate, generalizable, robust, and well-calibrated.

These challenges are addressed by integrating data diversity, regularization, and model hybridization into the classification pipeline. First, a large-scale MRI dataset was constructed by aggregating multiple public sources to capture a wide variety of tumor appearances and imaging protocols. This dataset is larger and more diverse than those typically used in related studies, allowing the model to learn from a broader range of tumor types and imaging conditions. Training on such diverse data enhances model generalization and reduces overfitting [5,8]. Second, strong regularization and calibration techniques were employed during training, including label smoothing to prevent overconfident outputs, mixup augmentation to enrich data variability [9], and an exponential moving average (EMA) of model weights to stabilize learning [10]. These strategies are widely used to reduce overfitting and improve generalization [9,10,11,12].

The approach further applied temperature scaling as a post-hoc calibration, which significantly reduces the prediction confidence error (ECE) without sacrificing accuracy [7]. Finally, a hybrid ensemble model was created that incorporates the complementary strengths of multiple architectures. Six diverse backbones were integrated as feature extractors, each pretrained on large-scale datasets. On top of these, lightweight classification heads were trained and then stacked in an ensemble configuration to aggregate their predictions. This stacked ensemble (using a meta-learner) provides a robust final prediction by combining multiple perspectives on the data. By design, this approach improves accuracy and reliability while keeping computational costs reasonable due to the efficiency of transfer learning. The approach was validated using rigorous 10-fold cross-validation and external testing, demonstrating that the proposed method achieves strong accuracy while maintaining generalization.

While previous studies have successfully applied hybrid or ensemble methods using a random approach, this work stands out by systematically exploring a design space of hybridization within a unified and leakage-free training protocol. The innovation here is not in proposing a new standalone architecture but in offering a reproducible, calibration-aware, and generalization-focused framework.

### Contributions

- This study presents a systematic methodology for building an integrated and leakage-aware hybrid model for the classification of brain tumors from MR images, incorporating multiple backbone feature extractors, fusion strategies, and probability calibration in one consistent pipeline.
- Extensive evaluation of 28 fusion variations (concatenation, attention-based, gating mechanisms, etc.) was performed in a well-controlled environment, contributing to a better understanding of the performance of various fusion schemes.
- A stacking method based on OOF predictions is developed, allowing unbiased meta-learning and preventing potential data-leakage problems that have been frequently ignored by other ensemble-based methods.
- An integrated pipeline with learning-aware calibration, such as temperature scaling, ECE, and Brier score, is proposed, emphasizing the significance of accurate probability assessment along with classification performance.
- This proposed approach is tested not only on internal but also external datasets, including a hospital dataset, to address domain-shift issues.

## 2. Literature Review

Deep learning has become a standard approach for automated brain-tumor classification from MRI and has shown strong performance in many recent studies. Prior work has explored a wide range of architectures, from traditional convolutional neural networks (CNNs) to more recent vision transformers. In particular, ensemble learning has often provided additional gains. For example, Liu et al. (2025) evaluated seven CNN-based models, including ResNet, GoogLeNet, MobileNet, and EfficientNet, and reported that a majority-vote ensemble achieved 99.8% classification accuracy, outperforming any single model [3]. The authors further observed that model diversity improved robustness and reduced the risk of overfitting [3].

Recent studies have also reported high accuracy on MRI-based datasets using attention-enhanced designs. For instance, Balamurugan et al. (2024) combined ResNet101 with a channel-wise attention module (CWAM) and achieved 99.83% accuracy for a four-class brain-tumor classification task [4]. Similarly, attention-based ensemble strategies have been investigated and reported strong performance on BraTS-style benchmarks, while also improving inference efficiency [13]. Ullah and Kim (2025) proposed a hierarchical deep-feature fusion and ensemble-learning framework for brain-tumor MRI classification, demonstrating the effectiveness of combining complementary pretrained features with ensemble learning [14].

A systematic review by Bouhafra and El Bahi summarized 60 studies published between 2020 and 2024, covering CNNs, transfer learning, autoencoders, transformers, and attention mechanisms [1]. Recent approaches and their reported results are summarized in Table 1.

Due to the scarcity of medical imaging data, transfer learning has become a foundation of most approaches. Pretrained models trained on extensive natural-image datasets are fine-tuned on brain MRI datasets to achieve high performance [4,21]. CNNs extract features automatically from MRIs, enabling precise classification of brain tumors [2,21]. In addition, ensemble strategies are frequently employed to further enhance performance, because combining multiple architectures can integrate their respective strengths and improve robustness [21]. Recent research has increasingly addressed multiclass classification challenges, often using datasets like the Figshare dataset (which includes glioma, meningioma, and pituitary tumors) and Kaggle-style datasets that include a “no-tumor” class [21]. Hybrid models further expand the design space, combining architectures like CNNs with transformers, CNNs with recurrent neural networks (RNNs), such as CNN + LSTM, and deep features paired with classical machine learning classifiers like SVM, Random Forest, and XGBoost. Many studies have reported strong results on benchmarks using these hybrid approaches [21]. Stacking combines the predictions of multiple models and can provide better performance than simple voting. Recent explainable stacking approaches have also shown improved robustness by combining complementary features from different models [5].

Even though many studies report impressively high accuracies, there are still real concerns about overfitting and how well these models actually generalize. Often, models are only tested on data that comes from the same source they were trained on (i.e., a single dataset split), which can give an overly optimistic picture and does not guarantee real-world performance. As highlighted in systematic reviews, many studies do not include strong external validation, even though it is crucial for clinical reliability [1,21]. Singh et al. (2025) found that CNN models, even those with high accuracy, often fail to generalize to new data [22]. Some studies have used cross-dataset validation to address this. For example, Liu et al. (2025) tested their ensemble on an independent external dataset [3]. They found that single models performed worse, while the ensemble was more robust [3]. However, cross-dataset testing is still not common, and many studies lack cross-validation and external validation, which remains a key concern [1,21].

A practical issue is that model size and efficiency can strongly affect real-world usability. A comparative study reported that NASNetLarge (about 84.9M parameters) achieved 97.56% accuracy but required a long training time (1188 min) and raised concerns about computational cost and overfitting risk, whereas MobileNetV2 (about 2.2M parameters) achieved over 95% accuracy with much faster training (101 min), offering a better balance in resource-limited settings [23].

### Research Gap

Although various hybrid/ensemble methods have been employed for brain-tumor classification using deep models, existing literature fails to conduct a comparative analysis among various fusion methods, which makes it hard to establish which methods are effective. Moreover, most existing literature has employed fusion methods in an ad hoc manner, i.e., either using concatenation or averaging methods without conducting a comparative analysis among other methods, such as attention or gated fusion. In addition, most existing literature has not employed stacking methods that are safe against leakage, i.e., without using any predictions generated using any data that has been used for meta-learning.

Furthermore, calibration analysis with metrics like Expected Calibration Error (ECE) and Brier score is seldom employed in these studies, despite its critical necessity in clinical decision-making, where reliability in confidence is equally important to accuracy. Moreover, most studies are based only on internal datasets and not validated with external clinical data, which raises questions about domain shifts in generalized settings. Therefore, the robustness of these models in practical settings is questionable. To overcome these shortcomings, a unified and systematically evaluated framework is necessary to not only compare different fusion strategies in a controlled environment but also ensure leakage-free evaluation and calibration analysis with external validation using clinical data.

## 3. Methodology

**Figure 1:**
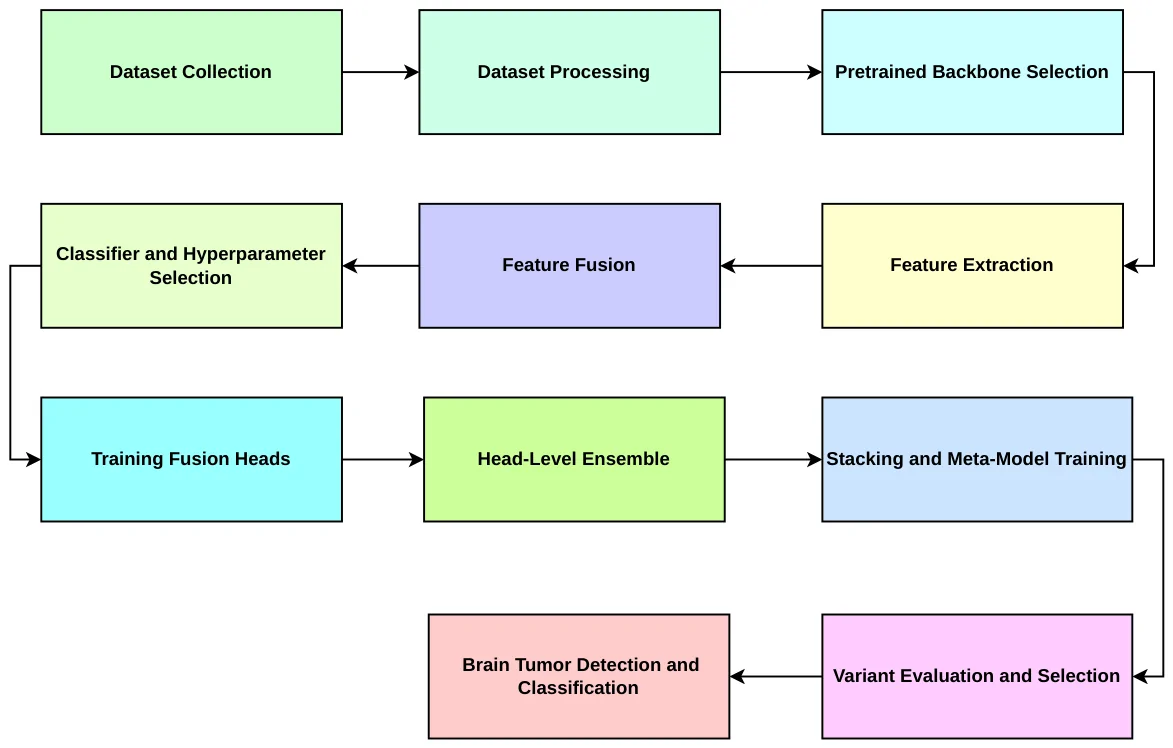
Overview of the research methodology.

Figure 1 provides an overview of the methodology.

### 3.1. Dataset Description and Preprocessing

**Figure 2:**
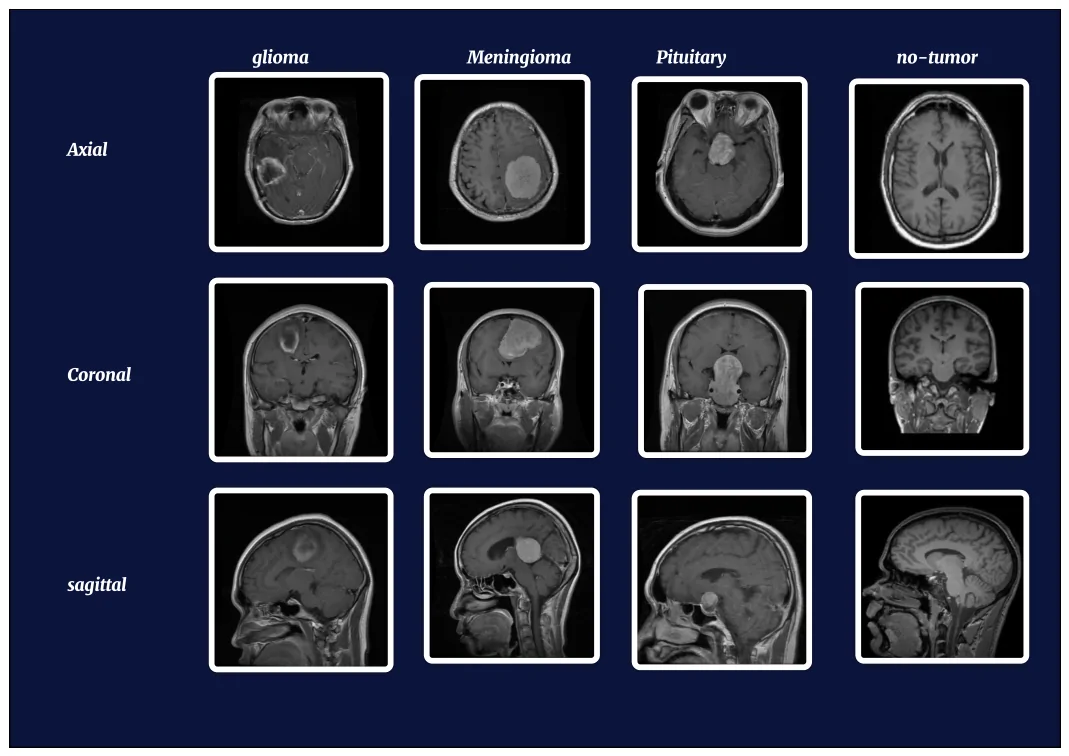
Illustration of sample brain-tumor images from the merged public dataset.

The dataset was constructed by merging nine publicly available brain MRI collections (Table 2). After merging, the raw combined dataset contained 77,719 images. Sample images from the dataset are shown in Figure 2.

#### 3.1.1. Handling Data Redundancy and Leakage Prevention

Public MRI repositories often reuse images across different uploads. As a result, the same MRI scan may appear multiple times, sometimes with a different filename, file format, or compression level. If these duplicates are present across different splits (e.g., training and testing), a model can indirectly “see” the test data during training. This causes data leakage and leads to overly optimistic performance.

To reduce this risk, an exact duplicate and near-duplicate removal pipeline was applied to the merged dataset. All images were first collected from class-wise folders. Three complementary matching strategies were then used:

(i) Exact file duplicates: A SHA-256 hash was computed from the raw file bytes. Files with identical SHA-256 values were treated as exact duplicates.

(ii) Pixel-level duplicates: Each image was converted to RGB, resized to 256×256, and an MD5 hash was computed from the resulting pixel bytes. This step identifies visually identical images that may differ only due to metadata, compression, or file encoding.

(iii) Near-duplicates: A 64-bit perceptual hash (pHash; hash size =8) was computed for each image. To enable efficient search, a locality-sensitive hashing (LSH) scheme was used by splitting the 64-bit hash into four 16-bit segments and grouping images that matched within any segment. Candidate pairs were compared only within these buckets, and two images were considered near-duplicates if their pHash Hamming distance was ≤3.

Using these criteria, exact duplicates, pixel-level duplicates, and near-duplicate images were identified. A conservative cleaning rule was applied: if a duplicate group appeared across multiple classes, the entire group was removed to avoid label conflicts; otherwise, one representative image (the largest file) was retained, and the remaining copies were discarded. After cleaning, 20,794 images remained, and this leakage-controlled dataset was used for all experiments. The class-wise distribution of training, validation, and test splits is presented in Table 3.

#### 3.1.2. External Test Set (TEST2)

An additional dataset (TEST2) was used only for external evaluation. It was not used during model training. The dataset contains 682 MRI images collected from 40 unique patients at the National Institute of Neurosciences and Hospital, Dhaka, Bangladesh.

The original MRI data were collected in DICOM format and subsequently converted to PNG format for use in our experiments. Suitable MRI slices were selected based on the recommendation of Dr. Md. Ashraful Haque, a neurosurgeon at the same hospital. He reviewed the selected PNG images and assigned the corresponding class labels. The dataset was collected and used with institutional approval.

The class-wise distribution of the TEST2 dataset is presented in Table 4.

### 3.2. Pretrained Backbone Selection

Modern convolutional and transformer-style backbones were mixed to capture complementary features while keeping features compact and fast to extract [34,35,36,37,38]. Importantly, the backbone choice was rationale-driven rather than random: The backbones were intentionally selected to cover major computer-vision paradigms and provide diverse feature representations. Specifically, the six pretrained backbones represent global attention (ViT), hierarchical window attention (Swin), modern convolution (ConvNeXt), efficient CNN scaling (EfficientNetV2), dense connectivity (DenseNet), and self-supervised semantic learning (DINOv3).

CNNs such as ConvNeXt-B, EfficientNetV2-RW-S, and DenseNet-201 provide hierarchical, locality- and texture-sensitive representations learned via convolution and pooling layers [34,35,36]. Swin-S contributes windowed self-attention with shifted windows, balancing locality with longer-range context [37]. Two ViT-B/16 variants (one supervised on ImageNet-1k and one self-supervised in the DINO family) add global attention features [38,39,40]. This architectural diversity tends to reduce errors and improve robustness when the models are fused or stacked. At the same time, the pooled descriptor sizes (768–1920 dimensions depending on the backbone) remain small enough for lightweight fusion heads [34,35,36,37,38]. We selected only backbones that are widely adopted, actively maintained, and accessible through standardized libraries such as timm, ensuring consistent and well-documented preprocessing (e.g., input resizing and normalization) [41]. All backbones are used strictly as feature extractors: pretrained weights are loaded, but no fine-tuning was performed.

### 3.3. Feature Extraction and Standardization

Each image *x* is represented using features from *S* pretrained backbones and a lightweight modality fingerprint (DIP). These features are extracted once and saved. Then, only the fusion head is trained on the saved features, while keeping all backbone weights frozen.

#### 3.3.1. Backbone Descriptors

For each backbone b∈{1,…,S} with last-layer representation $\phi_{b}\left(x\right)$, a single pooled vector is computed:

(1) $$f_{b}\left(x\right)\in R^{d_{b}}\;\mathrm{with}\;f_{b}\left(x\right)=\left\{\begin{array}{cc} \mathrm{GAP}\;\left(\phi_{b}\left(x\right)\right), & \mathrm{CNNs},\\ \frac{1}{T}\sum_{t=1}^{T}\phi_{b}^{\left(t\right)}\left(x\right), & \mathrm{ViTs}. \end{array}\right.$$

For CNNs, global average pooling (GAP) is applied, and for ViTs, the mean of the final-layer tokens is used. When test-time augmentation (TTA) is enabled, further averaging across the *T* augmented views is performed. The per-backbone feature vectors are then concatenated:

(2) $$F\left(x\right)\;=\;\left[f_{1}\left(x\right);\ldots ;f_{S}\left(x\right)\right]\in R^{D},\;D=\sum_{b=1}^{S}d_{b}.$$

#### 3.3.2. DIP Fingerprint Features

Along with deep features from the backbones, handcrafted features are added that summarize global appearance and intensity structure of each image. This DIP fingerprint is cheap to compute and helps the model by giving additional hints about modality/contrast, exposure, tissue–background balance, and basic texture that are often complementary to high-level deep features.

Preprocessing. The input image is converted to an 8-bit grayscale image of size H×W, with pixel values in {0,…,255}. The intensities are normalized by $\tilde{x}=x/255$, so that $\tilde{x}\in {\left[0,1\right]}^{H\times W}$.

Components of the fingerprint. The descriptor concatenates a normalized intensity histogram and six scalar statistics, as summarized in Table 5.

**Figure 3:**
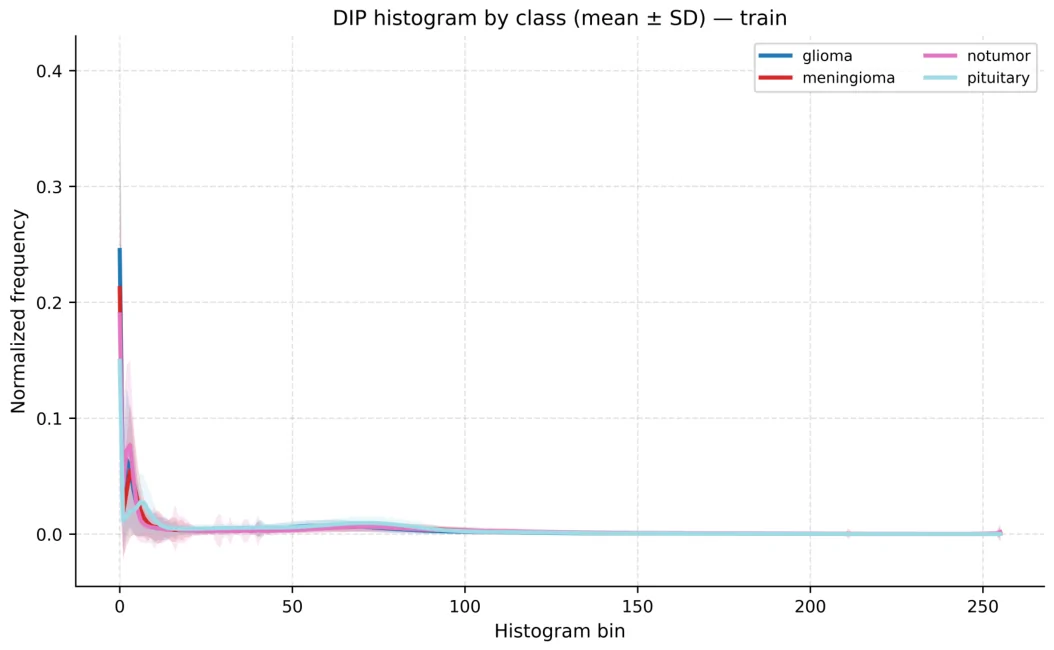
Class-wise DIP histogram on the training split.

The class-wise DIP histograms are shown in Figure 3. They show a sharp peak at the lowest bin for all classes, indicating a large proportion of near-zero pixels (mostly background or black borders in MRI images). After the first few bins, the distributions drop quickly and largely overlap, suggesting that the overall intensity profiles are similar across classes. A small bump is visible around bins ≈ 60–90, where the pituitary curve is slightly higher than the others, indicating a modest shift toward mid-range intensities. The mean ± SD bands overlap substantially, although the no-tumor class shows slightly higher variability in some low-intensity regions. Overall, the DIP histogram mainly captures global brightness/contrast characteristics and can provide weak but complementary cues.

**Figure 4:**
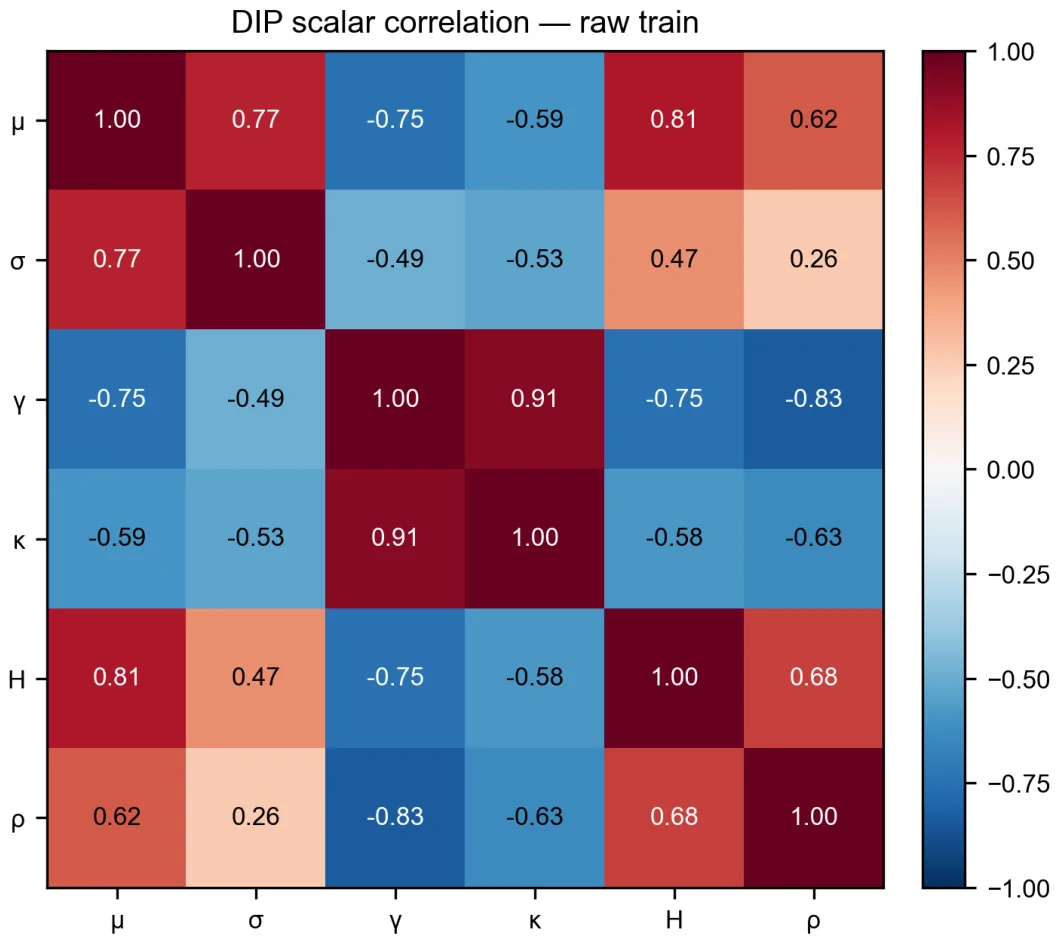
Correlation map among scalar DIP features on the training split.

The scalar correlation map among DIP statistics is shown in Figure 4. It shows clear relationships among the DIP statistics. Mean intensity (μ) is strongly positively correlated with standard deviation (σ, 0.77) and entropy (*H*, 0.77), so brighter images tend to have more intensity variation and more complex distributions. Entropy is also positively correlated with the Otsu foreground ratio (ρ, 0.70), meaning images with more foreground area usually have richer intensity structure. Skewness (γ) and kurtosis (κ) are strongly correlated (0.91), since both describe the shape of the intensity distribution. In contrast, μ is negatively correlated with γ (−0.74) and κ (−0.59), and γ is strongly negative with ρ (−0.83), suggesting that images with more foreground and higher brightness are less skewed. These trends show that the scalar features capture complementary information rather than duplicating each other.

**Figure 5:**
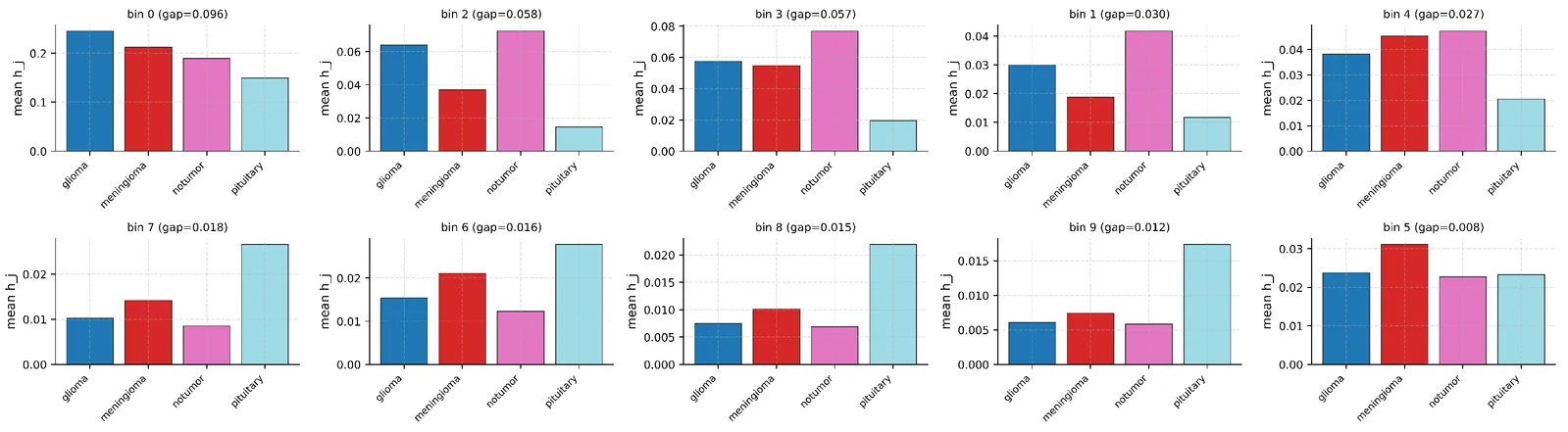
Most discriminative DIP histogram bins on the training split.

The most discriminative DIP histogram bins are presented in Figure 5. Bar charts of the most discriminative histogram bins (top bins by mean gap) show that the strongest class differences appear in the earliest bins (bins 0–4), which represent very dark pixels. Glioma has the highest value in bin 0, while pituitary is the lowest. Notumor is relatively higher in bins 1–4. In contrast, pituitary becomes highest in bins 6–9, indicating more low-to-mid-intensity content and less pure-black background. These consistent shifts show that a few low-intensity bins already carry useful modality/contrast cues.

**Figure 6:**
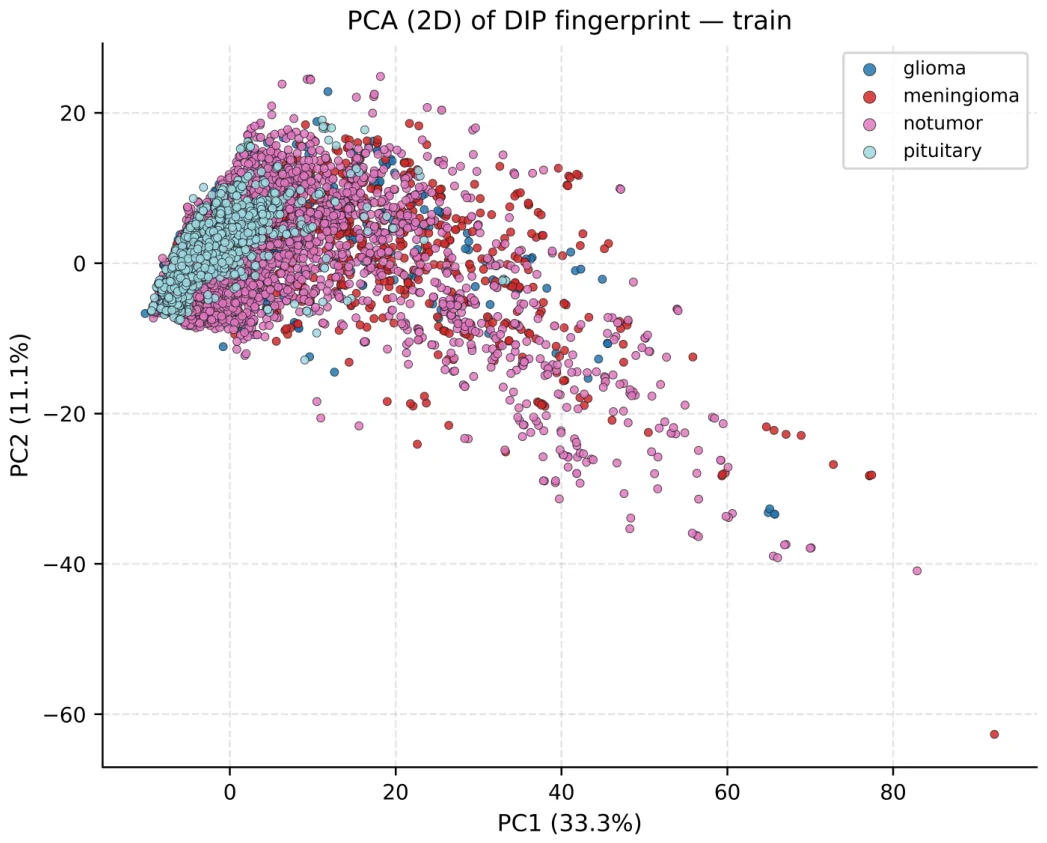
PCA projection of DIP fingerprints on the training split.

The PCA projection of the DIP fingerprints is shown in Figure 6. PCA (2D) of the DIP fingerprints shows partial structure but also strong overlap between classes. Pituitary samples form a tighter cluster toward the left side, while notumor spreads widely and overlaps with tumor classes. Meningioma extends more along PC1 and includes a few outliers, and glioma overlaps with both meningioma and notumor in the middle region. Since PC1 explains 33.3% and PC2 explains 11.1% of the variance, DIP alone cannot perfectly separate classes, but it provides complementary global intensity information that can support the deep features.

**Figure 7:**
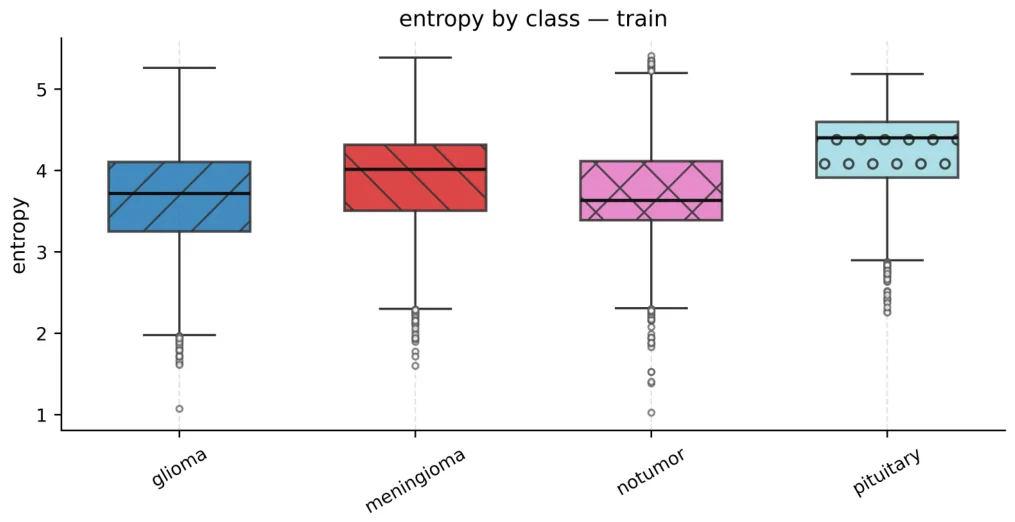
Entropy by class.

The entropy distributions across classes are shown in Figure 7. Pituitary shows the highest median entropy, while glioma and notumor are lower (notumor slightly lowest), suggesting pituitary scans contain richer intensity mixtures.

The following boxplots use the same convention: the box represents the interquartile range (IQR), the horizontal line indicates the median, and circles represent outliers.

**Figure 8:**
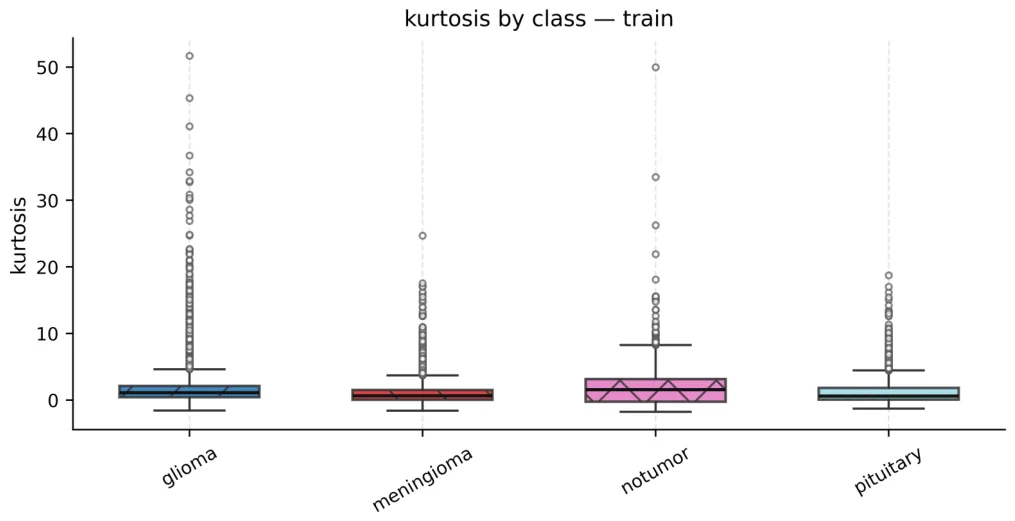
Kurtosis by class.

The kurtosis distributions across classes are shown in Figure 8. Notumor has the highest median and widest IQR, indicating more frequent heavy-tailed/peaky histograms. Glioma also exhibits very large kurtosis outliers, suggesting some highly peaked cases.

**Figure 9:**
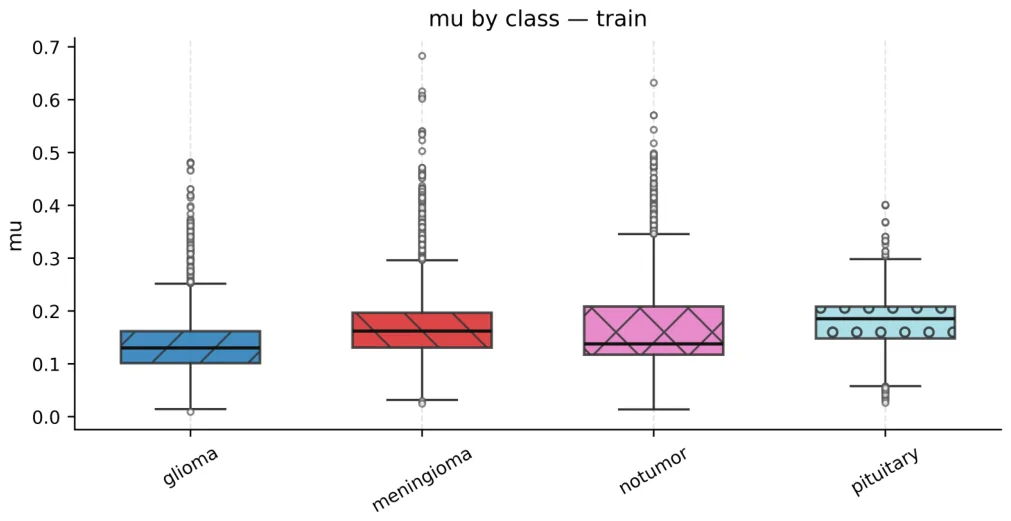
Mean intensity (μ) by class.

The mean intensity distributions across classes are shown in Figure 9. Glioma is darkest on average (lowest median μ), pituitary is brightest, and meningioma is generally brighter than notumor.

**Figure 10:**
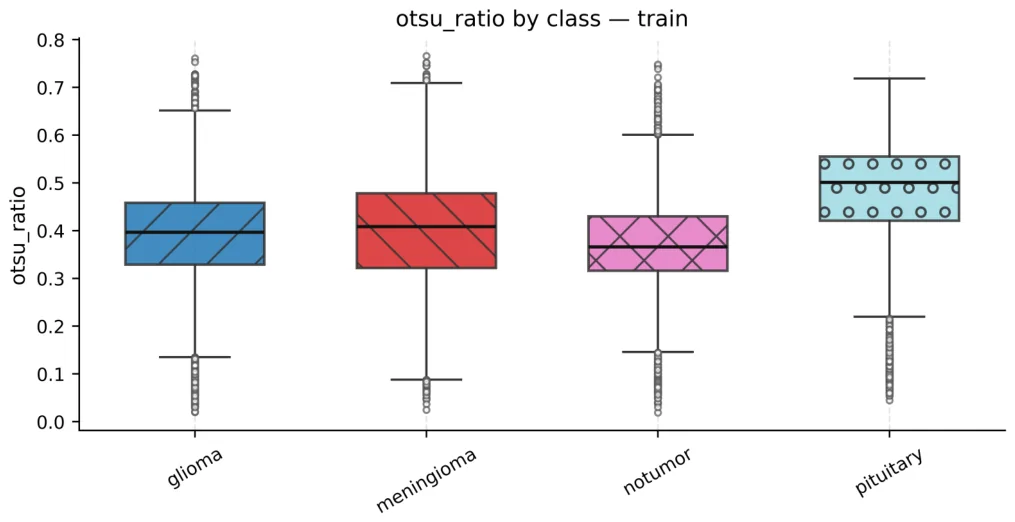
Otsu foreground ratio (ρ) by class.

The Otsu foreground ratio distributions across classes are shown in Figure 10. Pituitary has the highest median (∼0.5), glioma/meningioma are intermediate (∼0.40), and notumor is lower (∼0.35–0.37), indicating smaller foreground regions under Otsu thresholding.

**Figure 11:**
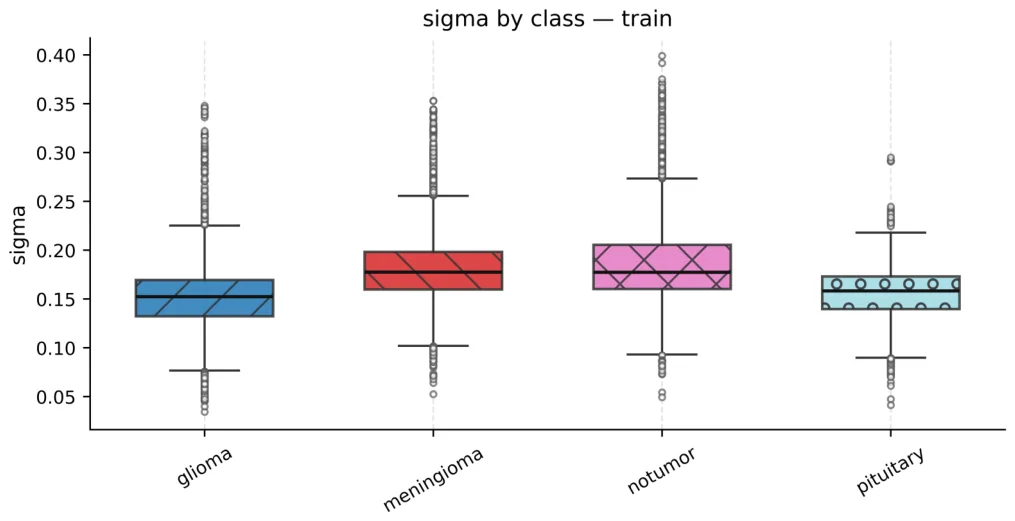
Standard deviation (σ) by class.

The standard deviation distributions across classes are shown in Figure 11. Notumor shows the highest variability and broad dispersion, while glioma shows the lowest variability, indicating stronger heterogeneity in grayscale content for notumor images.

**Figure 12:**
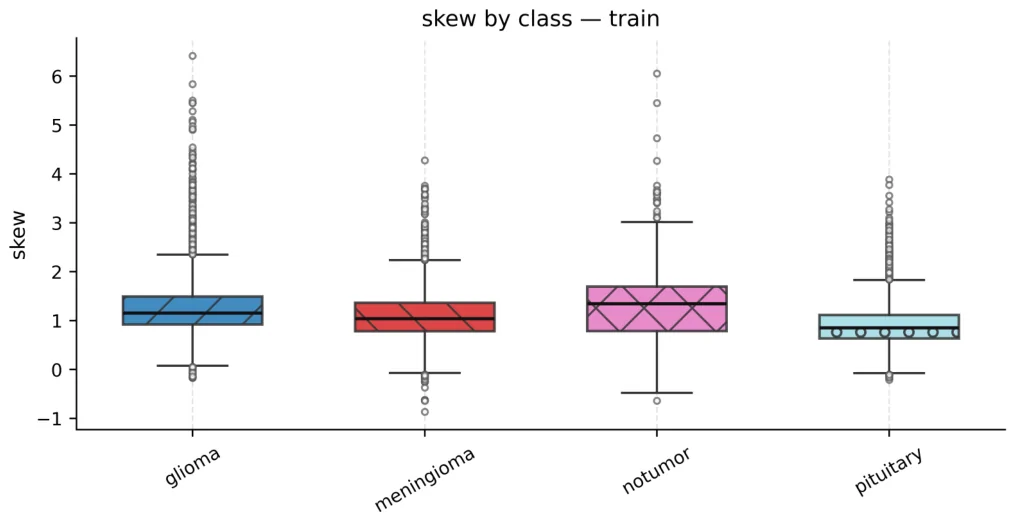
Skewness (γ) by class.

The skewness distributions across classes are shown in Figure 12. All classes are positively skewed (many dark pixels), with notumor showing the highest median skewness and pituitary the lowest; glioma also has a long positive tail with high-skew outliers.

#### 3.3.3. Z-Score Standardization

Different backbones (CNNs and ViTs) and the optional modality fingerprint can produce features on different numeric scales. If left uncorrected, large-magnitude features may dominate training. To place all signals on a common scale, each feature dimension is standardized using statistics from the training split only (to avoid leakage). For any vector $z\in R^{d}$, let μ and σ denote the per-dimension mean and standard deviation computed on the training split. Then, the following is applied:

$$\tilde{z}\;=\;\frac{z-\mu }{\max(\sigma ,\;10^{-6})}.$$

where the per-dimension standard deviation is lower-bounded at $10^{-6}$ for numerical stability.

(μ,σ) is estimated once on the concatenated backbone descriptor F using the training split only, and the same parameters are reused for validation and test (no recomputation, no leakage). If the modality fingerprint r is enabled, it is standardized separately with its own training-only (μ,σ) to ensure numerical stability. The standard deviation is not allowed to become too small. Therefore, a lower bound of $10^{-6}$ is set.

### 3.4. Classification Heads

All classifiers were small, MLP-based heads trained on cached, standardized features.

#### 3.4.1. Concatenation MLP (Concat)

This head simply stacked all feature vectors into one input. If the modality fingerprint was enabled, it was appended to the backbone features. Let $\tilde{F}\in R^{D}$ be the standardized concatenation of all backbones and let $\tilde{r}\in R^{70}$ denote the (optional) standardized fingerprint. The input to the MLP was

$$x=\left\{\begin{array}{cc} {}[\tilde{F},\tilde{r}], & \mathrm{if}\;\mathrm{the}\;\mathrm{fingerprint}\;\mathrm{was}\;\mathrm{used},\\ \tilde{F} & \mathrm{otherwise}. \end{array}\right.$$

The classifier consisted of a two-layer MLP with hidden dimensions of 1024 and 256 units, using dropout rates of 0.30 and 0.20, respectively, and an activation function *g* (Mish [45], SiLU [46], or GELU [47]):

$$\hat{y}=\mathrm{softmax}\;\left(W_{3}\;g\left(W_{2}\;g\left(W_{1}\;x\right)\right)\right)\in \Delta^{K-1}.$$

This architecture was effective because providing the MLP with the complete concatenated feature vector allowed it to learn relationships between the outputs of different backbones directly within its hidden layers. Despite its simplicity, this baseline model demonstrated strong performance and computational efficiency.

#### 3.4.2. Attention Fusion (Attn)

In this approach, the backbone feature streams were explicitly fused using a lightweight, stream-level attention mechanism inspired by channel attention [48]. Each standardized backbone feature ${\tilde{f}}_{j}\in R^{d_{j}}$ was first projected into a shared latent space of dimension $d_{f}=1024$:

$$z_{j}=P_{j}\;{\tilde{f}}_{j}\in R^{d_{f}},\;j=1,\ldots ,S.$$

A compact two-layer gating network (using the GELU activation [47]) then produced one scalar score per stream, which was normalized with a softmax function to obtain importance weights $a_{j}$. These weights were used to compute a weighted fusion of the projected stream features:

$$s_{j}=v^{⊤}\;\mathrm{GELU}\left(W_{g}\;z_{j}\right),\;a_{j}=\frac{e^{s_{j}}}{\sum_{\ell =1}^{S}e^{s_{\ell }}},\;z_{⋆}=\sum_{j=1}^{S}a_{j}\;z_{j}.$$

More informative backbones received higher weights for each input sample. The fused representation $z_{⋆}$ (optionally concatenated with the projected fingerprint feature) was then passed through a two-layer MLP classifier to generate the final class logits via

$$\hat{y}=\mathrm{softmax}\left(W_{3}\;g\left(W_{2}\;g\left(W_{1}\;\left[z_{⋆},P_{\mathrm{fp}}\tilde{r}\right]\right)\right)\right).$$

Conceptually, this attention-based head learned a soft, sample-specific weighting across the feature streams, enabling the model to prioritize more informative backbones for each input, while remaining computationally efficient.

#### 3.4.3. Gated Fusion (Gated)

In this head, the information from multiple feature streams was combined using a simple gating mechanism, similar in principle to mixture-of-experts gating [49]. Each gate learned how much attention to give to each backbone’s features for a given input. First, all standardized feature streams were concatenated into a single vector:

$$x=[{\tilde{f}}_{1},{\tilde{f}}_{2},\ldots ,{\tilde{f}}_{S}],$$

and if the fingerprint feature was available, it was added as an extra stream at the end.

A small linear layer followed by a sigmoid activation generated one gate value for each stream:

$$s=\mathrm{sigmoid}\left(W_{\mathrm{gate}}\;x\right)\in {\left(0,1\right)}^{S}.$$

Each gate $s_{j}$ acted like a weight, controlling how much of its corresponding stream ${\tilde{f}}_{j}$ should contribute to the final prediction. The streams were then multiplied by their gate values and concatenated again:

$$x_{g}=\left[\;s_{1}{\tilde{f}}_{1},\;s_{2}{\tilde{f}}_{2},\;\ldots ,\;s_{S}{\tilde{f}}_{S}\;\right].$$

Before classification, Layer Normalization [50] was applied to stabilize the feature magnitudes across streams:

$$x_{g}=\mathrm{LN}\left(x_{g}\right).$$

The normalized features were then passed through a lightweight MLP with one hidden layer (2048 units, GELU activation [47] and 0.10 dropout [12]) to produce the class logits, which were finally converted to probabilities using softmax.

Layer Normalization helped balance the contribution of different backbones, especially since CNN and ViT features can have different scales. Although the gating nonlinearity was fixed to GELU, three models were trained with different random seeds and their outputs averaged using validation-based weights, in line with the other heads.

### 3.5. Training and Regularization

Training was kept simple and consistent across heads with cross-entropy with label smoothing, exponential moving average (EMA) of weights, and mixup in feature space applied to 50% of mini-batches. Label smoothing was used as a mild regularizer [11], EMA with decay 0.999 to stabilize training [10], and mixup to reduce overfitting and label-noise sensitivity [9]. At test time, logits were calibrated by temperature scaling fitted on the validation set, following best practices for medical-image classifiers [7,51]. Specifically, ε=0.10 was used for label smoothing, mixup parameter α=0.4, EMA decay τ=0.999, warmup fraction φ=0.05, a base learning rate of $\eta_{0}=2\times 10^{-4}$, and a minimum learning rate $\eta_{\min}=10^{-6}$. Models were optimized with AdamW (weight decay $5\times 10^{-5}$) and checkpoints were selected based on the best validation accuracy. After training, a scalar temperature was fitted per head for post-hoc calibration on validation logits. For the super-ensemble, a new temperature was refitted while the stacking stage used the already calibrated base probabilities.

#### 3.5.1. Label-Smoothed Cross-Entropy

Let ${\hat{y}}_{i}=\mathrm{softmax}\left(z_{i}\right)$ denote the predicted-class probabilities for sample *i* and $t_{i}$ the corresponding one-hot label over *K* classes. With smoothing parameter ε [11], the per-sample loss becomes

(3) $$\ell_{i}=-\sum_{k=1}^{K}\left(\left(1-\varepsilon \right)\;t_{ik}+\frac{\varepsilon }{K}\right)\log{\hat{y}}_{ik}.$$

Label smoothing prevents the model from becoming overconfident, helping it generalize better and produce more calibrated predictions.

#### 3.5.2. MixUp in Feature Space

Since the backbone networks remain frozen and features are precomputed, mixup is applied directly in feature space (including the optional fingerprint features). For each minibatch, λ∼Beta(α,α) is sampled, and the mixed inputs and labels are formed:

(4) $$\tilde{x}=\lambda \;x+\left(1-\lambda \right)\;x^{\prime },\;\tilde{t}=\lambda \;t+\left(1-\lambda \right)\;t^{\prime }.$$

The training loss under mixup is a weighted combination of the two corresponding cross-entropies:

(5) $$\ell_{\mathrm{mix}}=\lambda \;\mathrm{CE}\left(z,t\right)+\left(1-\lambda \right)\;\mathrm{CE}\left(z,t^{\prime }\right).$$

Applying mixup at the feature level serves as both data augmentation and regularization, producing smoother decision boundaries and greater robustness to noise [9].

#### 3.5.3. Exponential Moving Average (EMA) of Weights

To improve stability and generalization, an exponential moving average of model parameters was maintained throughout training [10]. Denoting the current parameters by $\theta_{t}$, the EMA weights ${\bar{\theta }}_{t}$ are updated as

(6) $${\bar{\theta }}_{t}=\tau \;{\bar{\theta }}_{t-1}+\left(1-\tau \right)\;\theta_{t}.$$

All validation metrics and model selection were computed using the EMA-averaged weights.

#### 3.5.4. Warmup–Cosine Learning-Rate Schedule

Linear warmup is used for the first 5% of training steps (about 2 epochs for 40 total), followed by cosine decay to $\eta_{\min}=10^{-6}$. The learning rate is updated every optimization step. Cosine annealing [52] with linear warmup is followed.

#### 3.5.5. Post-Hoc Temperature Scaling

To improve calibration, temperature scaling was applied on the validation logits. Given logits z, a scalar temperature T>0 is sought that minimizes the negative log-likelihood (NLL) of the softmax outputs:

(7) $$p_{k}\left(T\right)=\frac{\exp(z_{k}/T)}{\sum_{j}\exp\left(z_{j}/T\right)},\;T^{⋆}=\arg\min_{T}\mathrm{NLL}\left(T\right).$$

In practice, a grid search over T∈[0.3,3.0] was performed using 55 evenly spaced candidates, selecting the value that minimized NLL on the validation set. The chosen temperature $T^{⋆}$ was then applied to both validation and test logits to obtain calibrated probabilities. This procedure adjusts the confidence scale without changing prediction accuracy, following best practices for neural network calibration [7,51].

#### 3.5.6. Early Stopping, Reproducibility, and Hyperparameter Selection

Training was run for up to 40 epochs, with early stopping based on validation accuracy using a patience value of 8. To ensure reproducibility, all random seeds for data shuffling, mixup sampling, and parameter initialization were fixed. Standardization statistics were computed using only the training set to prevent information leakage.

The selection of the final training configuration was not arbitrary. Several classifier types, activation functions, optimizers, losses, and training hyperparameters were examined during the development process. Some configurations were explored through grid search, while the final settings were selected based on validation performance and error analysis. The candidate choices and final selected settings are summarized in Table 6.

### 3.6. Ensembling

**Figure 13:**
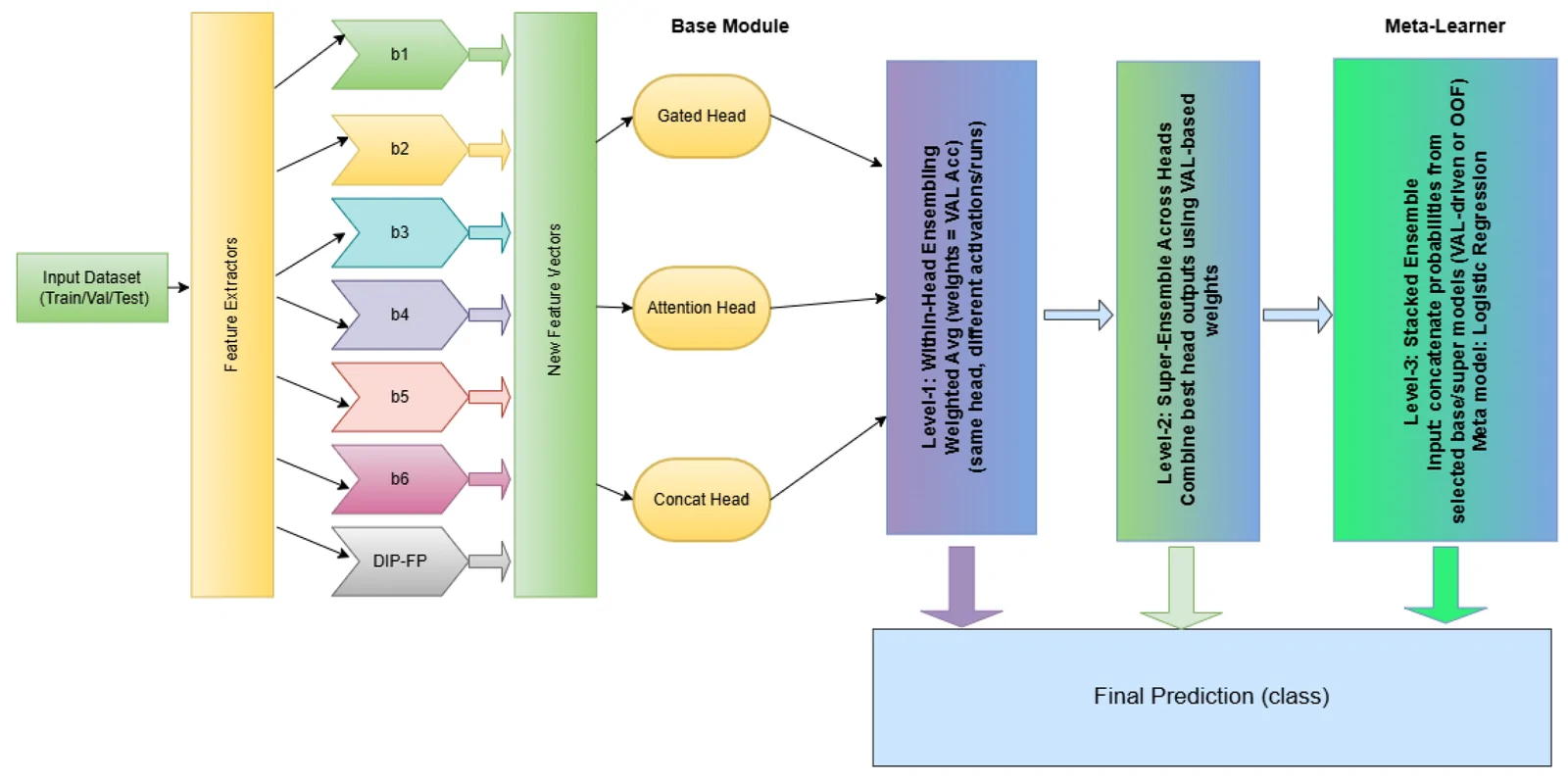
Proposed three-level ensembling strategy: (Level 1) within-head activation ensembling, (Level 2) super-ensemble across fusion heads, and (Level 3) stacked ensemble with an $\ell_{2}$-regularized logistic-regression meta-learner.

To maximize predictive performance and robustness, models are ensembled at three distinct levels: (i) within-head activation ensembling, which combines multiple training runs of the same fusion head using different nonlinearities, (ii) super-ensemble across heads, which aggregates the best-performing variant from each fusion architecture, and (iii) stacked ensemble via meta-learning, which trains a second-stage classifier on the probability outputs of base models. Each level targets a different source of model diversity and provides complementary benefits. The proposed ensembling strategy is shown in Figure 13.

#### 3.6.1. Within-Head Activation Ensembling

For each fusion head h∈{concat,attn,gated}, three variants are trained using A={Mish,SiLU,GELU} in the hidden layers. All other settings (splits, optimizer, schedule, regularization) match Section 3.5. Distinct random seeds further promote diversity.

#### 3.6.2. Validation-Weighted Logit Averaging

After training the three activation variants, each is evaluated on the validation set using EMA-averaged weights. Let $z_{h,a}^{\mathrm{val}}\left(x_{i}\right)$ be the logits produced by model (h,a) on validation sample $x_{i}$ with ground-truth $y_{i}$. Define its validation accuracy

(8) $$\alpha_{h,a}=\frac{1}{N_{\mathrm{val}}}\sum_{i=1}^{N_{\mathrm{val}}}⊮\;\left[\;\arg\max_{k}\mathrm{softmax}\;{\left(z_{h,a}^{\mathrm{val}}\left(x_{i}\right)\right)}_{k}\;=\;y_{i}\;\right].$$

Per-model weights are derived via min–max normalization mapped to [0.5,1.0]:

(9) $$w_{h,a}=0.5+0.5\times \frac{\alpha_{h,a}-\min_{a^{\prime }}\alpha_{h,a^{\prime }}}{\max_{a^{\prime }}\alpha_{h,a^{\prime }}-\min_{a^{\prime }}\alpha_{h,a^{\prime }}+\varepsilon },\;\varepsilon =10^{-6}.$$

At inference time (validation or test), ensembling is performed by logit averaging with these weights:

(10) $${\tilde{z}}_{h}\left(x\right)=\frac{\sum_{a\in A}w_{h,a}\;z_{h,a}\left(x\right)}{\sum_{a\in A}w_{h,a}},$$

Then, ${\tilde{z}}_{h}\left(x\right)$ is calibrated using temperature scaling.

#### 3.6.3. Super-Ensemble Across Heads

The super-ensemble aggregates the best-performing variant from each fusion head type, adding architectural diversity among concat, attn, and gated fusion. This approach extends the within-head ensembling scheme described earlier.

#### 3.6.4. Best-Variant Selection

For each head h∈{concat,attn,gated}, the activation variant with the highest validation accuracy is selected:

(11) $$a_{h}^{⋆}=\arg\max_{a\in A}\alpha_{h,a}.$$

The corresponding logits $z_{h}^{⋆}\left(x\right)$ (using EMA weights) represent the best configuration for that head. The selected heads are then combined using the same validation-weighted logit averaging and temperature-scaling strategy introduced in Section 3.6.1.

#### 3.6.5. Stacked Ensemble via Meta-Learning

Stacking is a powerful ensemble technique in which a meta-learner is trained to optimally combine predictions from multiple base models [53,54].

#### 3.6.6. Base Models and Meta-Features

Stacked ensembles are constructed using subsets of the fusion heads. Let $H_{\mathrm{stack}}\subseteq \left\{\mathrm{concat},\mathrm{attn},\mathrm{gated}\right\}$ denote the set of base heads included in the stack. For each head $h\in H_{\mathrm{stack}}$, the calibrated probabilities from the within-head ensemble are used as base predictions. Specifically, for a sample $x_{i}$, the meta-feature vector is the concatenation of all base probabilities:

(12) $$\phi \left(x_{i}\right)=\left[\;{\hat{p}}_{h_{1}}\left(x_{i}\right),\;{\hat{p}}_{h_{2}}\left(x_{i}\right),\;\ldots ,\;{\hat{p}}_{h_{m}}\left(x_{i}\right)\;\right]\in R^{m\times K},$$

where $m=|H_{\mathrm{stack}}|$ is the number of base heads and K=4 is the number of classes.

#### 3.6.7. Meta-Learner Architecture and Training

A multinomial logistic regression (softmax regression) is used as the meta-learner, trained with $\ell_{2}$ regularization:

(13) $${\hat{y}}_{\mathrm{stack}}\left(x\right)=\mathrm{softmax}\left(W\phi (x)+b\right),$$

where $W\in R^{K\times (m\;\cdot \;K)}$ and $b\in R^{K}$ are learned parameters. The loss function is the regularized cross-entropy:

(14) $$L_{\mathrm{stack}}=-\frac{1}{N}\sum_{i=1}^{N}\sum_{k=1}^{K}y_{ik}\log{\hat{y}}_{\mathrm{stack},ik}+\frac{\lambda }{2}{∥W∥}_{F}^{2},$$

where λ controls the strength of $\ell_{2}$ regularization (we use λ=1.0, equivalent to C=1.0 in scikit-learn’s LogisticRegression).

#### 3.6.8. Training Variants: VAL-Driven vs. TRAIN-OOF Stacking

Two complementary protocols for training the stacking meta-learner were implemented. Both approaches use predictions from several base heads (concat, attn, gated), but differ in how the input meta-features are obtained, either from validation predictions or from out-of-fold (OOF) training predictions.

(i) VAL-driven Stacking (Standard Approach). In this approach, the stacking model learns directly from the validation predictions of each head. For every base head *h*, its class probabilities are first calibrated on the validation set $D_{\mathrm{val}}$ by learning a temperature-scaling factor $T_{h}$ and applying:

$${\hat{p}}_{h}\left(x\right)=\mathrm{softmax}\left(z_{h}\left(x\right)/T_{h}\right),$$

where $z_{h}\left(x\right)$ are the logits of head *h*.

The calibrated probabilities from all heads are concatenated to form the meta-feature vector for each sample:

$$\phi \left(x\right)=[{\hat{p}}_{h_{1}}\left(x\right)\;∥\;{\hat{p}}_{h_{2}}\left(x\right)\;∥\;\cdots \;∥\;{\hat{p}}_{h_{\left|H\right|}}\left(x\right)].$$

To avoid overfitting, the meta-learner (a multinomial logistic regression) is trained using 5-fold cross-validation within the validation set. In each fold, the model is trained on four parts and tested on the remaining one to produce out-of-fold (OOF) predictions, which are later merged to compute an unbiased VAL metric. After the loop, every VAL sample has an out-of-fold prediction. Then, Accuracy, F1, AUROC, ECE, and Brier are computed using the OOF predictions. Finally, the meta-learner is retrained on the full validation features and applied to the test set for final evaluation.

This protocol is fast and practical, but since the validation set is used both for model selection and meta-learning, its results may be slightly optimistic.

(ii) TRAIN-OOF Stacking (Rigorous Approach). The TRAIN-OOF method provides a more robust and unbiased evaluation using out-of-fold predictions from the train set to build the meta-features.

The training split $D_{\mathrm{train}}$ is partitioned into five stratified folds $\left\{F_{1},\ldots ,F_{5}\right\}$.

For each fold *k*:

- Each head is trained on the remaining four folds (the “fold-train” split).
- 10% of those fold-train data are reserved as an inner-validation set for picking the best variant of each head (by activation), early stopping, and temperature calibration.
- The trained model then predicts on the held-out fold $F_{k}$, producing calibrated OOF probabilities for that fold.

After all five folds are processed, every training sample has exactly one unbiased OOF prediction, i.e., a prediction made by a model that never saw that sample during training. These predictions are then used to train the logistic-regression meta-learner.

This approach prevents direct leakage between base-model training and meta-learning for each OOF sample and therefore provides a more rigorous stacking protocol.

#### 3.6.9. Within-Head Prediction Sources (ens = True vs. ens = False)

Each head can contribute its predictions to the stacking framework in one of two ways:

- Single best activation (ens = False): Each head is trained with multiple activation functions (Mish, SiLU, GELU). Only the best-performing variant on the validation set is kept in this case.
- Within-head activation ensemble (ens = True): Predictions from all activation variants are averaged (weighted by their validation accuracies) before temperature calibration. This produces smoother, more reliable head outputs.

#### 3.6.10. Meta-Feature Standardization

Before training the meta-learner, we scale the stacked probability features $\phi \left(x\right)\in {\left[0,1\right]}^{H\;\cdot \;C}$ so that all feature dimensions are on a similar scale. For each feature *j*, we divide by its standard deviation $\sigma_{j}$ computed from the meta-training set:

$${\tilde{\phi }}_{j}\left(x\right)=\frac{\phi_{j}\left(x\right)}{\sigma_{j}}.$$

This is implemented with StandardScaler with mean = False, i.e., we scale each column but do not subtract the mean. As a result, the features stay non-negative while reducing the chance that one head or class probability dominates the meta-learner due to having a larger numeric scale. Although the scaled vector is no longer a probability distribution, it works well as input to the logistic-regression meta-learner.

While the VAL-driven stacking protocol offers a fast and effective baseline, the TRAIN-OOF approach provides an unbiased and rigorous alternative.

#### 3.6.11. Test-Time Inference and Abstention

At inference, all ensemble configurations (within-head, super-ensemble, and stacking) follow a unified protocol.

#### 3.6.12. Test-Time Augmentation (TTA)

At inference, for each sample *x*, global features are extracted from all frozen backbone networks. For evaluation splits (validation/test and external TEST2), we apply flip-based TTA by horizontally flipping the input and averaging the resulting feature vectors from the original and flipped views. If the DIP fingerprint is enabled, it is computed for each image and then z-score standardized using statistics estimated from the training set only.

#### 3.6.13. Detection (Tumor vs. No-Tumor) from Multiclass Outputs

Although the models are trained for *K*-class tumor subtype classification, we additionally evaluate a binary detection setting (tumor present vs. no-tumor) using the same temperature-calibrated class probabilities. Let $\hat{p}\left(x\right)=\mathrm{softmax}\left(z\left(x\right)/T\right)$ denote the calibrated probability vector, and let $k_{\mathrm{none}}$ be the index of the no-tumor class. We define the tumor score as

$$p_{\mathrm{tumor}}\left(x\right)=1-{\hat{p}}_{k_{\mathrm{none}}}\left(x\right).$$

A sample is predicted as tumor if $p_{\mathrm{tumor}}\left(x\right)\geq \tau_{\det}$ with $\tau_{\det}=0.50$; otherwise, it is predicted as no-tumor.

## 4. Results and Analysis

### 4.1. Evaluation Metrics

This study evaluates model performance using the following metrics.

Accuracy and confidence intervals. Accuracy (acc) is computed as the proportion of correctly classified samples. On TEST, a 95% percentile bootstrap confidence interval is reported using $B_{\mathrm{boot}}=1000$ nonparametric resamples [55].

Macro-F1. Macro-averaged F1 (F1) is computed by taking the unweighted mean of the per-class F1 scores, so that each class contributes equally regardless of class frequency [56].

Brier score. Probabilistic accuracy is assessed using the multiclass Brier score (Brier) [57,58], defined as the mean squared difference between the predicted probability vector and the one-hot encoded ground-truth label. Lower values indicate more accurate probability estimates.

Expected Calibration Error (ECE). Calibration is evaluated using the top-label Expected Calibration Error (ECE) [59]. Predictions are partitioned into M=10 equal-width bins according to confidence (the maximum predicted probability). ECE is computed as the weighted average across bins of the absolute difference between the bin-wise accuracy and the bin-wise confidence.

### 4.2. Implementation and Reproducibility

All experiments are conducted using an NVIDIA RTX 5090 GPU. A fixed random seed and deterministic cuDNN operations are used to ensure reproducibility.

### 4.3. Performance Evaluation

The performance of all evaluated variants is summarized in Table 7. The following analysis is based on the results reported in this table.

#### 4.3.1. Effect of FP (Distributional Fingerprint) Features

The effect of FP is head-dependent. For the concat head, FP is helpful on VAL but not on TEST or TEST2. For the attention and gated heads, FP improves TEST performance and gives gains on TEST2.

Concat head (ensemble_FP vs. ensemble_no_FP): VAL is slightly higher with FP: 0.9929 vs. 0.9926 (+0.03 pp). However, TEST is slightly lower: 0.9923 vs. 0.9928 (−0.05 pp), and TEST2 is also lower: 0.8783 vs. 0.8827 (−0.44 pp).
Attention head (ensemble_FP vs. ensemble_no_FP): FP improves the attention head consistently in this comparison: VAL 0.9920 vs. 0.9897 (+0.23 pp), TEST 0.9899 vs. 0.9875 (+0.24 pp), and TEST2 0.9516 vs. 0.9194 (+3.22 pp).
Gated head (ensemble_FP vs. ensemble_no_FP): For the gated head, FP slightly lowers accuracy on VAL: 0.9923 vs. 0.9929 (−0.06 pp), but higher on TEST: 0.9933 vs. 0.9913 (+0.20 pp), and also higher on TEST2: 0.9047 vs. 0.8856 (+1.91 pp).

Overall, FP is most helpful for the attention and gated heads, especially on local hospital data TEST2. However, it is not beneficial for concat on TEST2.

#### 4.3.2. Effect of Within-Head Activation Ensembles

The effect of within-head activation ensembling using Mish, SiLU, and GELU also depends on the head type. Consistent gains are observed for the attention head, especially on TEST2. For the gated head, ensembling is usually helpful on TEST or TEST2. For the concat head, the effect is small and mixed.

Concat head (ensemble vs. single): FP: ensemble is slightly lower than single VAL (0.9929 vs. 0.9936, −0.07 pp) and TEST (0.9923 vs. 0.9928, −0.05 pp), but slightly higher on TEST2 (0.8783 vs. 0.8768, +0.15 pp).
  no-FP: ensemble and single are identical here (VAL 0.9926 vs. 0.9926; TEST 0.9928 vs. 0.9928), but ensemble is lower on TEST2 (0.8827 vs. 0.8886, −0.59 pp).
Attention head (ensemble vs. single): FP: ensemble improves over single on all three splits: VAL 0.9920 vs. 0.9894 (+0.26 pp), TEST 0.9899 vs. 0.9889 (+0.10 pp), and TEST2 0.9516 vs. 0.9267 (+2.49 pp).
  no-FP: ensemble is slightly higher on VAL (0.9897 vs. 0.9894, +0.03 pp), slightly lower on TEST (0.9875 vs. 0.9880, −0.05 pp), but better on TEST2 (0.9194 vs. 0.8988, +2.06 pp).
Gated head (ensemble vs. single): FP: ensemble is slightly lower on VAL (0.9923 vs. 0.9933, −0.10 pp), but higher on TEST (0.9933 vs. 0.9918, +0.15 pp), and much higher on TEST2 (0.9047 vs. 0.8768, +2.79 pp).
  no-FP: ensemble is slightly lower on VAL (0.9929 vs. 0.9939, −0.10 pp), slightly higher on TEST (0.9913 vs. 0.9909, +0.04 pp), and only marginally higher on TEST2 (0.8856 vs. 0.8842, +0.14 pp).

These results suggest that activation ensembling is most useful for the attention head and also helps the gated head on TEST2. Its effect on concat is limited.

#### 4.3.3. VAL-Driven Stacking (Meta-Learner Trained on VAL)

Stacking variants are trained using only the VAL set. First, the top heads are selected as base models and their temperature-calibrated probability outputs on VAL are concatenated. Then, a multinomial logistic-regression meta-learner is trained using the VAL set.

Best single head (by TEST): 0.9933 (gated_fp_ens)
Super-ensemble of best heads: 0.9928 (super_bestheads)
VAL-driven stacks (TEST): top-2 FP: 0.9923
  top-3 FP: 0.9928
  top-2 no-FP: 0.9923
  top-3 no-FP: 0.9918
  top-3 mixed: 0.9928

Thus, the best VAL-driven stacks reach 0.9928, which matches the super-ensemble, but still remains −0.05 pp below the strongest single head on TEST (0.9933). Therefore, VAL-driven stacking does not provide a clear gain over the best non-stacked alternative.

#### 4.3.4. Rigorous TRAIN-Only OOF Stacking (Meta-Learner Trained on OOF)

The stacking meta-learner is trained using out-of-fold (OOF) predictions from the TRAIN split only. In each fold, the base heads are trained on a subset of TRAIN, tuned on a small inner split, and then used to predict the held-out fold. These OOF probabilities are used as input features for a multinomial logistic-regression meta-learner. Finally, the meta-learner is fit on all TRAIN-OOF features and evaluated on VAL, TEST, and TEST2.

OOF stacking does not improve TEST accuracy:

OOF stacks (single best activation per head inside each fold): TEST 0.9889–0.9904
OOF stacks (*ens*: within-fold activation ensemble): TEST 0.9894–0.9904

Compared to the best single head on TEST (0.9933), the best OOF stack (0.9904) is −0.29 pp lower.

#### 4.3.5. Generalization Under External Shift (TEST2)

All methods show lower performance on TEST2 than on TEST. This indicates a domain shift between the public data and the external clinical set. Across all variants, TEST2 accuracy ranges from 0.8768 to 0.9516. This represents an approximate 3.8 to 11.6 percentage drop compared to the TEST performance.

The best external performance is achieved by attn_fp_ens: TEST2 accuracy = 0.9516, TEST2 macro-F1 = 0.9411, and TEST2 detection accuracy = 0.9707.

#### 4.3.6. Calibration and Brier Score

Calibration performance is generally good on the TEST set. Among all variants, the lowest TEST ECE is 0.0017, achieved by stack_lr_full_top2_nofp. The lowest TEST Brier score is 0.0025, obtained by concat_nofp_single. However, these models do not achieve the highest classification accuracy. The best TEST accuracy is 0.9933, achieved by gated_fp_ens. This suggests that better calibration does not always result in higher accuracy.

#### 4.3.7. Takeaways

1. FP features are not equally useful for all heads. They clearly help the attention head. They also help the gated head in some cases, especially on TEST2. However, they do not help the concat head on external data and even reduce TEST2 performance.
2. Within-head activation ensembling helps the attention head the most. It also helps the gated head in several cases, especially on TEST2. For the concat head, the improvement is small and not consistent.
3. Stacking gives only limited improvement in this study. The best TEST result still comes from a best fusion-head ensemble, gated_fp_ens (0.9933). VAL-based stacking works well, but it does not beat the best fusion-head ensemble.
4. The more rigorous OOF stacking is also not useful in this setup. It does not improve performance beyond the best fusion-head ensemble.
5. TEST2 shows a clear domain shift. All models perform worse than on TEST. Among all variants, attn_fp_ens performs best on external data and achieves the highest TEST2 accuracy and macro-F1.

#### 4.3.8. 10-Fold Cross-Validation of the Best TEST2 Variant

For a more rigorous evaluation, we selected the attn_fp_ens variant because it achieved the best performance on the local clinical data. This variant was further evaluated using 10-fold cross-validation performed only on the TRAIN set. The out-of-fold (OOF) results on TRAIN achieved an accuracy of 0.9829 and a macro-F1 score of 0.9821. The 10-fold cross-validation results are summarized in Table 8.

**Figure 14:**
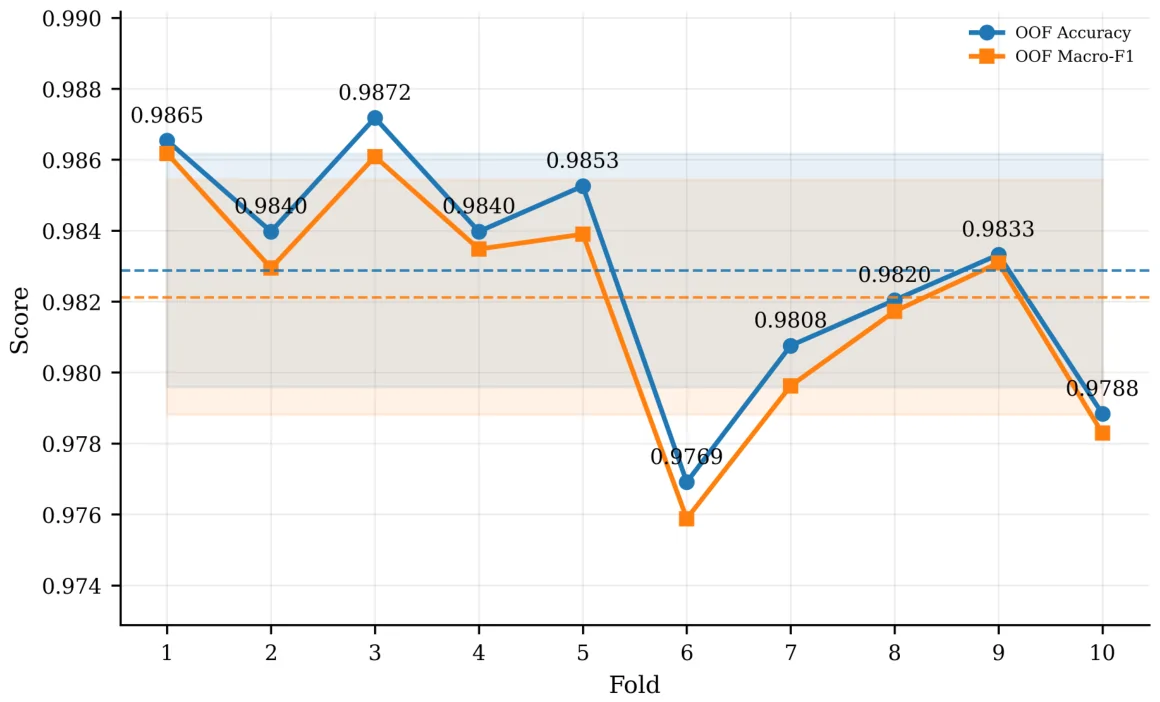
Fold-wise out-of-fold accuracy and macro-F1 scores across the 10-fold cross-validation for attn_fp_ens. Each marker represents the performance of an individual fold. The shaded regions indicate the variability across folds, and the dotted lines represent the mean accuracy and macro-F1 values.

**Figure 15:**
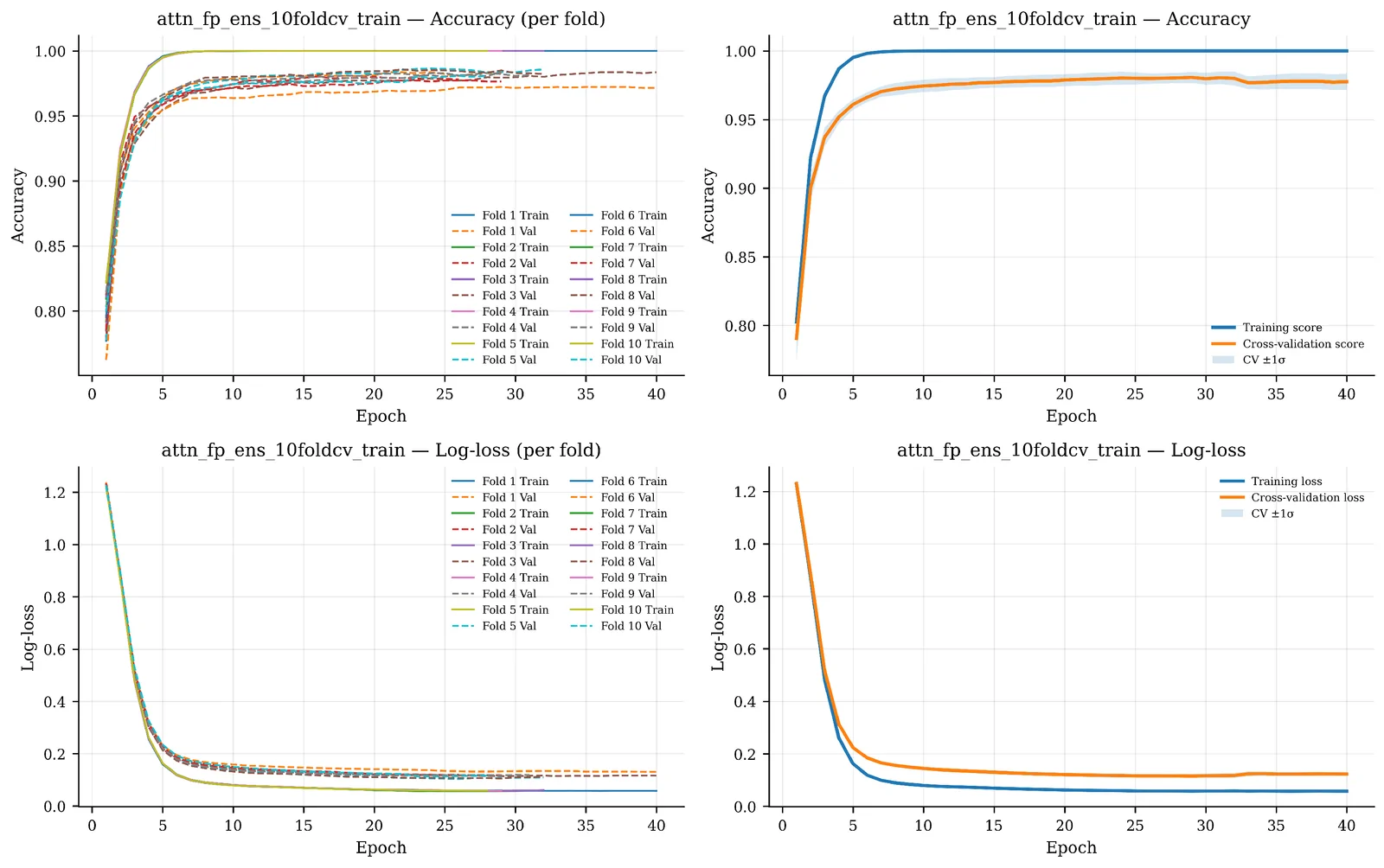
Synchronized learning curves from the 10-fold cross-validation for attn_fp_ens.

This configuration performs a 10-fold cross-validation on TRAIN only. The 10-fold CV learning curves are shown in Figure 14 and Figure 15.

#### 4.3.9. Single-Backbone Baseline Comparison

To compare the proposed multi-backbone model with individual feature extractors as baselines, each backbone was evaluated separately.

Among the single backbones, DenseNet201 achieved the best TEST macro-F1 (0.9531), followed by EfficientNetV2-M (0.9454). The proposed gated_fp_ens achieved a substantially higher macro-F1 of 0.9928, showing the benefit of combining multiple backbone features.

The comparison results between individual backbone models and the proposed multi-backbone model are summarized in Table 9.

### 4.4. Dataset-Source Predictability from Low-Intensity Background Features

To examine whether low-intensity background information could identify the dataset source, we used 77,719 MRI images from the nine source datasets (S1–S9). A 64-bin grayscale intensity histogram was calculated for each image, and only the first five lowest-intensity bins were used as features. The features were extracted from both the whole image and the outer 10% border, which mainly represents the dark background region.

Logistic Regression and Random Forest were evaluated using 5-fold grouped cross-validation repeated three times. Images with the same exact pixel hash were kept in the same fold to prevent identical images from appearing in both training and testing. Since there were nine dataset sources, the chance level was 11.11%.

The strongest results were obtained using Random Forest. Using the outer 10% border, the model achieved 33.05% accuracy and 16.89% balanced accuracy. Using the whole image, it achieved 34.12% accuracy and 16.95% balanced accuracy. Because the source datasets were highly imbalanced, balanced accuracy is more appropriate for interpretation.

Overall, these results indicate that low-intensity background information contains some dataset-source signal, but this information alone is not strong enough to reliably identify the dataset source. The dataset-source prediction results using low-intensity background features are summarized in Table 10.

### 4.5. Effect of Skull Stripping and Background Cropping

To investigate whether the models relied on non-brain information, we compared their performance on the original raw images and on skull-stripped and background-cropped images. The comparison was performed across all 28 model variants on both the internal TEST set and TEST2. The mean performance comparison between raw images and skull-stripped/background-cropped images across the 28 model variants is summarized in Table 11.

Overall, skull stripping and background cropping resulted in a small reduction in performance on the internal TEST set. Across the 28 evaluated variants, the mean accuracy decreased from 99.10% to 98.02%, while the mean macro-F1 decreased from 99.04% to 97.91%. In contrast, performance improved on TEST2, where the mean accuracy increased from 89.49% to 93.77%, and the mean macro-F1 increased from 88.63% to 91.99%.

Skull stripping and background cropping therefore slightly reduced the accuracy on the internal TEST set, but improved the accuracy on the TEST2 dataset. This suggests that the models trained on raw images may partially rely on non-brain information, such as the skull, image borders, background regions, or scanner- and dataset-specific patterns for prediction. Removing these regions may help the models focus more on the brain region and improve their robustness when evaluated on data obtained from a different source.

### 4.6. Dataset-Source-Held-Out Evaluation

To examine whether performance changes when the test data come from a completely unseen dataset source, we performed a leave-one-dataset-source-out (LODO) experiment using the gated_fp_ens model. In each fold, one complete dataset source was used only for testing, while the remaining sources were used for training and validation. Exact duplicate images shared with the held-out source were removed from the training and validation data before training each fold.

The performance of the model for each held-out dataset source is shown in Table 12.

Performance remained high for most held-out dataset sources. However, the lowest performance was observed for S5, where the model achieved 84.68% accuracy and 83.71% macro-F1. This suggests that differences between dataset sources can affect model generalization.

### 4.7. Extension to Joint Brain-Tumor Classification and Localization

Beyond tumor classification, identifying the tumor location is also important because a classification-only model provides only a class label and does not indicate where the abnormal region is located. Therefore, after evaluating the classification variants, this study further extended the framework into a joint brain-tumor classification and localization model. The extended model performs two tasks simultaneously: it predicts the tumor class and generates a binary mask representing the tumor region.

The joint model follows the Gated-FP concept because the gated-fusion model with fingerprint features showed the strongest performance on the internal TEST set in the previous classification analysis. The joint architecture therefore retains the same general gated-fusion idea and uses the same six pretrained backbone networks for feature extraction. However, instead of using only globally pooled features, spatial feature maps are preserved so that the same shared encoder can support both classification and localization. Sigmoid-based spatial gates control the contribution of the different backbone feature streams before fusion.

A 70-dimensional fingerprint descriptor is also included in the classification branch. It contains a 64-bin grayscale intensity histogram together with six statistical features: mean, standard deviation, skewness, kurtosis, entropy, and foreground ratio. This descriptor provides complementary information about the global intensity distribution of the MRI image.

The classification branch combines the fused deep representation with the fingerprint descriptor to predict one of the four tumor classes. In parallel, a U-Net-style localization decoder uses the shared spatial features and skip connections to progressively recover spatial information and generate the binary tumor mask. In this way, the model provides both tumor classification and tumor-region localization from the same MRI input.

**Figure 16:**
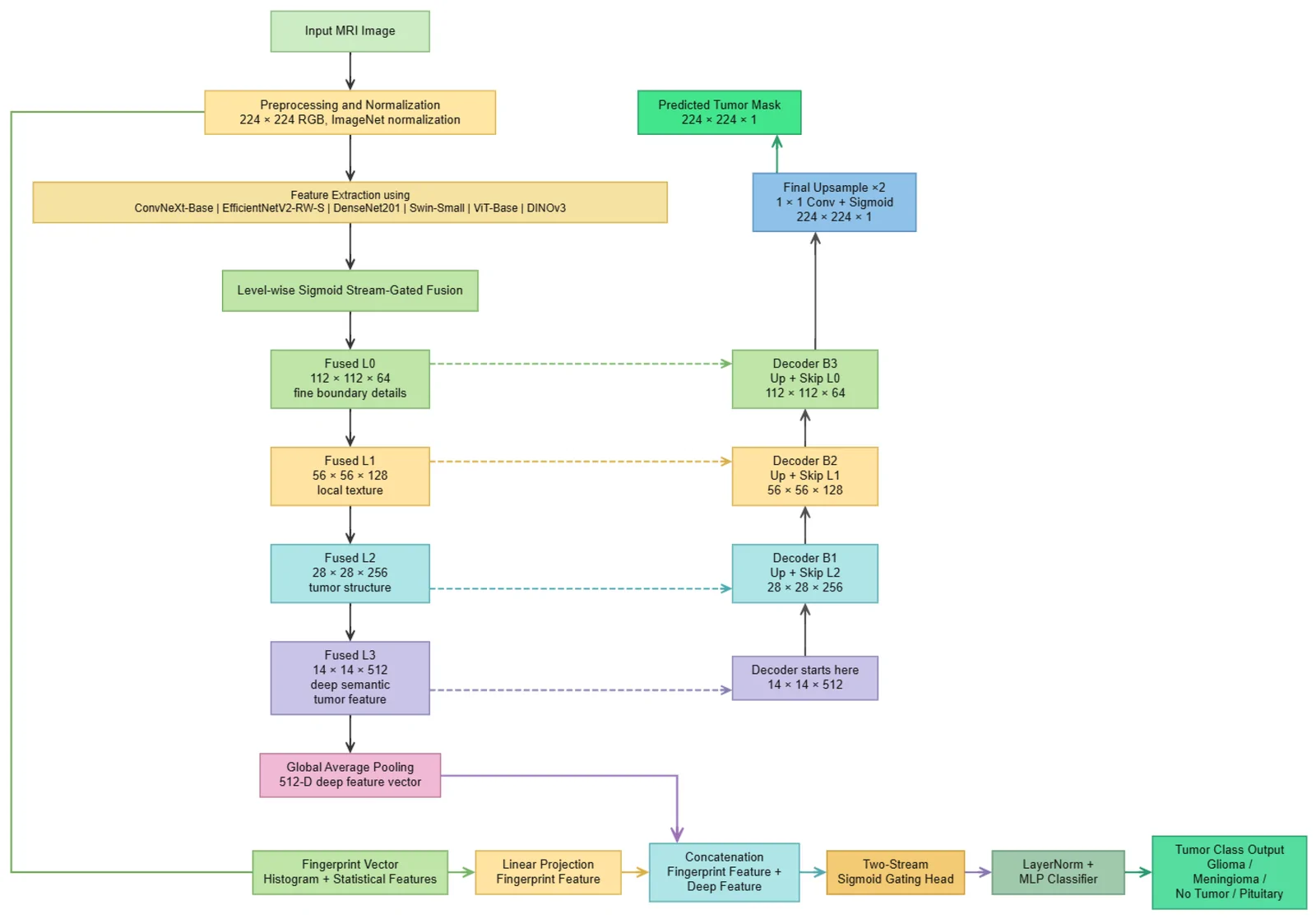
Architecture of the proposed joint brain-tumor classification and localization model based on the Gated-FP feature-fusion concept.

The overall architecture of the joint model is shown in Figure 16.

#### 4.7.1. Dataset for the Joint Experiment

The joint classification and localization experiment was conducted using the BRISC2025 brain MRI dataset [26,27]. This dataset is suitable for the joint task because it provides four-class brain MRI data together with tumor-region annotations for localization. The four classes are glioma, meningioma, no tumor, and pituitary tumor. The official training and testing data were used, while part of the training data were reserved for validation. The availability of both class labels and tumor masks makes this dataset appropriate for evaluating classification and localization within the same framework.

#### 4.7.2. Input Processing and Joint Architecture

Each MRI image was resized to 224×224 pixels and normalized before being passed through the shared multi-backbone encoder. During training, light MRI-safe augmentation was applied, including horizontal flipping, small rotations, and minor brightness and contrast changes.

The shared encoder extracts spatial features from the six pretrained backbones. These features are projected to compatible dimensions and combined using sigmoid-based spatial gating. The fused representation is then used by two branches. The classification branch combines the deep features with the 70-dimensional fingerprint descriptor, whereas the localization branch uses a U-Net-style decoder with skip connections to produce the predicted tumor mask.

#### 4.7.3. Loss Functions and Joint Training

The classification branch was trained using cross-entropy loss with label smoothing of ϵ=0.05 to reduce overconfident predictions. MixUp was also applied to the classification batches with α=0.20. MixUp was not applied to the segmentation masks because mixing binary masks could distort the true tumor boundaries.

For two classification samples, the mixed input is defined as

(15) $$X_{\mathrm{mix}}=\lambda X_{i}+\left(1-\lambda \right)X_{j},\;\lambda ∼\mathrm{Beta}\left(0.20,0.20\right).$$

The corresponding fingerprint vectors and class labels were mixed using the same coefficient.

For tumor localization, the segmentation branch combines binary cross-entropy (BCE), Dice loss, and focal loss. BCE provides pixel-wise supervision, Dice loss encourages overlap between the predicted and ground-truth tumor regions, and focal loss gives greater emphasis to difficult foreground pixels. The segmentation loss is defined as

(16) $$L_{\mathrm{seg}}=L_{\mathrm{BCE}}+L_{\mathrm{Dice}}+0.5\;L_{\mathrm{Focal}},$$

where the focal-loss parameters were set to α=0.25 and γ=2.0.

The classification and localization objectives were optimized jointly using

(17) $$L_{\mathrm{total}}=\lambda_{\mathrm{cls}}L_{\mathrm{cls}}+\lambda_{\mathrm{seg}}L_{\mathrm{seg}},$$

where $\lambda_{\mathrm{cls}}=1.0$ and $\lambda_{\mathrm{seg}}=1.0$. Thus, both tasks were given equal importance during training.

During each training iteration, one classification minibatch and one segmentation minibatch were processed before a single optimizer update. Therefore, gradients from both tasks contributed to the same shared encoder, allowing the learned representation to support both classification and localization.

The model was optimized using AdamW with a learning rate of $2\times 10^{-4}$ and a weight decay of $5\times 10^{-5}$. A ReduceLROnPlateau scheduler was used to reduce the learning rate when the joint validation performance stopped improving.

#### 4.7.4. EMA and Checkpoint Selection

An exponential moving average (EMA) of the model parameters was maintained during training to obtain a smoother and more stable set of model weights. The EMA update is expressed as

(18) $$\theta_{\mathrm{EMA}}^{\left(t\right)}=\rho \theta_{\mathrm{EMA}}^{\left(t-1\right)}+\left(1-\rho \right)\theta^{\left(t\right)},$$

where the decay value was set to ρ=0.999. The EMA weights were used during validation and final evaluation.

Because the model performs both classification and localization, checkpoint selection was based on a joint validation score rather than classification accuracy alone. Classification accuracy and segmentation Dice score were given equal importance:

(19) $${\mathrm{Score}}_{\mathrm{joint}}=0.5\;{\mathrm{Acc}}_{\mathrm{val}}^{\mathrm{cls}}+0.5\;{\mathrm{Dice}}_{\mathrm{val}}^{\mathrm{seg}}.$$

The checkpoint with the highest joint validation score was selected for the final evaluation. This selection strategy ensures that the chosen model maintains balanced performance across both classification and localization.

#### 4.7.5. Evaluation

Classification performance was evaluated using accuracy, precision, recall, F1-score, and the confusion matrix. Localization performance was evaluated using the Dice score and visual comparison between the predicted and ground-truth tumor masks.

### 4.8. Tumor Localization and Classification Results

The joint multitask framework was evaluated for both tumor classification and localization.

Classification performance was evaluated using accuracy, precision, recall, F1-score, and the confusion matrix. Localization performance was evaluated using the Dice score, which quantifies the overlap between the predicted tumor mask and the corresponding ground-truth mask.

Qualitative overlay visualizations were also generated to assess tumor localization. These visualizations show the original MRI image, the ground-truth tumor mask, the predicted tumor mask, and the combined overlay, allowing visual comparison of the predicted and annotated tumor regions.

**Figure 17:**
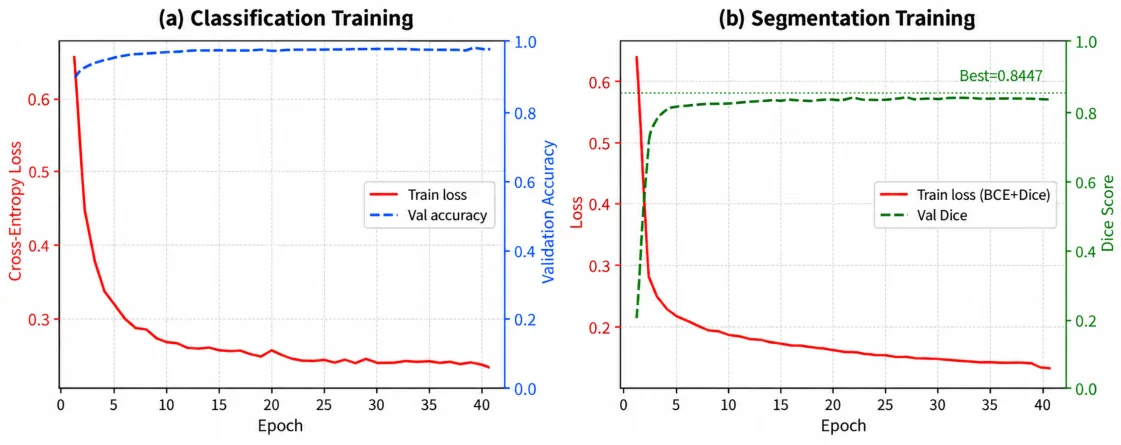
Training and validation performance of the proposed multitask model.

As shown in Figure 17, the training and validation curves illustrate the learning behavior of the multitask model.

**Figure 18:**
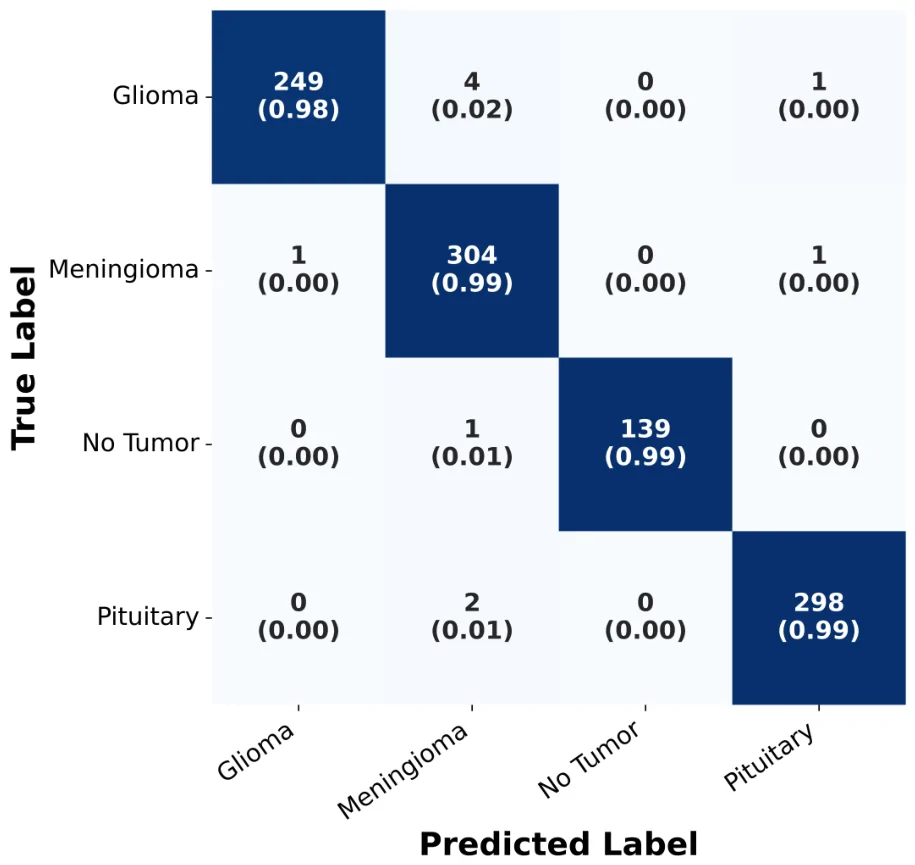
Confusion matrix for brain-tumor classification.

The classification performance is further illustrated by the confusion matrix in Figure 18.

**Figure 19:**
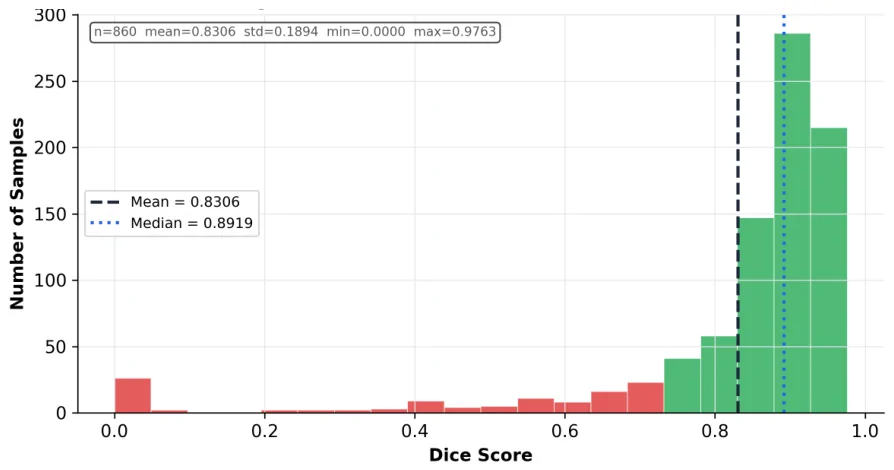
Distribution of Dice scores for tumor localization.

**Figure 20:**
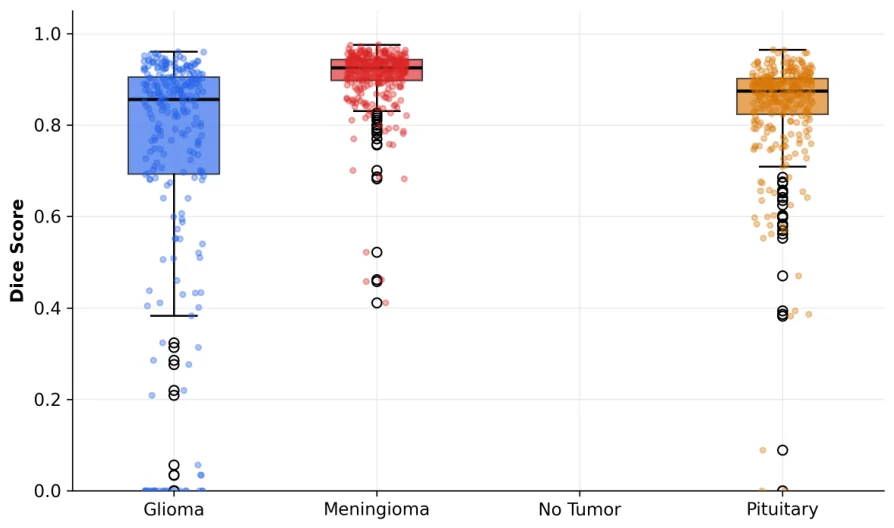
Class-wise distribution of Dice scores for tumor localization. Colored circles indicate individual Dice score values, and black circles indicate boxplot outliers.

Figure 19 shows the distribution of Dice scores across the evaluated segmentation samples, whereas Figure 20 compares the Dice-score distributions across tumor classes.

**Figure 21:**
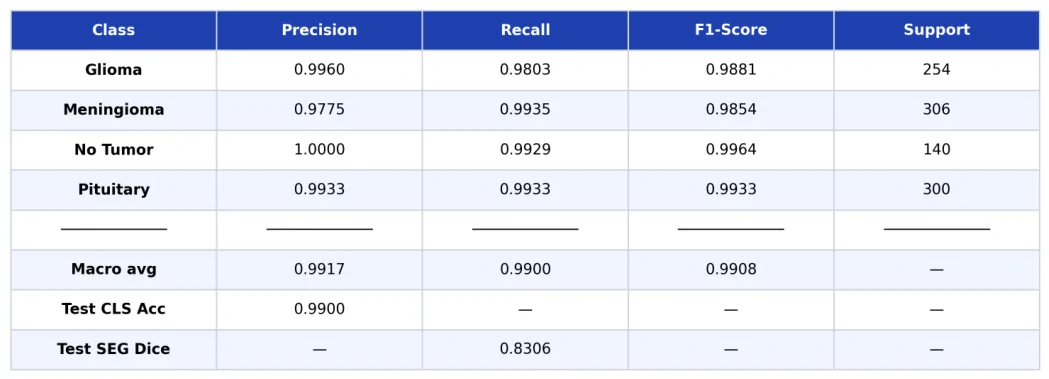
Summary of classification and tumor-localization performance.

The overall classification and segmentation results are summarized in Figure 21.

**Figure 22:**
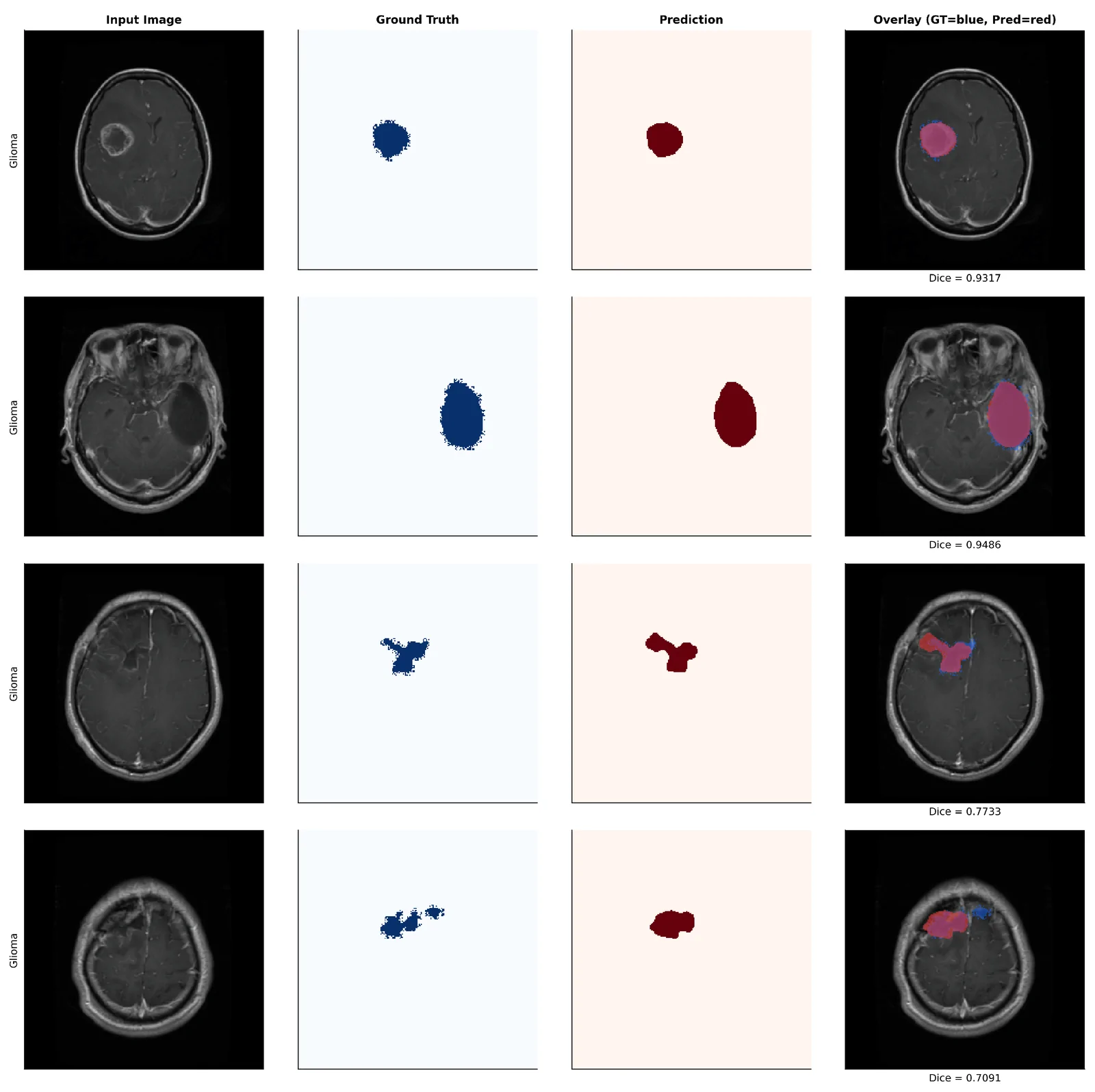
Qualitative tumor-localization results showing the original MRI image, ground-truth tumor mask, predicted tumor mask, and combined overlay visualization.

Qualitative localization examples are presented in Figure 22, showing the original MRI image, ground-truth mask, predicted mask, and combined overlay visualization.

### 4.9. Interpretability Analysis Using XAI Techniques

Interpretability is important in medical-image analysis to gain clinician trust in the particular diagnosis model. To gain their trust, it is necessary to show them how, or by considering which region the model predicts the diagnosis. A model may achieve high accuracy, but clinicians may not fully trust it if they cannot see which part of the MRI influenced the decision. For this purpose, an XAI experiment was conducted on the classification branch of the proposed gated_fp_ens model. XAI allows clinicians to examine whether the model is using a medically reasonable image region. The applied XAI techniques included Grad-CAM, Grad-CAM++, HiResCAM, LayerCAM, XGrad-CAM, Integrated Gradients, GradientSHAP, Score-CAM, Ablation-CAM, patch occlusion, and LIME. These methods produced visual maps showing which MRI regions contributed to the predicted tumor class.

To make the evaluation among those methods, an analysis was performed on a randomly selected set of 21 tumor images from test data, consisting of 7 glioma, 7 meningioma, and 7 pituitary cases. Among these, 19 correctly classified images were used in the main comparison. The XAI methods were compared not only by faithfulness, but also by how well and how precisely they highlighted the actual tumor region. This is important because a method may appear to perform well simply by highlighting a very large area of the brain. Such a broad heatmap may overlap with the tumor, but it is not clinically focused or visually convincing. Therefore, both tumor-region agreement and spatial focus were considered in the evaluation.

### 4.10. XAI and Model-Focus Analysis

The quantitative comparison of the evaluated XAI techniques is presented in Table 13. Among the evaluated methods, Grad-CAM++ showed the strongest overall balance between tumor-region agreement, spatial focus, and faithfulness. It achieved a tumor-sized Dice score of 0.434, an IoU of 0.324, a Top-10 precision of 0.266, an energy lift of 4.735, a pixel average precision of 0.464, and a faithfulness gain of 0.240 over the random reference. Unlike several other methods, Grad-CAM++ produced relatively focused attribution maps without highlighting unnecessarily large regions of the brain.

Score-CAM showed relatively strong faithfulness, with a faithfulness gain of 0.281, but its attribution maps were more spatially diffuse. LayerCAM and Grad-CAM achieved tumor-sized Dice and IoU scores similar to those of Grad-CAM++, although LayerCAM produced relatively diffuse maps and Grad-CAM showed a lower faithfulness gain. LIME obtained the highest faithfulness gain of 0.393, but its maps were excluded by the quality-screening criterion because they were spatially diffuse. Therefore, considering the quantitative metrics together with spatial focus, Grad-CAM++ was selected as the most suitable XAI method among those evaluated for the proposed gated_fp_ens classifier.

**Figure 23:**
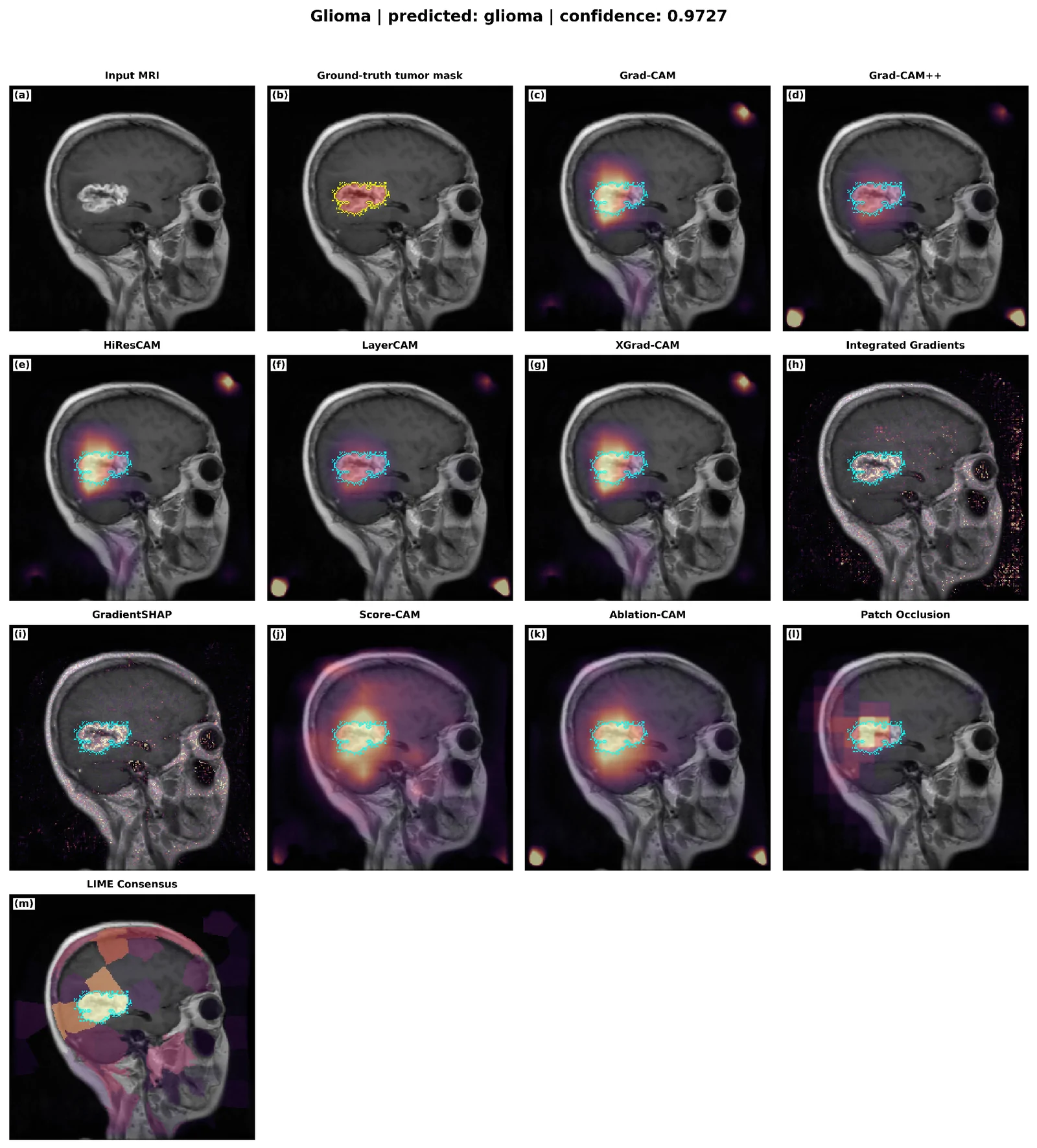
Visualization of all evaluated XAI methods for a glioma MRI image.

**Figure 24:**
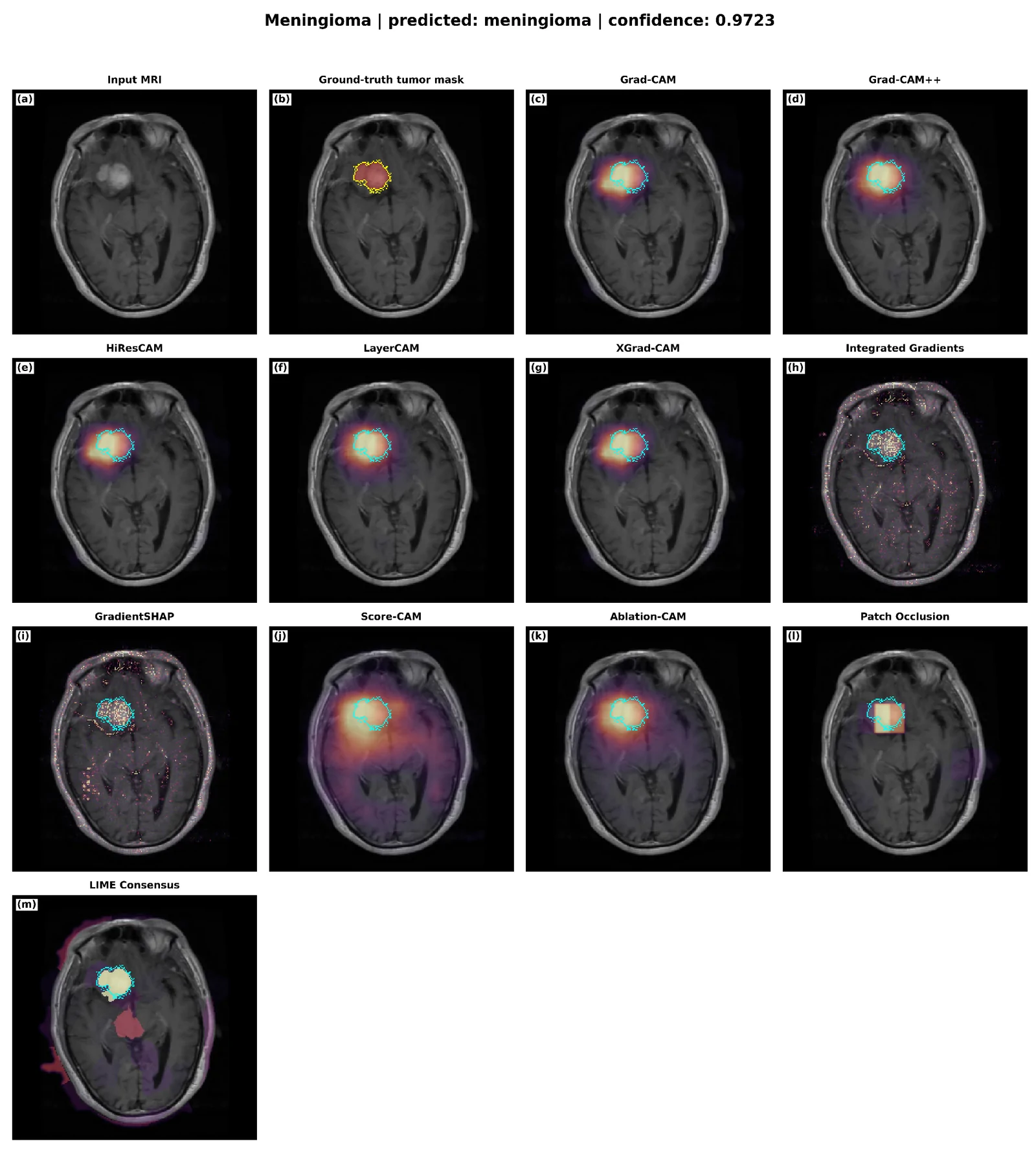
Visualization of all evaluated XAI methods for a meningioma MRI image.

**Figure 25:**
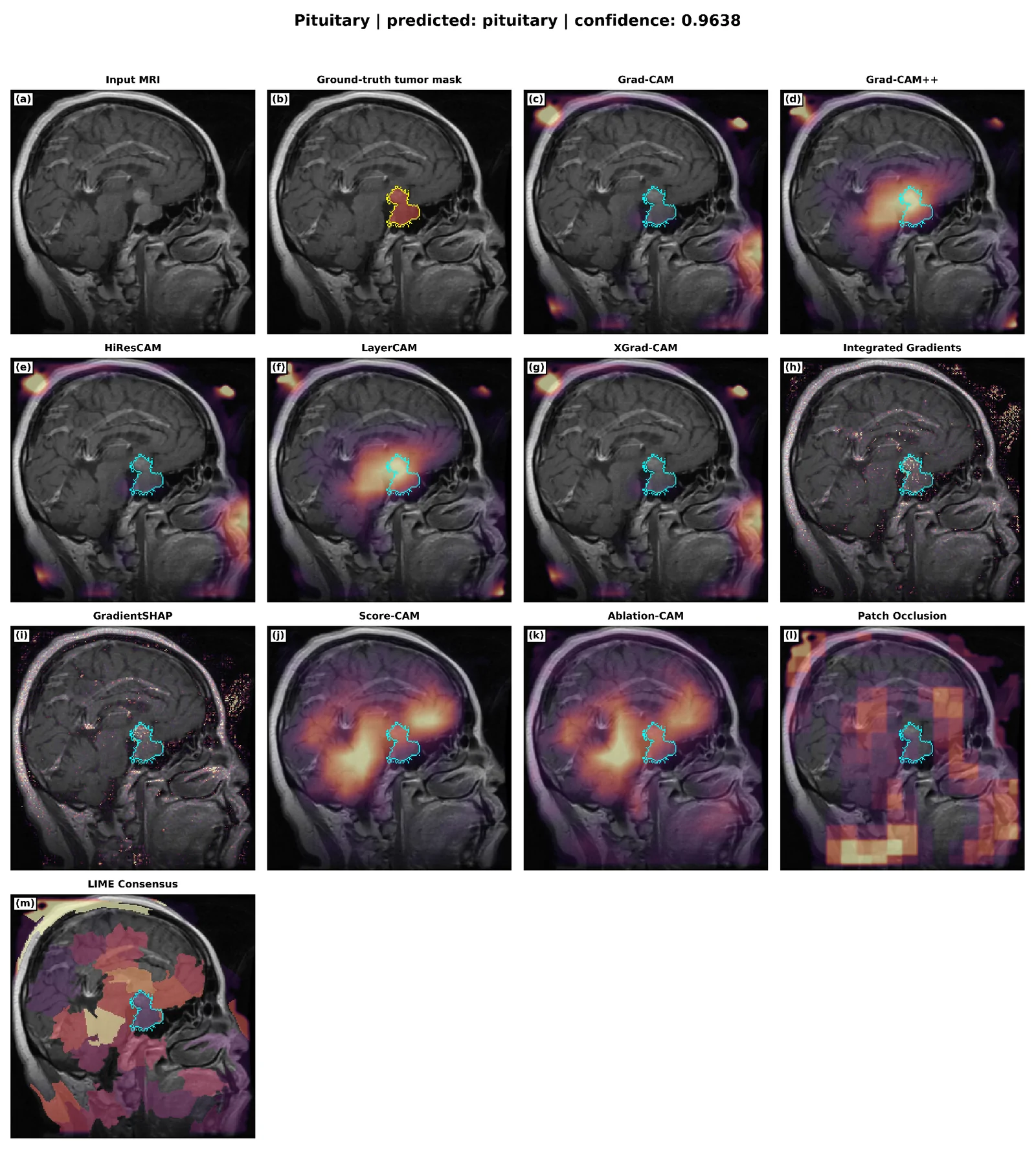
Visualization of all evaluated XAI methods for a pituitary tumor MRI image.

Visualization of all evaluated XAI methods is shown in Figure 23, Figure 24 and Figure 25. Specifically, Figure 23 presents a representative glioma case, Figure 24 presents a representative meningioma case, and Figure 25 presents a representative pituitary tumor case. These visualizations provide qualitative insight into the regions considered important by different explanation methods for the model predictions.

Overall, the XAI analysis suggests that the classifier frequently assigns importance to lesion-related regions. This provides additional insight into the regions influencing the predictions of the classification branch. Grad-CAM and LayerCAM may also be used as supporting explanation methods, although Grad-CAM++ provided the best overall balance among the evaluated techniques.

As shown in Figure 23, Figure 24 and Figure 25, the heatmaps highlight the image regions that contributed to the model prediction, with warmer colors indicating areas of greater importance. The sky-blue contour represents the ground-truth tumor boundary.

Therefore, based on both the quantitative evaluation in Table 13 and the qualitative visualizations in Figure 23, Figure 24 and Figure 25, Grad-CAM++ was selected as the most suitable XAI technique among the evaluated methods for interpreting the predictions of the proposed gated_fp_ens classifier.

### 4.11. Error Analysis

#### 4.11.1. Error Analysis on the Merged Publicly Available Dataset

**Figure 26:**
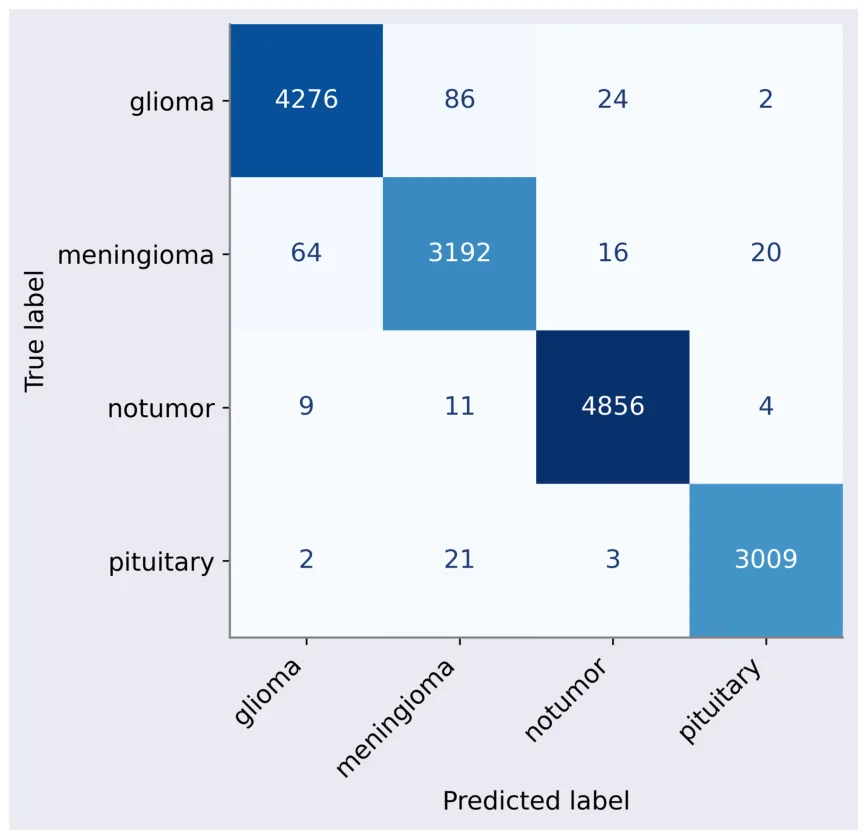
Meta-learner confusion matrix summed over folds.

The confusion matrix in Figure 26 summarizes the out-of-fold predictions across the 10-fold cross-validation procedure on the training split. It shows that, out of 15,595 images, the model correctly classified 15,333 and misclassified only 262 images (≈1.68% of the data). To better understand these errors, we examined all 262 misclassified cases together with a neurosurgeon co-author (Dr. Md. Ashraful Haque). Our discussions are summarized below.

Here, the model is essentially “thinking” in a clinico-radiological way. In real practice, the final decision about tumor type is made after surgery, using both histopathology and immunohistochemistry. In contrast, our model must make a decision from a single MRI slice.

We manually reviewed all 262 misclassified images together with our neurosurgeon. Interestingly, several of the misclassified cases are not straightforward even for human experts. The neurosurgeon explained that, for 5 of these misclassified images, he would also face confusion when trying to predict the tumor type from a single MRI slice. He also felt that for 83 misclassified images the model’s prediction looked reasonable (the neurosurgeon would likely make a similar prediction from a single slice) based on the limited information. In contrast, a portion of the remaining misclassifications were mainly due to very heavy noise and artifacts (27 images), which made them difficult for the model to interpret. For 18 additional cases, he noted that for them differential diagnosis (DD) would be needed before making a final decision.

A more detailed breakdown of the 262 misclassified cases is given below (T = true label, P = predicted label):

1.  T: glioma, P: meningioma (86 images):
  26 clinically reasonable; model and neurosurgeon would likely make a similar prediction from a single slice. 1 case where a differential diagnosis (DD) would be required. The remaining 59 images were judged as clearly wrong predictions.
2.  T: glioma, P: no tumor (24 images): 6 reasonable from a single slice; 5 heavily noisy images; the remaining 13 images were true wrong predictions.
3.  T: glioma, P: pituitary (2 images): 2 images were true wrong predictions.
4.  T: meningioma, P: glioma (64 images): 24 reasonable; 10 needing DD; 9 strongly affected by noise. 21 were actual wrong prediction.
5.  T: meningioma, P: no tumor (16 images): 10 reasonable; 2 noisy; 4 clear wrong prediction.
6.  T: meningioma, P: pituitary (20 images): 8 reasonable; 2 noisy; 10 clear wrong predictions.
7.  T: no tumor, P: glioma (9 images): 7 actual wrong predictions, 2 noisy images.
8.  T: no tumor, P: meningioma (11 images): 9 actual wrong predictions, for 2 images differential diagnosis (DD) would be required.
9.  T: no tumor, P: pituitary (4 images): 2 noisy; 2 wrongly cropped.
10. T: pituitary, P: glioma (2 images): 1 reasonable; 1 noisy.
11. T: pituitary, P: meningioma (21 images): 8 reasonable; 5 needing DD; 4 noisy; 4 clear wrong predictions.
12. T: pituitary, P: no tumor (3 images): 3 were actual wrong.

#### 4.11.2. External TEST2 Analysis

To further evaluate the robustness of the proposed variant, this study tested the attn_fp_ens model on the external TEST2 dataset. This evaluation included classification performance, calibration, class-wise errors, and detailed confusion patterns.

##### Overall Performance

As shown in Table 14, the model achieved an accuracy of 0.9516 with a bootstrap confidence interval of [0.9370, 0.9663]. It also obtained a macro-F1 score of 0.9411. These results show that the model maintained strong performance on the external hospital dataset, although its performance was lower than on the internal TEST set. The class-wise results in Table 15 further show that the model performed consistently well across all classes. From a probabilistic perspective, the model achieved an ECE of 0.0342 and a Brier score of 0.0249, as reported in Table 14. These values indicate good calibration. However, the calibration was slightly lower than that of the internal test set.

The error pattern is summarized in Table 16.

**Figure 27:**
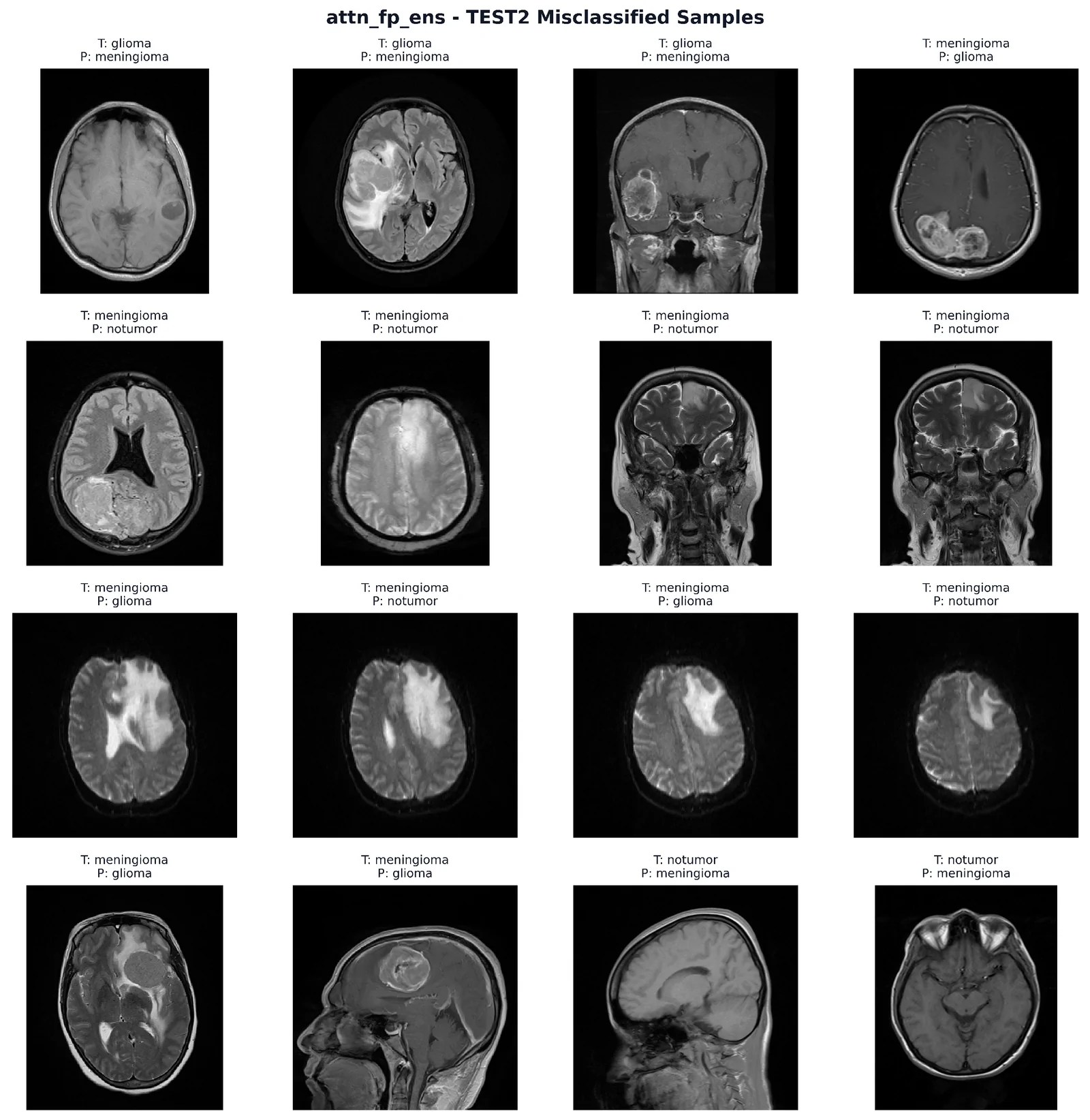
Examples of misclassified images on TEST2 by attn_fp_ens. Here, T denotes the true class, and P denotes the predicted class.

A total of 33 samples were misclassified. The most frequent confusion occurred between meningioma and notumor. Examples of misclassified images are shown in Figure 27.

##### Review of Misclassified Cases

To understand why these errors occurred, we reviewed the misclassified cases with our neurosurgeon co-author, Dr. Md. Ashraful Haque. In real clinical practice, the final tumor type is confirmed after surgery using histopathology and immunohistochemistry. In contrast, our model must make a decision from only a single MRI slice.

He explained that, to interpret the tumor class type based on a single slice, this inter-class tumor-type dilemma can be faced by an experienced neurosurgeon. In real clinical practice, the expert reviews multiple cuts and slices to figure out the actual tumor type. However, if they had to make a prediction like our model based on only a single slice, they would also face the inter-class tumor-type dilemma.

##### Visual Analysis

**Figure 28:**
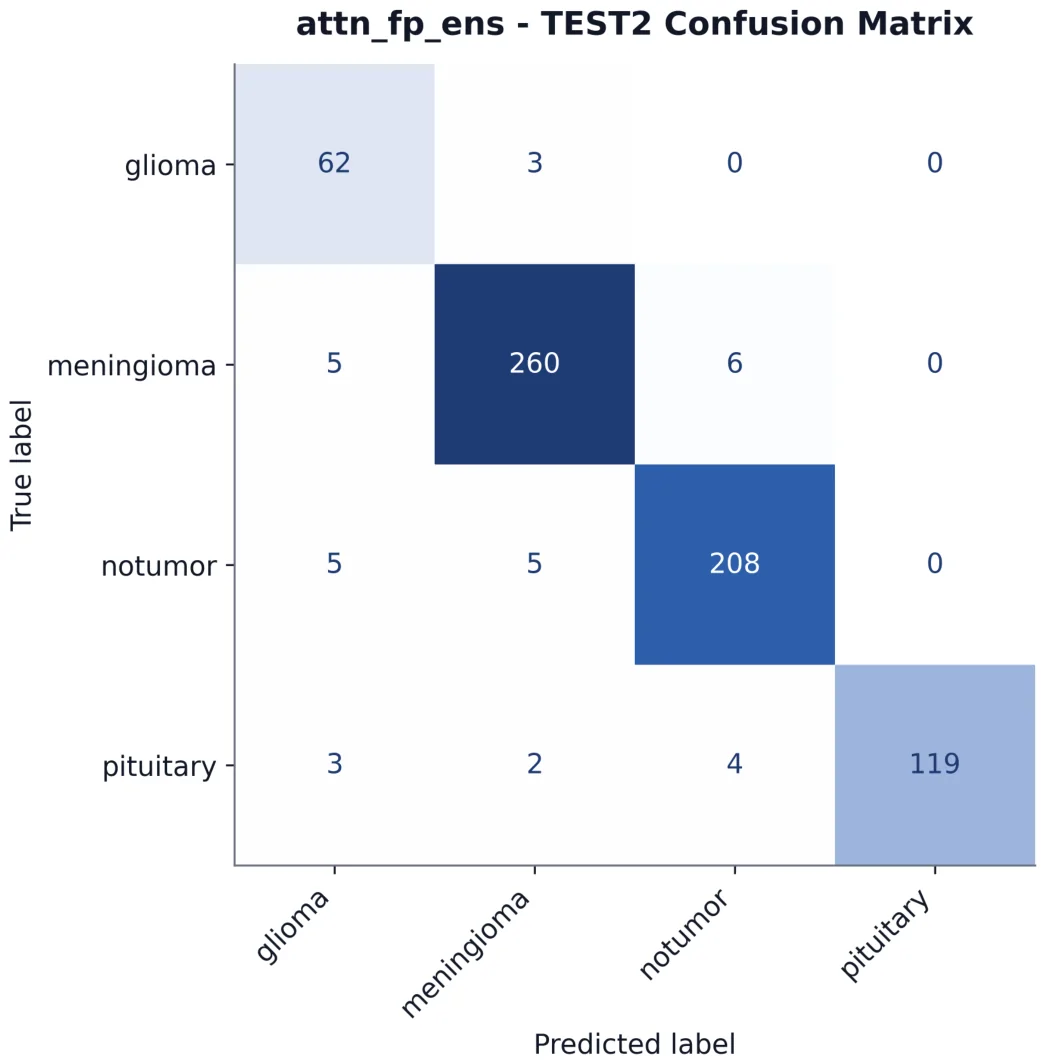
Confusion matrix on TEST2.

**Figure 29:**
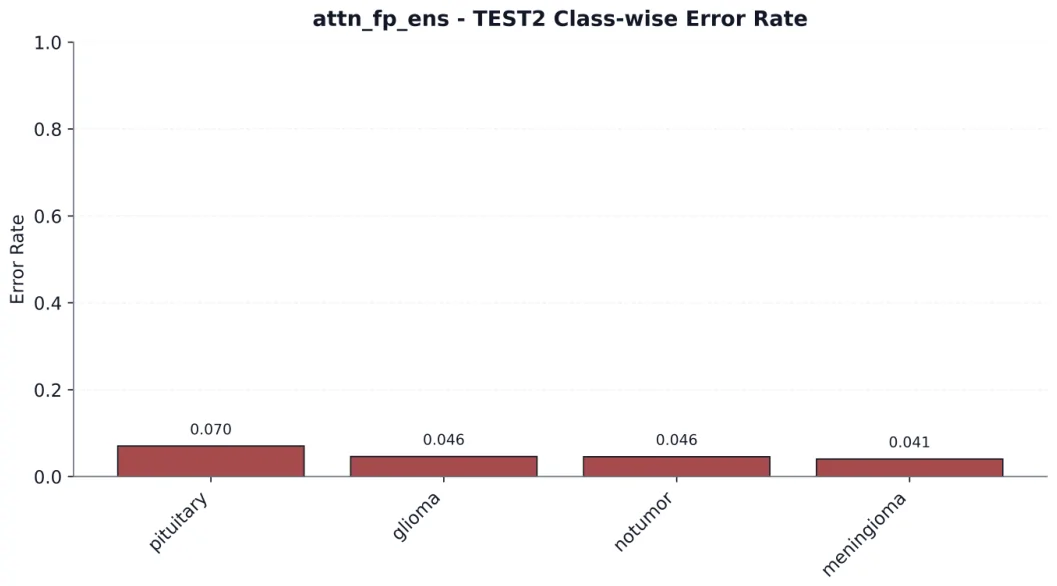
Class-wise error rate on TEST2.

**Figure 30:**
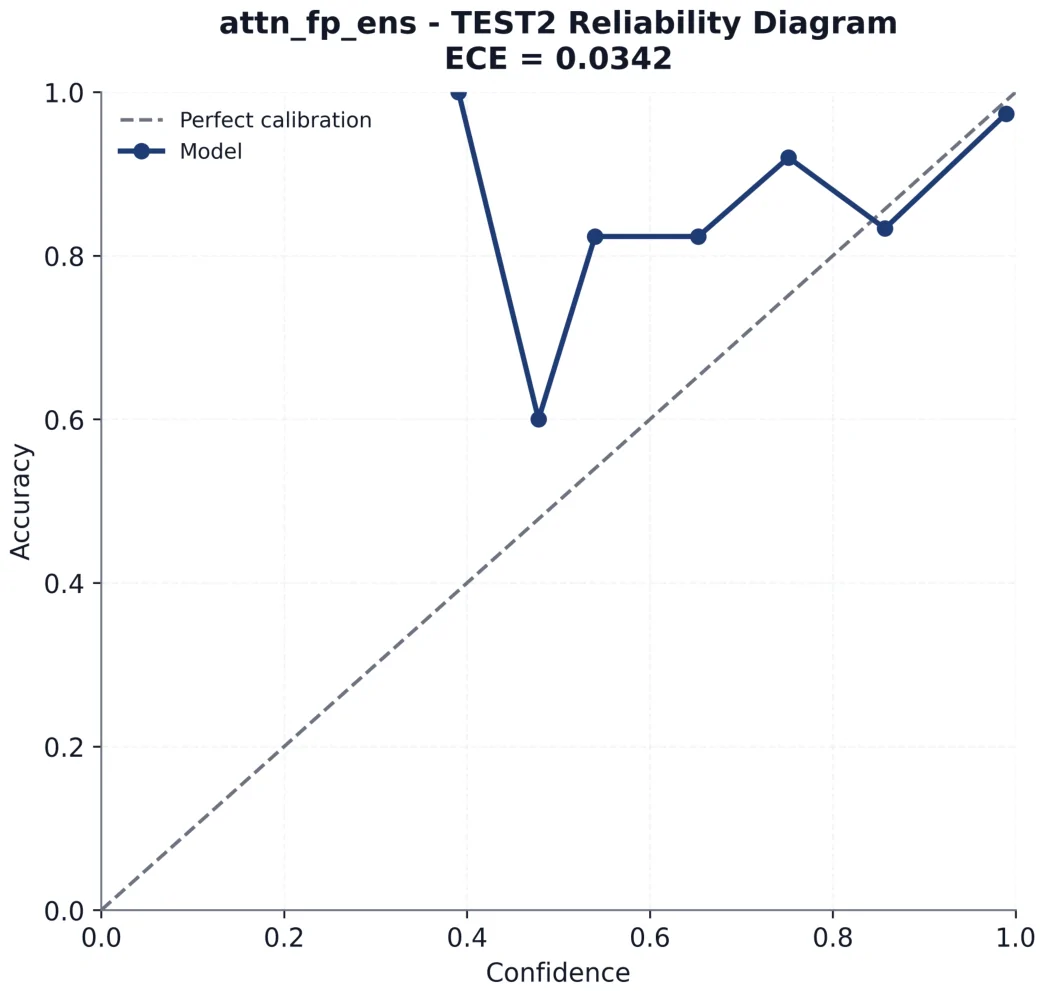
Reliability diagram on TEST2 (ECE = 0.0342).

**Figure 31:**
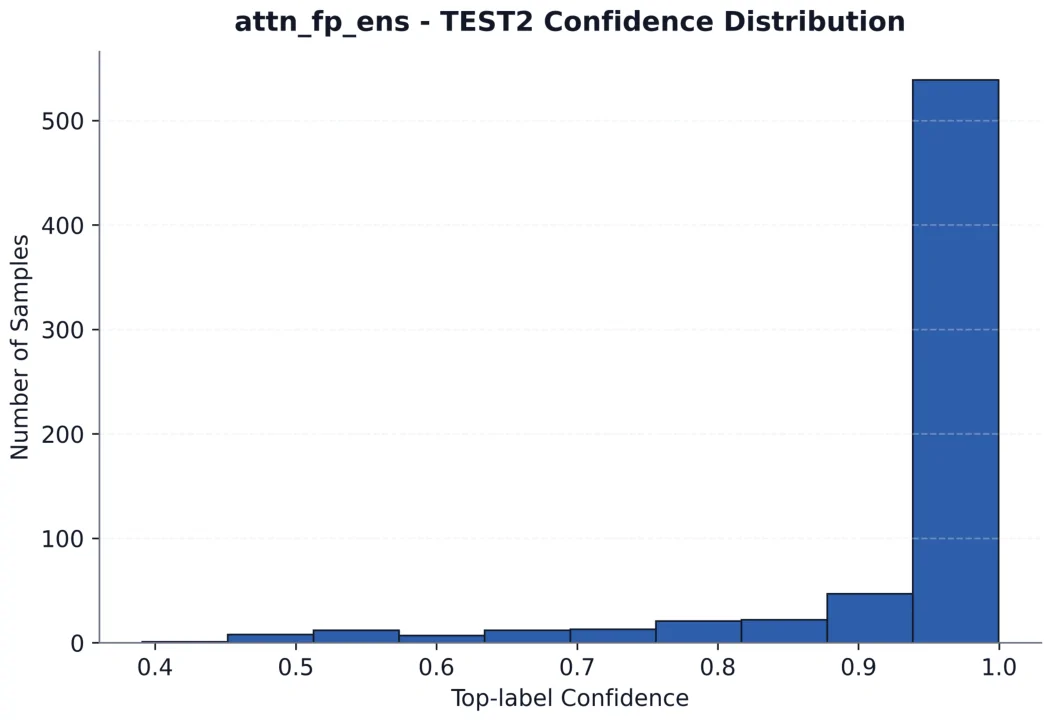
Confidence distribution on TEST2.

**Figure 32:**
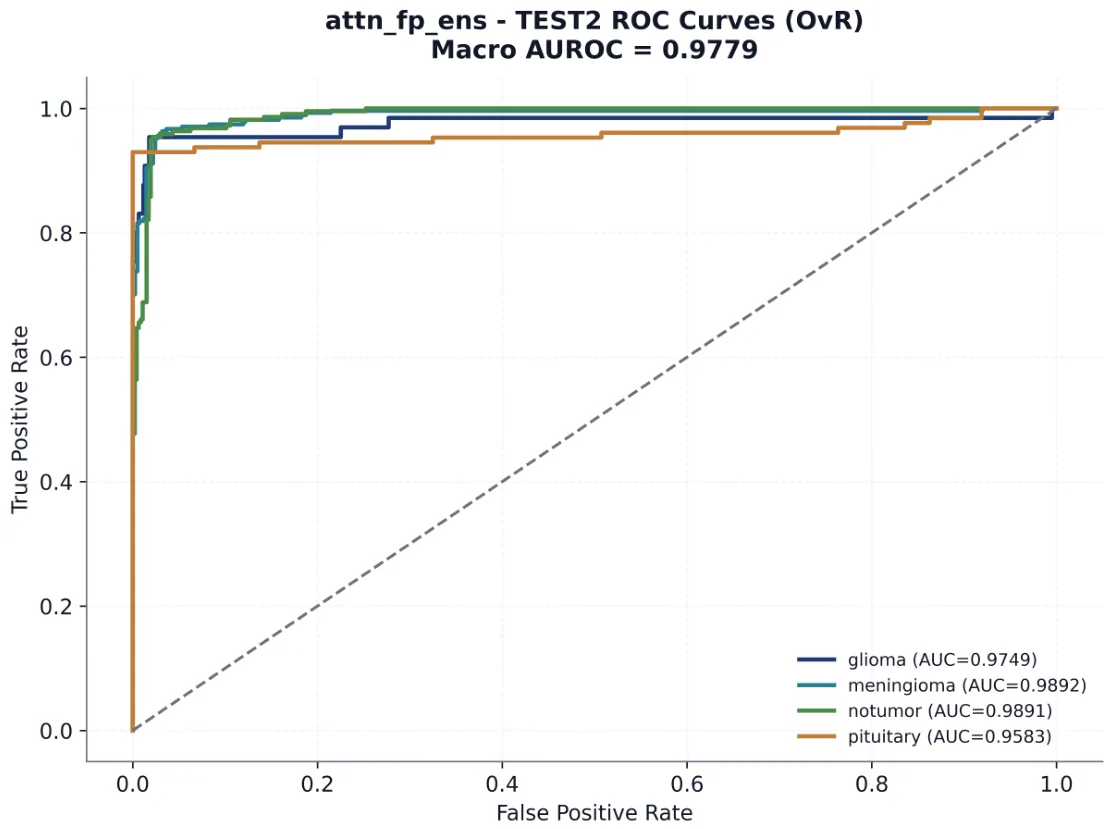
ROC curves (one-vs-rest) on TEST2.

**Figure 33:**
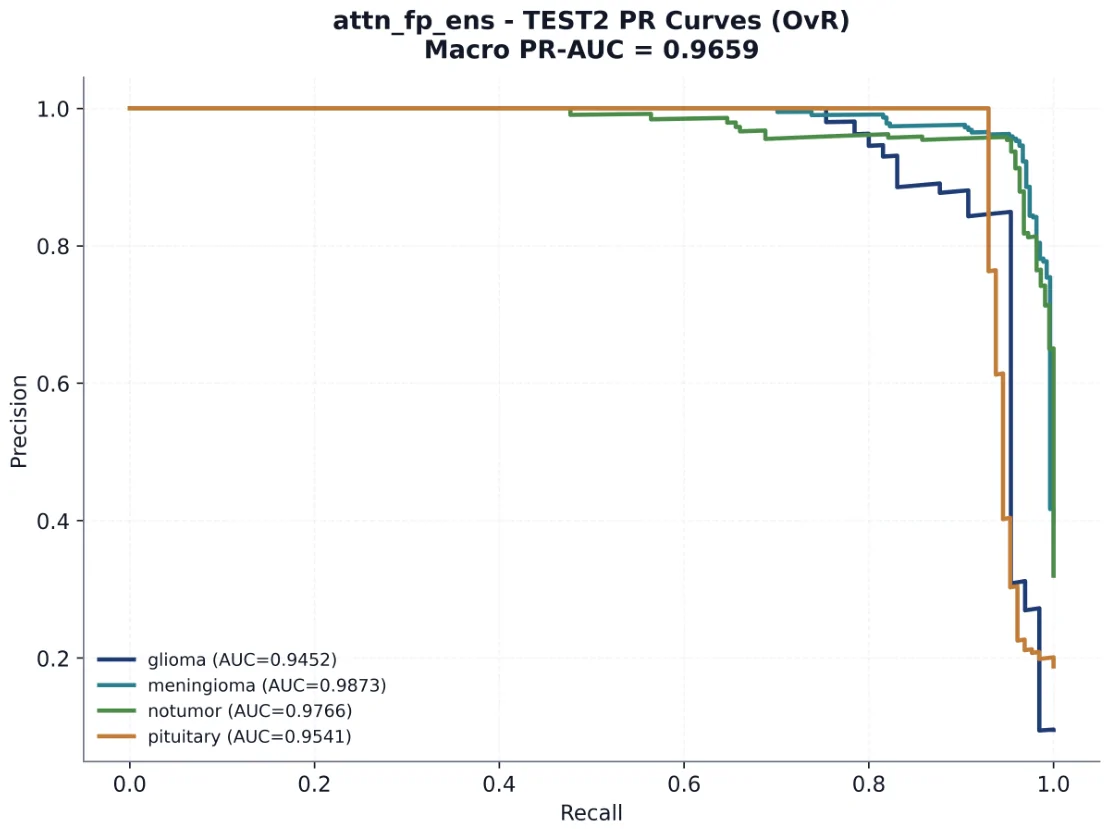
Precision–Recall curves (one-vs-rest) on TEST2.

**Figure 34:**
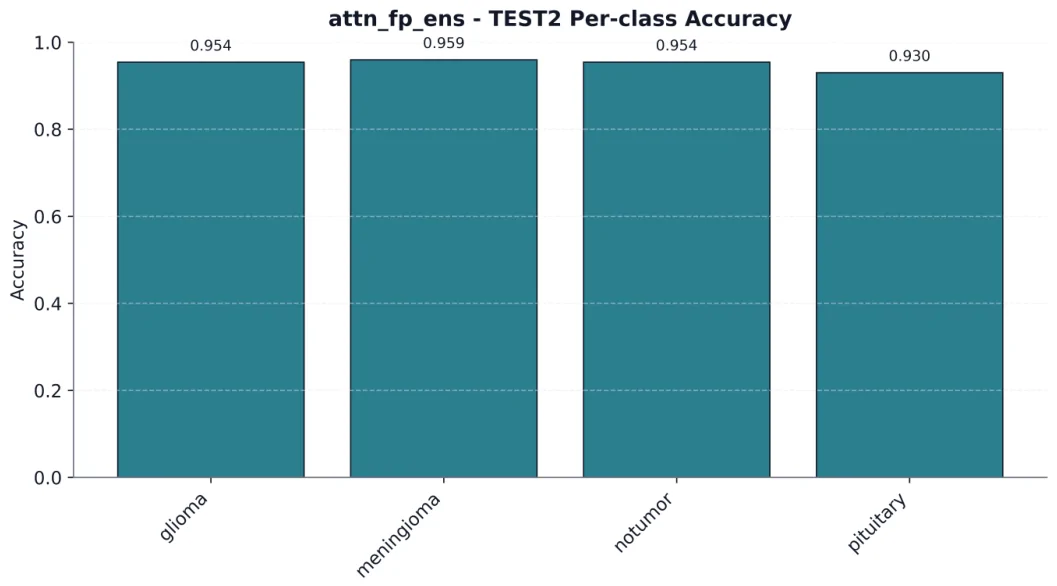
Per-class accuracy on TEST2.

Figure 28 shows the confusion matrix. Figure 29 presents the class-wise error rates, where pituitary has the highest error. Figure 30 shows the reliability diagram, indicating generally good calibration with slight underconfidence in several bins and minor overconfidence in some high-confidence bins. Figure 31 illustrates the confidence distribution of the predictions. Figure 32 and Figure 33 show the ROC and PR curves, confirming good performance across all classes. Figure 34 shows the per-class accuracy.

##### Discussion

Overall, the model maintained strong performance on TEST2, although the decrease relative to the internal TEST set indicates external domain shift.

### 4.12. Comparison of Training Time, Model Complexity, and Inference Speed

#### 4.12.1. Metrics and Protocol

Each head and meta-learner is evaluated based on three efficiency aspects: (i) training time (s), (ii) model size (M parameters), and (iii) head inference latency (ms/image). Since all variants use the same six frozen feature-extraction backbones, the backbone cost is common across variants and was excluded from this head-level efficiency comparison. The inference cost of the six frozen backbones is reported separately below.

#### 4.12.2. Inference Cost of the Six Frozen Backbones

To provide the computational cost of the complete feature-extraction stage, we separately measured the inference time of all six frozen backbones using 50 test images on the GPU. Without test-time augmentation, the combined six-backbone inference time was approximately 87.73 ms per image for raw images and 82.99 ms per image for skull-stripped images. With flip-based test-time augmentation, the corresponding inference times increased to 165.11 ms and 157.94 ms per image, respectively. The inference times are summarized in Table 17.

**Figure 35:**
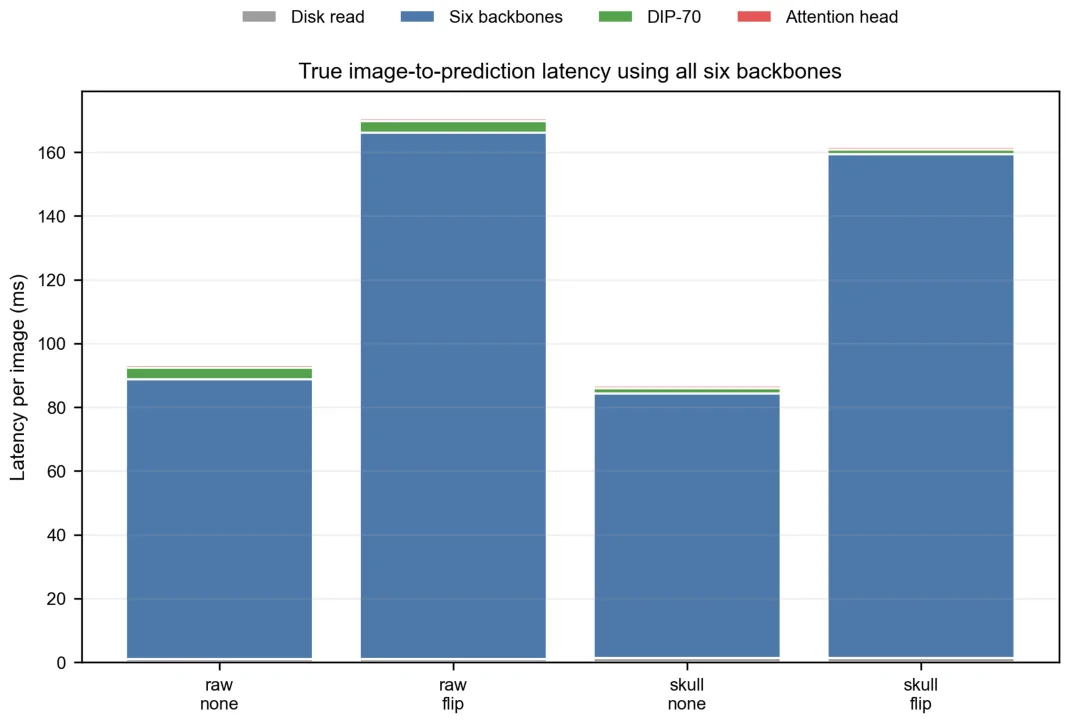
Inference-latency breakdown for raw and skull-stripped images with and without flip-based test-time augmentation. The six frozen backbones account for most of the computational cost, while the DIP-70 descriptor and attention-based classification head contribute relatively little additional latency.

Figure 35 further shows the contribution of disk reading, the six frozen backbones, the DIP-70 descriptor, and the attention-based classification head to the overall inference latency.

For a compact summary, this study reports an efficiency index (↑), computed as the mean of the three normalized scores for training time, parameter count, and inference speed:

$$\mathrm{Efficiency}=\frac{1}{3}\left({\mathrm{score}}_{\mathrm{train}}+{\mathrm{score}}_{\mathrm{params}}+{\mathrm{score}}_{\mathrm{speed}}\right)$$

where higher is better.

**Figure 36:**
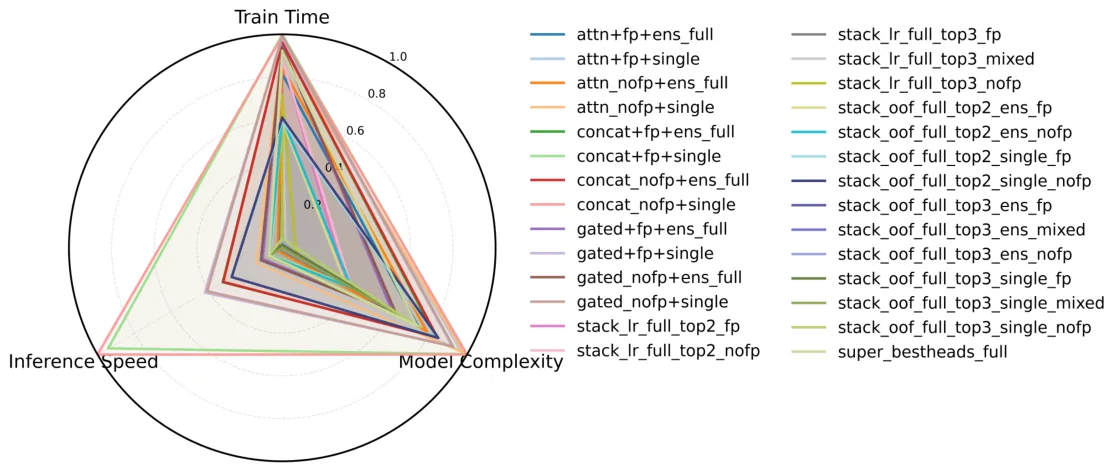
All variants in a single overlay under the same normalized efficiency axes: training time, parameters, and head inference speed (higher is better).

For a fair comparison (Table 18), methods that use multiple heads during inference, such as stack_lr_*, stack_oof_*, and super_bestheads, were evaluated by adding the parameter counts and inference latencies of their constituent heads. A single radar overlay for all variants is shown in Figure 36.

#### 4.12.3. Findings

**Figure 37:**
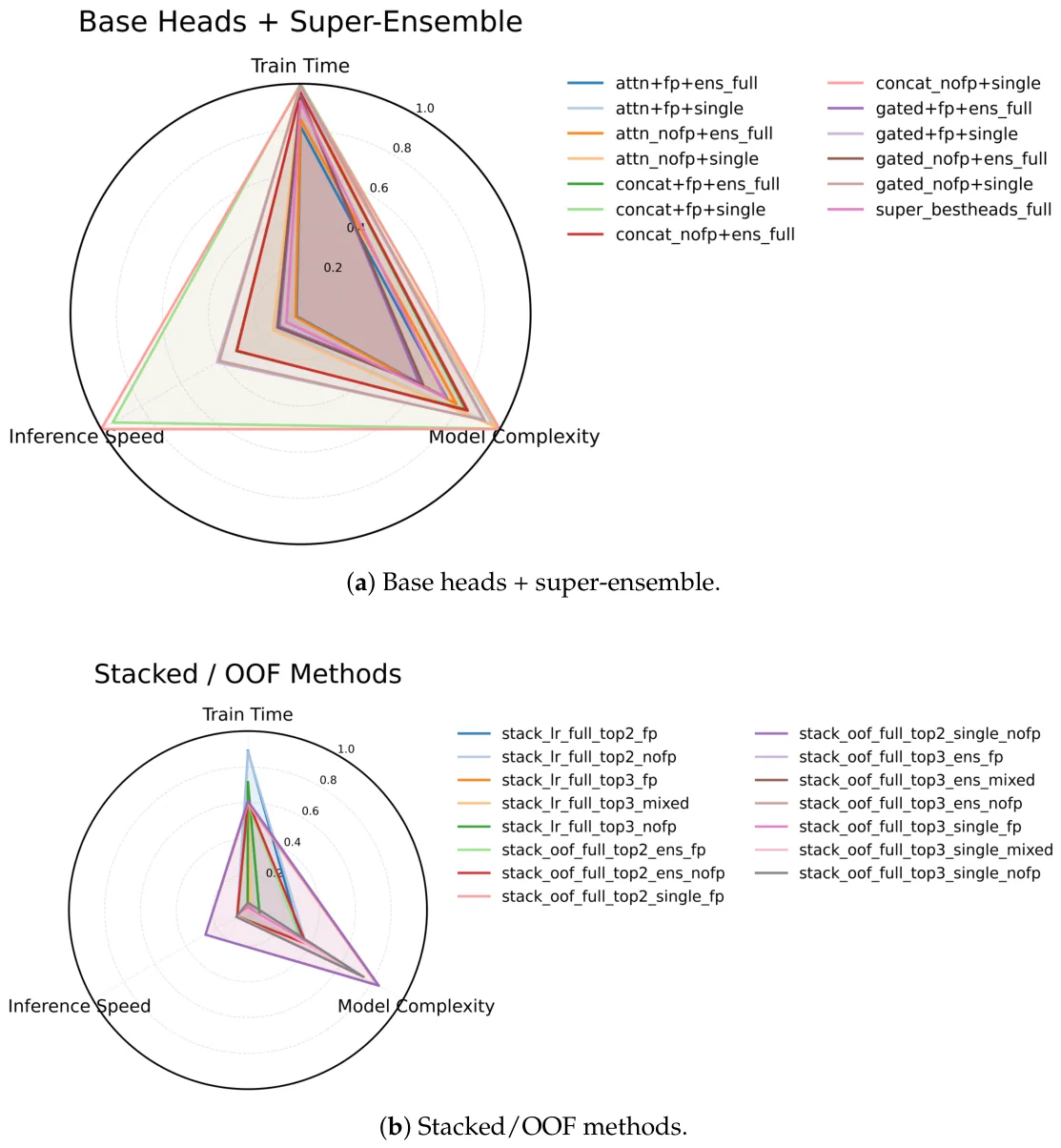
Normalized comparison of training time, model complexity (parameters), and head inference speed. Higher is better along each axis.

From Table 18, the highest overall efficiency is achieved by the single-head concat_nofp_single (1.000 efficiency, 6.69 M parameters, $6.86\times 10^{-4}$ ms/img, 116.1 s train), followed by concat_fp_single (0.935). The next most efficient variants are gated_nofp_single (0.750) and gated_fp_single (0.745). As shown in Figure 37, the normalized comparison of training time, model size, and inference speed shows that the concat-based single-head models are the most efficient options.

Within-head ensembles (*_ens) increase parameter count and latency. For example, concat_fp_ens uses 20.87 M parameters and requires 117.8 s training time. In terms of accuracy, the effect is mixed. Some ensemble variants provide little gain or even a slight drop compared with their single counterparts, whereas others, such as gated_fp_ens, improve TEST performance. Stacked methods have much larger model sizes, such as stack_lr_full_top2_fp with 61.20 M parameters and stack_lr_full_top3_fp with 89.95 M parameters. They also require substantially longer training times because they combine multiple base learners with a meta-learner. OOF-based stacks are the most expensive to train, taking about 0.9 k to 2.2 k seconds.

Cross-referencing with Table 7, the best TEST accuracy is obtained by the ensemble variant gated_fp_ens (0.9933). On the external TEST2 dataset, attn_fp_ens achieved the best performance, with an accuracy of 0.9516 and a macro-F1 score of 0.9411. In contrast, OOF stacking was the most computationally expensive approach. However, it still did not achieve the best TEST result. Its best configuration reached 0.9904, which was about 0.29 percentage points lower than gated_fp_ens.

#### 4.12.4. Practical Trade-Offs

For deployment settings where speed or memory is limited, the concat-based single heads are the most practical choice. In particular, concat_nofp_single and concat_fp_single provide the best balance among efficiency, model size, and inference speed. They also maintain strong TEST accuracy. If the main goal is to obtain the highest performance on the internal TEST set, and computational cost is not a major concern, gated_fp_ens is a strong option. However, if the main goal is better robustness on external data, attn_fp_ens is preferable. This model achieved the best result on TEST2, although it required more computational resources than the concat and gated single-head models.

### 4.13. Pairwise McNemar–Bowker Comparison on TEST (vs. Fixed Baseline)

**Figure 38:**
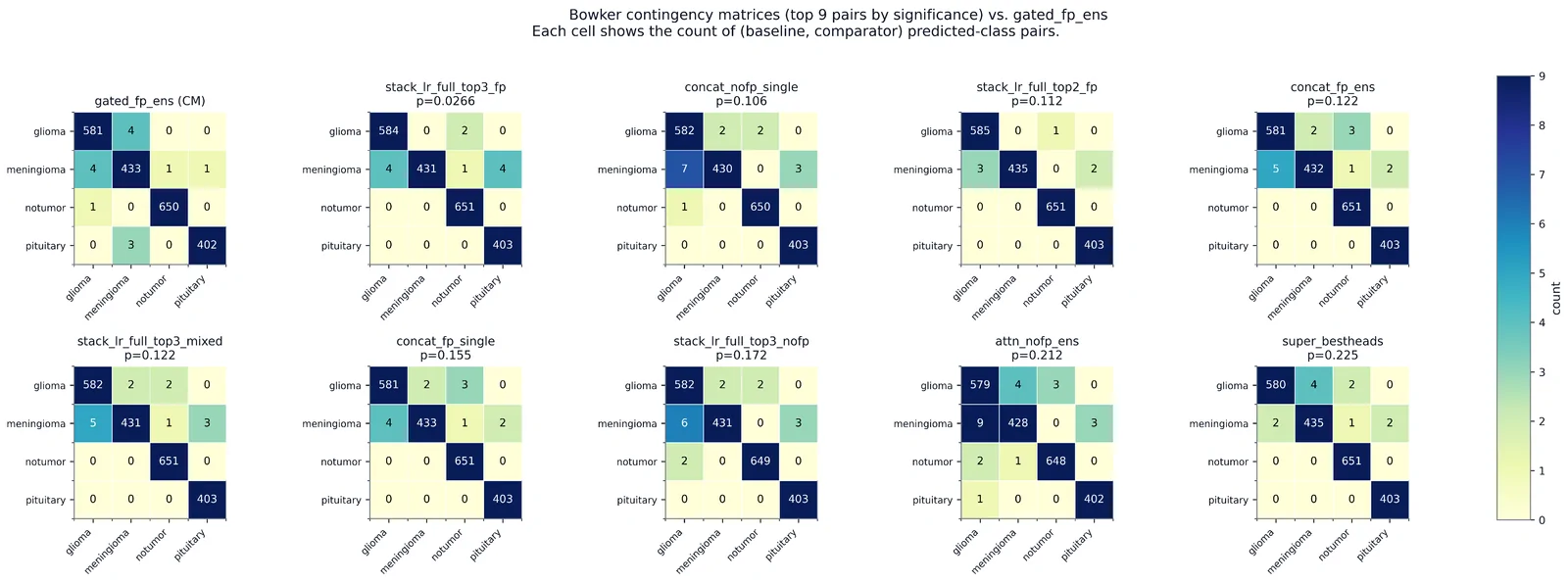
Bowker contingency matrices (baseline CM + top-*N* pairs by raw significance) versus gated_fp_ens on TEST. Each cell shows the count of (baseline, comparator) predicted-class pairs.

McNemar–Bowker tests were performed to assess whether the per-class error patterns of each comparator model differ from the fixed baseline (gated_fp_ens) on the same TEST samples. For each pair, Bowker’s $\chi^{2}$ statistic and raw *p*-value were computed, followed by the application of Holm–Bonferroni correction to maintain a family-wise error rate at α=0.05 across all 27 comparisons. The resulting *p*-values and test statistics are summarized in Table 19. The Bowker contingency matrices are visualized in Figure 38.

Summary.

As reported in Table 19, the smallest raw *p*-value in this run is p=0.0266 for stack_lr_full_top3_fp, which becomes $p_{\mathrm{Holm}}=0.7172$ after Holm correction. Thus, none of the 27 comparisons is statistically significant at α=0.05. Under family-wise error-rate control, the compared variants are therefore statistically indistinguishable from gated_fp_ens in terms of their error distribution across classes on the TEST set.

### 4.14. Uncertainty-Aware Abstention Analysis

In addition to standard classification performance metrics, the uncertainty-aware abstention behavior of each model variant is analyzed under a fixed confidence threshold. Using calibrated probabilities $\hat{p}\left(x\right)$, a confidence score $c\left(x\right)=\max_{k}{\hat{p}}_{k}\left(x\right)$ is defined and low-confidence cases are flagged as

$$\mathrm{flag}\left(x\right)=⊮\left[c(x)<\tau \right],\;\tau =0.70.$$

The number of flagged samples (“abstentions”) per model variant at τ=0.70 is reported. The flag is used only to identify cases for potential expert review.

**Figure 39:**
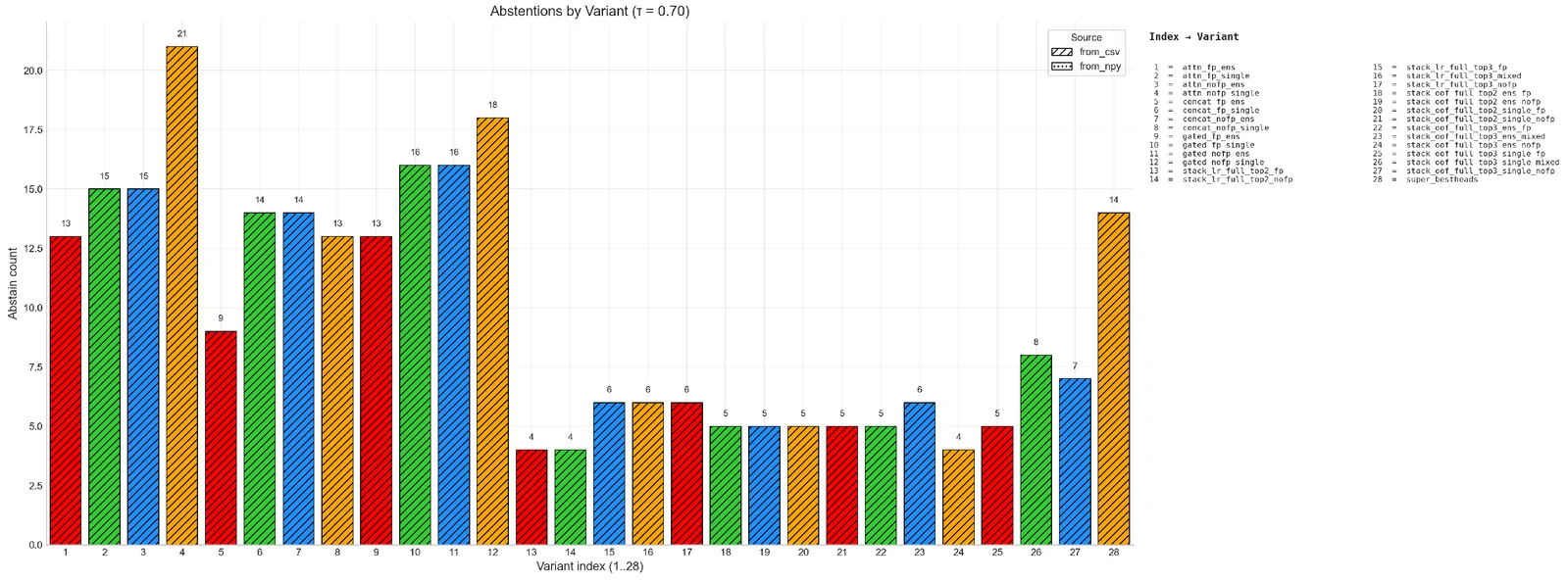
Abstentions by variant at confidence threshold τ=0.70 on the TEST set. A prediction is considered abstained if the maximum class probability is below τ.

Figure 39 reports the total number of abstained predictions for each model variant on the TEST set. Lower abstention counts indicate broader confident coverage, whereas higher counts reflect a more conservative decision strategy that favors reliability over coverage. To improve interpretability and avoid visual clutter, variants are indexed numerically on the horizontal axis, with a corresponding index-to-variant mapping provided alongside the plot.

### 4.15. Comparison to Prior Work on Benchmark Subsets

Table 20 shows that gated_fp_ens from the present study achieves 99.33% accuracy and 99.28% macro-F1 on the internal TEST set, using a dataset containing 20,794 cleaned images in total. Although some prior works report slightly higher peak accuracy on smaller benchmark subsets, many do not report F1-score or external validation, which limits robustness comparisons. Overall, the proposed model remains highly competitive.

Several Transformer-based baselines underperform classical CNNs on (four-class, 7022/7023 images). For instance, the plain ViT-B/16 architecture achieves only 91.36% accuracy [70], while enhanced variants like FTVT-l16 (98.70%) [68] and ResViT (98.47%) [69] remain below the best CNN and hybrid alternatives. Strong hybrid/attentionized models narrow the gap, such as RanMerFormer achieves 99.17% [72], and Hybrid ViT-DNN 99.19% [73]. High-capacity CNN ensembles also report strong accuracy near 99% (e.g., NeuroNet19 at 99.3% [74]). However, many of these works omit F1, making robustness comparisons difficult.

Traditional single CNN architectures tend to underperform on the D2 (four-class, 3264 images), with reported accuracies such as 91.88% for a baseline CNN model [65] and 94.5% for VGG-16 [66]. Modern backbones are narrowing the gap (Xception 97.6/97.9 [63], EfficientNetB3 97.5% [64]) and even EfficientNet-B7 tops out around 97% on D2 [61].

Several studies report strong performance on the four-class dataset containing 4292 images, although the reported results vary widely. For instance, TumorAwareNet achieves 93.50% accuracy and a 91.25% F1-score [60], while a VGG16-based DCNN reports 96.67% accuracy [62], and EfficientNet-B7 reaches 96% accuracy [61]. In addition to the previously discussed CNN and Vision Transformer (ViT) baselines, several recent studies from 2024 to 2025 have evaluated models on Figshare, BraTS, or various mixed four-class MRI subsets. A majority-voting ensemble of deep CNNs reported exceptional accuracy scores of 99.8% and 95.8% on datasets comprising 3261 and 7023 images, respectively [3]. Another study combining ResNet101 with a channel-wise attention mechanism (CWAM) achieved 99.83% accuracy and a 99.27% F1-score [4]. A hybrid deep CNN approach evaluated three related tasks: tumor detection (99.53%), five-class tumor-type classification (93.81%), and tumor grading (98.56%) [8]. Furthermore, an ensemble-attention model combining MobileNetV3 and EfficientNetB7 achieved 98.94% accuracy on the Figshare dataset and 98.48% on BraTS19 [13]. Another method using a combination of deep learning and weight selection techniques reported an overall accuracy of approximately 95% [20]. Lastly, a study employing pixel and spatial-level machine learning with explainable AI (XAI) reported classification results attained F1-scores 99.84% with performance metrics [21].

### 4.16. Prototype Web Interface

A web-based prototype interface was developed to demonstrate the practical integration of the proposed joint classification and localization model. The interface allows a user to upload a brain MRI image and displays the predicted tumor class, localized tumor region, and corresponding interpretability visualization.

**Figure 40:**
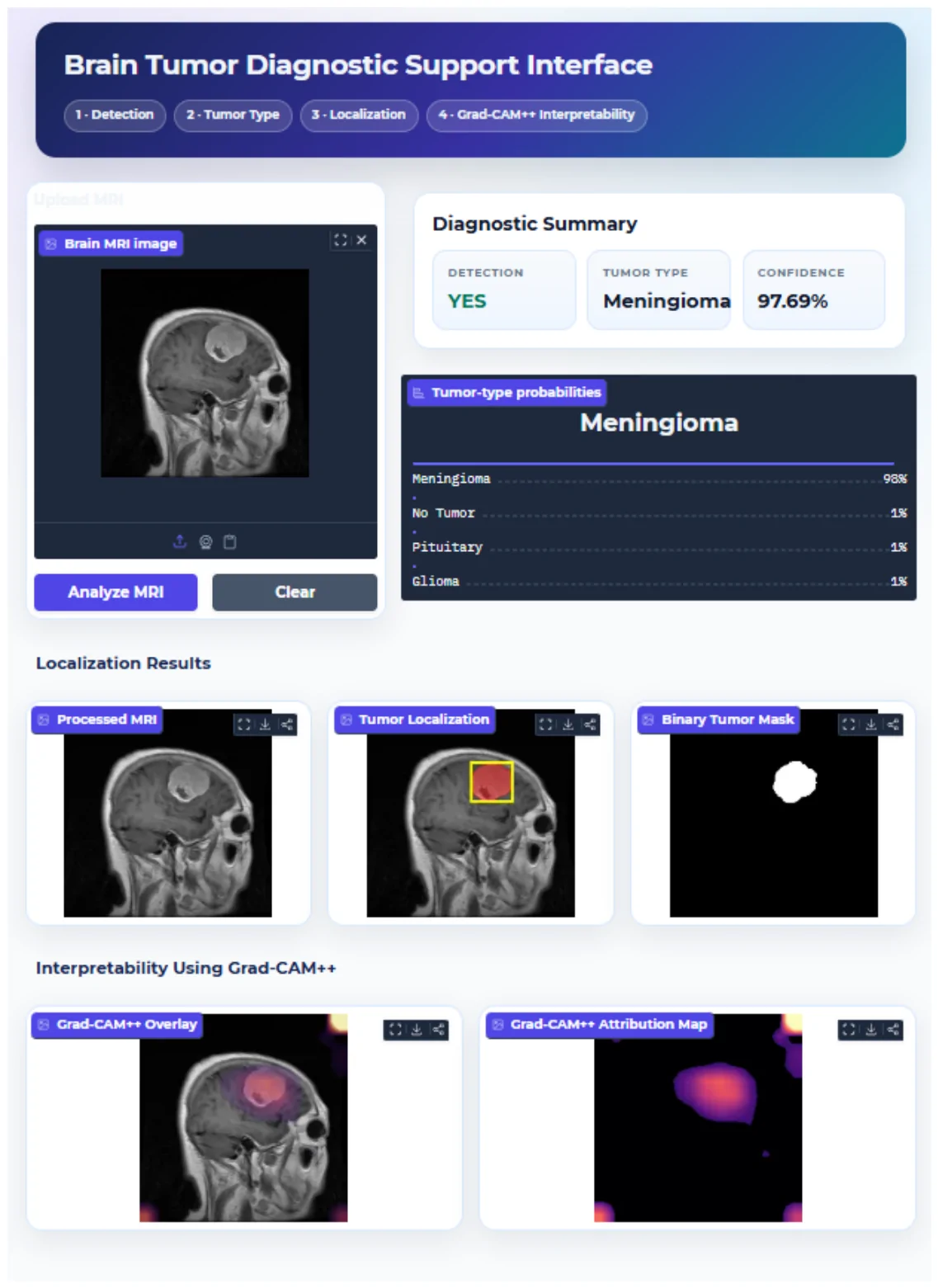
Prototype web interface for brain-tumor classification, localization, and interpretability using the proposed multitask model.

The interface is intended only as a research prototype to demonstrate the model workflow and should not be interpreted as a clinically deployed diagnostic system. The prototype interface is shown in Figure 40.

## 5. Discussion and Limitations

### 5.1. Discussion

This study achieved high accuracy on widely used public brain MRI datasets. This strong performance is consistent with many prior studies, where results above 98% are commonly reported for similar tumor classification tasks. At the same time, public MRI datasets can contain duplicated or near-duplicated images. This is especially likely in brain MRI because the same patient may have multiple scans over time that look very similar. If such images are split across training and testing, the model may appear better than it truly is due to data leakage. To reduce this risk, two complementary checks were applied before splitting: cryptographic hashing to remove exact duplicate images and perceptual hashing to remove near-duplicate images. This step helps make the evaluation more reliable and reduces the chance of overly optimistic results.

On the internal TEST set, the best classification accuracy was achieved by gated_fp_ens, which reached 99.33%. On the external hospital dataset (TEST2), the strongest performance was obtained by attn_fp_ens, which achieved 95.16% accuracy and a macro-F1 score of 0.9411. These findings suggest that strong performance on public benchmark data does not always translate equally across external clinical data, and they further support the presence of domain shift.

In comparison with other hybrid ensemble methods proposed in the literature, this framework has several distinct features. Many previous ensemble studies rely mainly on majority voting, averaging, or validation-based stacking. In contrast, this work compares different fusion strategies, namely concatenation, attention, and gating, in a controlled and unified setting. Moreover, unlike many earlier stacking-based studies, this framework also evaluates out-of-fold stacking, which is more careful from a data-leakage perspective. Another notable feature is the inclusion of calibration analysis, which is often ignored in earlier ensemble-based MRI classification studies.

Despite the very high internal testing accuracy (99%) achieved by the model, this finding should be cautiously regarded given its comparison to the overall evaluation process. The lower performance on the external dataset from the hospital (95%) suggests that there was a certain domain-shift problem and, therefore, the model did not fully generalize. It demonstrates how crucial the external validation of such results is. Therefore, the current findings should not be interpreted as evidence of clinical readiness, and further patient-level, multi-institutional, and prospective validation is required before clinical deployment.

### 5.2. Limitations

This study has several limitations. First, the datasets were collected from publicly available sources, and the ground-truth labels were not clinically re-verified, so some label noise may remain. Second, performance may vary across scanner vendors, field strengths, and acquisition protocols. Such differences can shift the image distribution and affect generalization.

Third, patient-level separation cannot be fully guaranteed because patient identifiers are not available in the public datasets. Although hash- and perceptual-hash-based deduplication removes exact and near-duplicate images, it cannot perfectly ensure that multiple scans from the same patient never appear across different splits. The external hospital evaluation provides additional evidence under domain shift; however, it does not establish clinical readiness, and broader patient-level and multi-institutional validation is still required.

## 6. Conclusions

This study developed an efficient pipeline for MRI-based brain-tumor classification using pretrained CNN- and ViT-based models, a lightweight modality fingerprint (FP) vector, and three fusion heads: concat, attention, and gated. The effects of FP features, activation ensembling (Mish, SiLU, and GELU), and two stacking strategies (VAL-driven and TRAIN-OOF) were evaluated using a large multi-source dataset.

The results show that the usefulness of FP features depends on the type of fusion head. FP consistently improves the attention head and also provides clear gains for the gated head on both TEST and TEST2. In contrast, its effect on the concat head is mixed, especially under external shift. Ensembling activation functions improves the attention head and can also help the gated head, particularly on TEST2, but it does not consistently benefit the concat head.

For meta-learning, the VAL-driven stacking variants remain competitive, but they do not outperform the best-performing fusion head on the internal TEST set. TRAIN-OOF stacking provides only limited additional benefit, likely because the base models are already strong. A drop in performance on TEST2 confirms the presence of domain shift. Among all tested models, attn_fp_ens showed the strongest robustness on TEST2, achieving the highest external accuracy and macro-F1. For the internal TEST set, the best classification accuracy was achieved by gated_fp_ens. Thus, the results suggest that the most suitable model depends on the target setting: gated_fp_ens is strongest for internal benchmark performance, whereas attn_fp_ens is more robust under external distribution shift. The attn_fp_ens model was further evaluated using 10-fold cross-validation to assess its stability.

From a deployment perspective, concat_nofp_single was the most efficient model and achieved the highest overall efficiency rank. Therefore, this study highlights an important practical trade-off: the most accurate model, the most robust model under domain shift, and the most efficient model are not the same. This difference is important for real-world deployment, where accuracy, generalization, and computational cost must all be considered together.

## Figures and Tables

**Table 1:** Summary of recent deep-learning methods for brain-tumor MRI classification and reported results (as stated by the authors).

Study/Theme	Model or Strategy	Reported Outcome (as Stated)	Why It Matters/Typical Limitation
Liu et al. (2025) [3]	Majority-vote ensemble of seven CNNs (ResNet, GoogLeNet, MobileNet, EfficientNet, etc.)	99.8% (in-domain); 95.8% (external)	Strong voting baseline; still does not address calibration explicitly, and generalization is evaluated on limited external settings [3].
Balamurugan et al. (2024) [4]	ResNet101 + channel-wise attention (CWAM)	99.83% accuracy (4-class)	Attention improves feature focus; typically trained/evaluated on a single dataset distribution [4].
Celik et al. (2024) [13]	Ensemble attention (MobileNetV3 + EfficientNetB7)	98.94% (Figshare); 98.48% (BraTS19)	Shows attention+ensemble benefit; calibration and cross-site clinical external testing are not central [13].
Babu Vimala et al. (2023) [2]	EfficientNetB0–B4 fine-tuning + Grad-CAM (CE-MRI Figshare; 3-class)	99.06% acc; 98.79% F1	Strong transfer-learning baseline; 3-class setting and limited cross-dataset validation [2].
Hosny et al. (2025) [15]	DenseNet121 + InceptionV3 ensemble + Grad-CAM (CE-MRI; 3-class)	99.02% acc; 98.86% F1; 98.11% mean (5-fold)	Includes CV + explainability; still not multi-source leakage-controlled training and no domain-shift hospital test [15].
Haque et al. (2025) [5]	Explainable stacking ensemble: EfficientNetB0, MobileNetV2, GoogLeNet, and Multi-level CapsuleNet; CatBoost meta-learner	97.81% F1 + 98.75% PR-AUC (M1); 98.32% F1 + 99.34% PR-AUC (M2)	Strong stacking over heterogeneous backbones on merged multi-source datasets; however, calibration metrics (ECE/Brier/TS) are not emphasized, and the stacking protocol is not clearly described as leakage-safe OOF [5].
Napa et al. (2026) [16]	BRAIN-META: 10 CNN–ViT hybrids + stacked meta-learners (Logistic Regression, XGBoost) trained on validation softmax outputs	97.10% accuracy (XGBoost); 97.03% (Logistic Regression)	Uses stacked meta-learning over CNN–Transformer hybrids, but stacking is validation-driven, evaluated on a single public dataset, and does not emphasize calibration metrics or clinical external testing [16].
Sankari et al. (2025) [17]	Hierarchical multi-scale ViT (HMSA-ViT) with explicit calibration reporting	98.7% accuracy; ECE = 0.023 (reported)	Focuses on a single architecture and does not provide a controlled multi-backbone fusion/head comparison or leakage-controlled multi-source merging with external hospital testing [17].
Ahmed et al. (2024) [18]	Hybrid ViT + GRU with XAI; hospital MRI data + 10-fold CV	Up to 98.97% accuracy (optimizer-dependent)	Uses real hospital data + XAI; but generally trained on a single local source and not a unified multi-source leakage-controlled training design [18].
Ahmed et al. (2025) [19]	SwRD: parallel Swin Transformer + ResNet50 feature fusion	99.79% acc; 100% CV (as reported)	Hybrid CNN–Transformer fusion aligns with the motivation; very high scores emphasize the need for leakage checks, external testing, and calibration reporting [19].
Gasmi et al. (2024) [20]	ViT + EfficientNetV2 ensembles; weighted averaging + GA weight tuning	Best reported ≈ 96.09% accuracy	Shows weight-optimized ensembles; still not focused on calibration metrics or external hospital evaluation [20].
Akter et al. (2024) [21]	Handcrafted statistical features with explainable classical ML classifiers	RF 99.84% ± 0.1% accuracy; several classifiers reaching 100%	Highlights low-cost handcrafted cues; however, it is not a deep-learning backbone approach and does not establish robustness under external hospital domain shift [21].
Singh et al. (2025) [22]	Dynamic ensemble with explainability; includes cross-dataset validation	99.4% test; 99.31% cross-dataset	Includes cross-dataset validation; still not a controlled, systematic comparison of fusion heads under one unified training protocol [22].

**Table 2:** Overview of the nine publicly available brain MRI datasets used to construct the merged dataset.

ID	Dataset (Short Name)	Classes	Size (Images)	Citation
S1	Maxwell Bernard multiclass MRI	4	16,269	[24]
S2	Figshare “brain_tumor_dataset”	3	3064	[25]
S3	BRISC2025	4	6000	[26,27]
S4	Nickparvar Brain-Tumor MRI	4	7200	[28]
S5	Mohamed Islam Houssam Brain-Tumor MRI	4	21,672	[29]
S6	Bhuvaji et al. Brain-Tumor Classification (MRI)	4	3160	[30]
S7	Tom Backert Brain-Tumor MRI Data	4	7153	[31]
S8	Hira Brain-Tumor MRI	4	12,064	[32]
S9	Jamil Multiclass Brain-Tumor MRI	4	1137	[33]
Total			77,719	

S2 contains three tumor classes and does not include the no-tumor class.

**Table 3:** Number of images per split and class after leakage-safe grouping by MD5 hash.

Split	Glioma	Meningioma	No-Tumor	Pituitary	Total
Train	4388	3292	4880	3035	15,595
Val	878	658	976	607	3119
Test	585	439	651	405	2080
All	5851	4389	6507	4047	20,794

**Table 4:** Class-wise distribution of the external test set (TEST2).

Class	Number of Images
Glioma	65
Meningioma	271
Pituitary Tumor	128
No Tumor	218
Total	682

**Table 5:** Components of the Fingerprint Descriptor.

#	Component and Definition (on Normalized Grayscale $\tilde{x}\in {\left[0,1\right]}^{H\times W}$; N=HW)
1	Normalized histogram (64 bins): $h_{j}=n_{j}/N,\sum_{j=1}^{64}h_{j}=1$, where $n_{j}$ counts pixels in bin *j* of the 8-bit histogram rebinned to B=64.
2	Mean intensity: $\mu =\frac{1}{N}\sum_{p=1}^{N}{\tilde{x}}_{p}$.
3	Standard deviation (population): $\sigma =\sqrt{\frac{1}{N}\sum_{p=1}^{N}{\left({\tilde{x}}_{p}-\mu \right)}^{2}}$.
4	Skewness (adjusted Fisher–Pearson): $m_{k}=\frac{1}{N}\sum_{p}{\left({\tilde{x}}_{p}-\mu \right)}^{k},g_{1}=\frac{m_{3}}{m_{2}^{3/2}},\gamma =\frac{\sqrt{N(N-1)}}{N-2}g_{1},N>2$ [42].
5	Kurtosis (unbiased excess): $g_{2}=\frac{m_{4}}{m_{2}^{2}}-3,\kappa =\frac{N-1}{\left(N-2)(N-3\right)}\left[\left(N+1\right)g_{2}+6\right],N>3$ [42].
6	Shannon entropy of histogram: $H=-\sum_{j=1}^{B}h_{j}\log\left(h_{j}+\varepsilon \right)$ with $\varepsilon =10^{-8}$ [43].
7	Otsu foreground ratio: $t^{⋆}$ is the Otsu threshold from the grayscale (via h); $\rho =\frac{1}{N}\left|\{p:x_{p}\geq t^{⋆}\}\right|$ [44].
8	Final feature vector: $r\left(x\right)=\left[h_{1},\ldots ,h_{B},\mu ,\sigma ,\gamma ,\kappa ,H,\rho \right]\in R^{B+6}$; for B=64, dim=70.

**Table 6:** Training parameter selection with candidate choices and final selected settings.

Parameter	Candidates/Range	Selected Value
Classifier head	Logistic Regression, LinearSVC, SGD-Logistic, RidgeClassifier, Perceptron, Passive-Aggressive, GaussianNB, LDA, QDA, Nearest Centroid, kNN, Random Forest, ExtraTrees, Gradient Boosting, XGBoost, LightGBM, CatBoost, and MLP-based neural Classifier	MLP-based neural Classifier
Optimizer	Adam, AdamW, RAdam, AdaBelief, SGD, SGD-Nesterov	AdamW
Learning rate	$2\times 10^{-4}$, $3\times 10^{-4}$, $4\times 10^{-4}$, $5\times 10^{-4}$	$2\times 10^{-4}$
Weight decay	$1\times 10^{-5}$, $5\times 10^{-5}$, $1\times 10^{-4}$, $3\times 10^{-4}$	$5\times 10^{-5}$
Epochs	20, 30, 40, 50	40
Batch size	16, 32, 64	32
Activation function	ReLU, LeakyReLU, SiLU, Mish, GELU	Mish, SiLU, and GELU
Loss function	Cross-entropy, label-smoothed cross-entropy, focal loss	Label-smoothed cross-entropy
Label smoothing	0.06, 0.08, 0.10	0.10
MixUp	MixUp with α∈{0.2,0.4,0.6}	α=0.4, applied to 50% of mini-batches
EMA decay	0.99, 0.995, 0.999	0.999
Learning-rate scheduler	Step decay, cosine decay, warmup–cosine decay	Warmup–cosine decay
Warmup fraction	0.03, 0.05, 0.10	0.05
Minimum learning rate	$1\times 10^{-6}$, $5\times 10^{-6}$, $1\times 10^{-5}$	$1\times 10^{-6}$
Determinism	Fixed seed only; fixed seed with deterministic cuDNN	Fixed global seeds with deterministic cuDNN

**Table 7:** Compact ablation on VAL/TEST/TEST2.

Name	T	VAL Acc	TEST Acc	TEST Acc CI	TEST F1	TEST ECE	TEST Brier	TEST Det Acc	TEST2 Acc	TEST2 F1	TEST2 Det Acc
concat_fp_single	0.40	0.9936	0.9928	[0.9889,0.9962]	0.9923	0.0018	0.0026	0.9986	0.8768	0.8744	0.9106
concat_fp_ens	0.40	0.9929	0.9923	[0.9885,0.9962]	0.9918	0.0031	0.0029	0.9981	0.8783	0.8720	0.9120
concat_nofp_single	0.45	0.9926	0.9928	[0.9889,0.9962]	0.9924	0.0039	0.0025	0.9986	0.8886	0.8817	0.9223
concat_nofp_ens	0.45	0.9926	0.9928	[0.9889,0.9962]	0.9924	0.0038	0.0027	0.9981	0.8827	0.8782	0.9179
attn_fp_single	0.50	0.9894	0.9889	[0.9841,0.9933]	0.9882	0.0039	0.0048	0.9971	0.9267	0.9143	0.9487
attn_fp_ens	0.50	0.9920	0.9899	[0.9856,0.9938]	0.9896	0.0037	0.0042	0.9976	0.9516	0.9411	0.9707
attn_nofp_single	0.55	0.9894	0.9880	[0.9832,0.9923]	0.9875	0.0036	0.0047	0.9971	0.8988	0.8900	0.9252
attn_nofp_ens	0.50	0.9897	0.9875	[0.9822,0.9918]	0.9869	0.0039	0.0044	0.9976	0.9194	0.9091	0.9428
gated_fp_single	0.40	0.9933	0.9918	[0.9880,0.9957]	0.9911	0.0026	0.0029	0.9990	0.8768	0.8709	0.9091
gated_fp_ens	0.40	0.9923	0.9933	[0.9899,0.9966]	0.9928	0.0048	0.0029	0.9990	0.9047	0.9013	0.9326
gated_nofp_single	0.40	0.9939	0.9909	[0.9865,0.9947]	0.9903	0.0038	0.0031	0.9981	0.8842	0.8768	0.9238
gated_nofp_ens	0.40	0.9929	0.9913	[0.9875,0.9952]	0.9909	0.0032	0.0031	0.9981	0.8856	0.8763	0.9282
super_bestheads	0.40	0.9939	0.9928	[0.9889,0.9962]	0.9923	0.0049	0.0028	0.9986	0.9003	0.8935	0.9326
stack_lr_full_top2_fp	1.00	0.9929	0.9923	[0.9885,0.9962]	0.9918	0.0030	0.0032	0.9986	0.8944	0.8902	0.9296
stack_lr_full_top3_fp	1.00	0.9945	0.9928	[0.9889,0.9962]	0.9924	0.0039	0.0031	0.9990	0.9091	0.9026	0.9384
stack_lr_full_top2_nofp	1.00	0.9933	0.9923	[0.9885,0.9957]	0.9918	0.0017	0.0032	0.9986	0.8842	0.8720	0.9252
stack_lr_full_top3_nofp	1.00	0.9933	0.9918	[0.9880,0.9957]	0.9915	0.0031	0.0035	0.9981	0.8900	0.8773	0.9340
stack_lr_full_top3_mixed	1.00	0.9942	0.9928	[0.9889,0.9962]	0.9924	0.0032	0.0033	0.9986	0.9032	0.8940	0.9413
stack_oof_full_top2_single_fp	1.00	0.9913	0.9899	[0.9856,0.9942]	0.9891	0.0052	0.0039	0.9986	0.8886	0.8804	0.9311
stack_oof_full_top2_ens_fp	1.00	0.9913	0.9899	[0.9856,0.9942]	0.9891	0.0066	0.0040	0.9981	0.8944	0.8852	0.9311
stack_oof_full_top3_single_fp	1.00	0.9910	0.9889	[0.9841,0.9933]	0.9881	0.0065	0.0040	0.9981	0.8959	0.8841	0.9355
stack_oof_full_top3_ens_fp	1.00	0.9913	0.9894	[0.9851,0.9938]	0.9886	0.0054	0.0041	0.9976	0.8974	0.8866	0.9370
stack_oof_full_top2_single_nofp	1.00	0.9920	0.9904	[0.9861,0.9947]	0.9896	0.0059	0.0038	0.9986	0.8842	0.8765	0.9208
stack_oof_full_top2_ens_nofp	1.00	0.9917	0.9899	[0.9856,0.9942]	0.9891	0.0052	0.0040	0.9986	0.8783	0.8686	0.9223
stack_oof_full_top3_single_nofp	1.00	0.9913	0.9904	[0.9861,0.9947]	0.9896	0.0059	0.0038	0.9986	0.8856	0.8755	0.9223
stack_oof_full_top3_ens_nofp	1.00	0.9913	0.9904	[0.9861,0.9947]	0.9896	0.0052	0.0040	0.9986	0.8798	0.8704	0.9208
stack_oof_full_top3_single_mixed	1.00	0.9920	0.9904	[0.9861,0.9947]	0.9896	0.0058	0.0038	0.9986	0.8959	0.8856	0.9384
stack_oof_full_top3_ens_mixed	1.00	0.9913	0.9899	[0.9856,0.9942]	0.9891	0.0050	0.0040	0.9976	0.9003	0.8888	0.9413

Variant definitions for Table 7. concat|attn|gated = fusion-head type (MLP concat head, attention fusion, or gated fusion). fp = histogram-based modality fingerprint vector appended or injected. nofp = no fingerprint features. single = best single activation selected by validation accuracy. ens = within-head activation ensemble over ENSEMBLE_ACTS (Mish, SiLU, GELU) with validation-weighted logit averaging. super_bestheads = best-performing base variant from each head type (concat, attention, gated) combined by validation-weighted logits. stack_lr_full_topK_{fp|nofp|mixed} = VAL-driven stacking using a multinomial logistic-regression meta-learner trained on VAL using concatenated calibrated probabilities from selected top-K base variants. stack_oof_full_topK_{…} = TRAIN-only OOF stacking, where base heads are trained inside each fold and used to generate OOF meta-features; the meta-learner is trained on OOF predictions. _single (in OOF rows) = best single activation per head selected within each fold using inner-validation performance. _ens (in OOF rows) = validation-weighted activation ensemble inside each base head. T (temperature) = scaling value learned to improve probability calibration by minimizing cross-entropy. VAL-driven stacking (stack_lr_*) = temperature calibration is learned on VAL before generating calibrated probabilities for the meta-learner. TRAIN-only OOF stacking (stack_oof_*) = temperature calibration is learned only from each fold’s inner-validation split before generating OOF features. Mixed set = top3_mixed selected using VAL ranking.

**Table 8:** Final model with synchronized 10-fold CV on TRAIN (variant: attn_fp_ens; activations: Mish, SiLU, GELU).

OOF Acc	OOF MacroF1	VAL Acc	TEST Acc	TEST MacroF1	TEST2 Acc
0.9829	0.9821	0.9885	0.9904	0.9897	0.9252

**Table 9:** Single-backbone baseline comparison on the internal TEST set.

Model	Macro-F1
Swin-S	0.9091
ViT-B/16	0.9192
ConvNeXt-B	0.9246
DINOv3-B/16	0.9251
EfficientNetV2-M	0.9454
DenseNet201	0.9531
gated_fp_ens	0.9928

**Table 10:** Dataset-source prediction using the five lowest-intensity histogram bins.

Feature Region	Accuracy (%)	Balanced Accuracy (%)
Outer 10% border	33.05	16.89
Whole image	34.12	16.95

**Table 11:** Mean performance across 28 model variants using raw and skull-stripped/background-cropped images. Δ represents the change after skull stripping (skull-stripped minus raw).

Dataset	Raw Acc.	Skull Acc.	Δ Acc.	Raw F1	Skull F1	Δ F1
Internal TEST	99.10%	98.02%	−1.08 pp	99.04%	97.91%	−1.13 pp
TEST2	89.49%	93.77%	+4.28 pp	88.63%	91.99%	+3.36 pp

**Table 12:** Source-wise leave-one-dataset-source-out (LODO) performance of gated_fp_ens.

Held-Out Source	Test Images	Accuracy	Balanced Acc.	Macro-F1
S1	16,269	97.76%	97.42%	97.70%
S2	3064	99.93%	99.93%	99.92% *
S3	6000	99.05%	98.83%	98.96%
S4	7200	99.92%	99.92%	99.92%
S5	21,672	84.68%	86.48%	83.71%
S6	3160	99.56%	99.58%	99.60%
S7	7153	99.94%	99.95%	99.94%
S8	12,064	98.96%	99.07%	98.96%
S9	1137	99.91%	99.91%	99.91%

* S2 does not contain the no-tumor class; therefore, macro-F1 is reported over the classes present in that source.

**Table 13:** Quantitative comparison of the evaluated XAI techniques.

XAI Method	Tumor-SizedDice ↑	Tumor-SizedIoU ↑	Top-10Precision ↑	EnergyLift ↑	PixelAP ↑	FaithfulnessGain ↑	DiffuseMap Flag	Final Remark
Grad-CAM++	0.434	0.324	0.266	4.735	0.464	0.240	No	Selected method
LayerCAM	0.434	0.324	0.266	4.718	0.463	0.236	Yes	Good, but relatively diffuse
Grad-CAM	0.434	0.324	0.266	4.735	0.464	0.169	No	Good supporting method
HiResCAM	0.200	0.143	0.132	3.270	0.229	0.171	No	Weaker localization
Score-CAM	0.259	0.188	0.205	1.807	0.292	0.281	Yes	Broad heatmap, less focused
XGrad-CAM	0.200	0.143	0.132	3.270	0.229	0.170	No	Moderate
Ablation-CAM	0.222	0.158	0.164	2.067	0.262	0.247	Yes	Diffuse
Integrated Gradients	0.160	0.092	0.090	3.261	0.125	0.213	No	Scattered attribution
Patch Occlusion	0.213	0.160	0.100	3.613	0.233	0.232	Yes	Diffuse and coarse
GradientSHAP	0.150	0.086	0.083	3.000	0.112	0.206	No	Lower localization
LIME	0.198	0.151	0.111	2.282	0.207	0.393	Yes	Excluded by quality screen

Note: Higher values are better for Dice, IoU, Top-10 Precision, Energy Lift, Pixel AP, and Faithfulness Gain. “Diffuse map flag” indicates that the method highlighted a relatively broad area rather than a focused lesion-related region.

**Table 14:** Overall performance of attn_fp_ens on TEST2.

Metric	Value
Accuracy	0.9516
95% CI	[0.9370, 0.9663]
Macro-F1	0.9411
Macro-AUROC	0.9779
Macro-PR-AUC	0.9659
ECE	0.0342
Brier score	0.0249
Misclassified Samples	33/682

**Table 15:** Class-wise performance on TEST2.

Class	Support	Correct	Wrong	Accuracy	Error Rate	Precision	F1
Glioma	65	62	3	0.9538	0.0462	0.8267	0.8857
Meningioma	271	260	11	0.9594	0.0406	0.9630	0.9612
Notumor	218	208	10	0.9541	0.0459	0.9541	0.9541
Pituitary	128	119	9	0.9297	0.0703	1.0000	0.9636

**Table 16:** Top confusion pairs on TEST2.

True Class	Predicted Class	Count
Meningioma	Notumor	6
Meningioma	Glioma	5
Notumor	Glioma	5
Notumor	Meningioma	5
Pituitary	Notumor	4
Glioma	Meningioma	3
Pituitary	Glioma	3
Pituitary	Meningioma	2

**Table 17:** Inference time of the six frozen feature-extraction backbones.

Input	No TTA (ms/Image)	Flip TTA (ms/Image)
Raw	87.73	165.11
Skull-stripped	82.99	157.94

**Table 18:** Head-level efficiency comparison of the base heads and meta/ensembling methods. The common six-backbone feature-extraction cost is excluded and reported separately in Table 17.

Variant	Train (s)	Params (M)	Head Infer. (ms/img)	Efficiency	Rank
concat_nofp_single	116.1	6.69	0.000686	1.000	1
concat_fp_single	117.4	6.96	0.000842	0.935	2
gated_nofp_single	160.5	12.90	0.001867	0.750	3
gated_fp_single	149.2	13.44	0.001933	0.745	4
concat_fp_ens	117.8	20.87	0.002197	0.706	5
concat_nofp_ens	116.2	20.06	0.002292	0.705	6
attn_nofp_single	361.4	8.27	0.004968	0.657	7
attn_fp_single	425.4	9.58	0.004317	0.648	8
stack_oof_full_top2_single_nofp	875.2	19.59	0.002553	0.573	9
gated_nofp_ens	161.1	38.70	0.004996	0.568	10
attn_nofp_ens	362.3	24.80	0.013079	0.561	11
gated_fp_ens	149.4	40.33	0.005194	0.561	12
stack_oof_full_top2_single_fp	892.1	20.40	0.002775	0.559	13
attn_fp_ens	426.3	28.75	0.014206	0.533	14
super_bestheads	703.3	29.44	0.007026	0.502	15
stack_lr_full_top2_nofp	277.4	58.76	0.007288	0.453	16
stack_lr_full_top2_fp	267.3	61.20	0.007390	0.445	17
stack_oof_full_top2_ens_nofp	870.2	58.76	0.007288	0.357	18
stack_oof_full_top2_ens_fp	901.4	61.20	0.007390	0.341	19
stack_oof_full_top3_single_mixed	1908.6	29.44	0.007026	0.306	20
stack_oof_full_top3_single_nofp	2100.0	27.85	0.007521	0.279	21
stack_lr_full_top3_nofp	639.7	83.56	0.020367	0.274	22
stack_oof_full_top3_single_fp	2149.6	29.98	0.007092	0.264	23
stack_lr_full_top3_mixed	705.2	88.32	0.021398	0.244	24
stack_lr_full_top3_fp	693.5	89.95	0.021596	0.239	25
stack_oof_full_top3_ens_nofp	1951.6	83.56	0.020367	0.060	26
stack_oof_full_top3_ens_mixed	1906.5	88.32	0.021398	0.048	27
stack_oof_full_top3_ens_fp	2159.7	89.95	0.021596	0.000	28

**Table 19:** Bowker test vs. gated_fp_ens on the TEST set. $p_{\mathrm{Holm}}$ controls FWER at α=0.05 across m=27 comparisons.

Variant	*p*	$p_{\mathrm{Holm}}$	$\chi^{2}$	df	Sig.
attn_fp_ens	0.2333	1.0000	6.833	5	
attn_fp_single	0.3971	1.0000	4.067	4	
attn_nofp_ens	0.2116	1.0000	7.123	5	
attn_nofp_single	0.6183	1.0000	3.533	5	
concat_fp_ens	0.1215	1.0000	7.286	4	
concat_fp_single	0.1546	1.0000	6.667	4	
concat_nofp_ens	0.2998	1.0000	3.667	3	
concat_nofp_single	0.1063	1.0000	6.111	3	
gated_fp_single	0.3916	1.0000	3.000	3	
gated_nofp_ens	0.6387	1.0000	2.533	4	
gated_nofp_single	0.3796	1.0000	4.200	4	
stack_lr_full_top2_fp	0.1116	1.0000	6.000	3	
stack_lr_full_top2_nofp	0.3056	1.0000	3.619	3	
stack_lr_full_top3_fp	0.0266	0.7172	11.000	4	
stack_lr_full_top3_mixed	0.1215	1.0000	7.286	4	
stack_lr_full_top3_nofp	0.1718	1.0000	5.000	3	
stack_oof_full_top2_ens_fp	0.5037	1.0000	3.333	4	
stack_oof_full_top2_ens_nofp	0.5823	1.0000	1.952	3	
stack_oof_full_top2_single_fp	0.5037	1.0000	3.333	4	
stack_oof_full_top2_single_nofp	0.7212	1.0000	1.333	3	
stack_oof_full_top3_ens_fp	0.6747	1.0000	2.333	4	
stack_oof_full_top3_ens_mixed	0.9293	1.0000	0.867	4	
stack_oof_full_top3_ens_nofp	0.7603	1.0000	1.867	4	
stack_oof_full_top3_single_fp	0.5358	1.0000	3.133	4	
stack_oof_full_top3_single_mixed	0.7603	1.0000	1.867	4	
stack_oof_full_top3_single_nofp	0.7603	1.0000	1.867	4	
super_bestheads	0.2255	1.0000	5.667	4	

**Table 20:** Unified comparison of prior works on MRI brain-tumor classification (2024–2025)

Model	Categories	Images	Accuracy	F1 Score	Year	Ref.
CNN Ensemble (Majority Voting)	4	3261; 7023	99.8%; 95.8%	—	2025	[3]
ResNet-101 + CWAM	4	7023; 3264	99.83%	99.27%	2024	[4]
Hybrid CNN (Detection/5-Class/Grading)	2/5	—	99.53%; 93.81%; 98.56%	—	2024	[8]
Attention Ensemble (MobileNetV3 + EfficientNet-B7)	3	Figshare; BraTS19	98.94%; 98.48%	—	2024	[13]
Combined Deep Learning with Weight Selection	—	—	95%	—	2024	[20]
Pixel/Spatial Feature ML with XAI	3/4	—	99.845%	—	2024	[21]
TumorAwareNet (SCDA + NSVC)	4	4292	93.50	91.25	2024	[60]
EfficientNet B7	4	3264; 4292	97%, 96%	96%, 97%	2024	[61]
VGG16-based DCNN	4	4292	96.67%	—	2024	[62]
Xception	4	3264	97.6%	97.9%	2024	[63]
EfficientNetB3	4	3264	97.5%	—	2024	[64]
CNN	4	3264	91.88%	—	2024	[65]
VGG-16	4	3264	94.5	94.5	2024	[66]
CNN-ML Ensemble	4	7022	96	95.91	2024	[67]
FTVT-l16	4	7023	98.70%	—	2024	[68]
ResViT	4	7023	98.47%	—	2024	[69]
ViT-B/16	4	7023	91.36%	—	2024	[70]
ViT-B/16 (Patterned-GridMask)	4	7023	97.74%	97.75%	2024	[71]
RanMerFormer	4	7023	99.17%	—	2024	[72]
Hybrid ViT-DNN	4	7023	99.19%	—	2024	[73]
NeuroNet19	4	7023	99.3%	—	2024	[74]
BT-CNN	4	7023	96.06%	—	2024	[75]
InceptionV4	4	7022	98.7%	—	2024	[76]
gated_fp_ens (present study; TEST)	4	20,794 (total)	99.33%	99.28%	2026	—

## Data Availability

The merged multi-source dataset (MMBMD) is available at Kaggle: https://www.kaggle.com/datasets/faysal202114023/majib-multi-source-brain-mri-dataset-mmbmd (accessed on 29 August 2026). The de-duplicated dataset is available at Kaggle: https://www.kaggle.com/datasets/faysal202114023/de-duplicated-brain-mri-dataset (accessed on 29 August 2026). The external hospital dataset (NINS-EXT/TEST2) is available at Kaggle: https://www.kaggle.com/datasets/faysal202114023/majib-et-al-external-test-set-nins-ext (accessed on 29 August 2026). The implementation and full experimental pipeline are available on GitHub: https://github.com/faysalahmed23/-Brain-Tumor-Detection-and-Classification- (accessed on 29 August 2026).
